# Circulating Long Non-Coding RNAs LINC00324 and LOC100507053 as Potential Liquid Biopsy Markers for Esophageal Squamous Cell Carcinoma: A Pilot Study

**DOI:** 10.3389/fonc.2022.823953

**Published:** 2022-02-14

**Authors:** Uttam Sharma, Tushar Singh Barwal, Akanksha Khandelwal, Manjit Kaur Rana, Amrit Pal Singh Rana, Karuna Singh, Aklank Jain

**Affiliations:** ^1^ Department of Zoology, Central University of Punjab, Bathinda, India; ^2^ Department of Biochemistry, Central University of Punjab, Bathinda, India; ^3^ Department of Pathology/Laboratory Medicine, All India Institute of Medical Sciences, Bathinda, India; ^4^ Department of Surgery, Baba Farid University of Health Sciences, Faridkot, India; ^5^ Department of Radiotherapy, Advanced Cancer Institute, Bathinda Affiliated with Baba Farid University of Health Sciences, Faridkot, India

**Keywords:** esophageal squamous cell carcinoma, ESCC, long non-coding RNA, LncRNA, next generation sequencing, biomarkers

## Abstract

**Background:**

Despite the availability of advanced technology to detect and treat esophageal squamous cell carcinoma (ESCC), the 5-year survival rate of ESCC patients is still meager. Recently, long non-coding RNAs (lncRNAs) have emerged as essential players in the diagnosis and prognosis of various cancers.

**Objective:**

This pilot study focused on identifying circulating lncRNAs as liquid biopsy markers for the ESCC.

**Methodology:**

We performed next-generation sequencing (NGS) to profile circulating lncRNAs in ESCC and healthy individuals’ blood samples. The expression of the top five upregulated and top five downregulated lncRNAs were validated through quantitative real-time PCR (qRT-PCR), including samples used for the NGS. Later, we explored the diagnostic/prognostic potential of lncRNAs and their impact on the clinicopathological parameters of patients. To unravel the molecular target and pathways of identified lncRNAs, we utilized various bioinformatics tools such as lncRnome, RAID v2.0, Starbase, miRDB, TargetScan, Gene Ontology, and KEGG pathways.

**Results:**

Through NGS profiling, we obtained 159 upregulated, 137 downregulated, and 188 neutral lncRNAs in ESCC blood samples compared to healthy individuals. Among dysregulated lncRNAs, we observed *LINC00324 significantly upregulated* (2.11-fold; *p-value* = 0.0032) and *LOC100507053* significantly downregulated (2.22-fold; *p-value* = 0.0001) in ESCC patients. Furthermore, we found *LINC00324* and *LOC100507053* could discriminate ESCC cancer patients’ from non-cancer individuals with higher accuracy of Area Under the ROC Curve (AUC) = 0.627 and 0.668, respectively. The Kaplan-Meier and log-rank analysis revealed higher expression levels of *LINC00324* and lower expression levels of *LOC100507053* well correlated with the poor prognosis of ESCC patients with a Hazard ratio of *LINC00324* = 2.48 (95% CI: 1.055 to 5.835) and Hazard ratio of *LOC100507053* = 4.75 (95% CI: 2.098 to 10.76)]. Moreover, we also observed lncRNAs expression well correlated with the age (>50 years), gender (Female), alcohol, tobacco, and hot beverages consumers. Using bioinformatics tools, we saw miR-493-5p as the direct molecular target of *LINC00324* and interacted with the MAPK signaling pathway in ESCC pathogenesis.

**Conclusion:**

This pilot study suggests that circulating *LINC00324* and *LOC100507053* can be used as a liquid biopsy marker of ESCC; however, multicentric studies are still warranted.

## Introduction

As per the recent GLOBOCAN 2020 data, esophageal cancer (EC) is the eighth most common cancer and the sixth leading cause of death globally (1). Moreover, a hospital-based analysis of 2088 patients in Northern India revealed esophageal cancer as the fourth (10.39%) most prevalent cancer (2). Because of the substantial increase in the incidence and mortality rates along with the decrease in the 5-year survival rate of EC (~18%), esophageal cancer has become a severe global public health challenge (3). Based on EC histology, it is classified into adenocarcinoma (AC) and esophageal squamous cell carcinoma (ESCC). Distinctively, ESCC represents 90%, while AC represents 10% of total EC cases worldwide (4). In line with this, our previous hospital-based study demonstrated 96.5% of esophagus tumors were squamous cell carcinoma, whereas 1.7% were adenocarcinoma (2). Despite the higher percentage of ESCC, its etiology remains poorly understood due to its heterogeneous nature. ESCC originates from the stratified squamous epithelial lining of the esophagus, which undergoes continuous proliferation followed by abnormal growth and improper differentiation of squamous cells. Currently, conventional diagnostic techniques like computed tomography (CT), tissue biopsy, and endoscopic ultrasonography suffer from severe limitations like a highly invasive nature, late detection, and high cost (5). Furthermore, its prognosis remains poor even after chemoradiotherapy and surgery of ESCC patients. Therefore, it is imperative to identify blood-based minimally invasive agents, which can be used as potential diagnostic and prognostic markers for ESCC patients.

Recent scientific investigation revealed that non-coding RNAs (ncRNAs) hold ~98% of the human genome (6). Typically, ncRNAs are divided into housekeepers and regulatory ncRNAs. Housekeeper ncRNAs are commonly referred to as transfer RNAs, ribosomal RNAs, small cytoplasmic RNAs, and small nuclear RNAs (7). The regulatory ncRNAs are broadly subcategorized into microRNAs (miRNAs, 18–25 nucleotides), small interfering RNAs (siRNAs, <200 nucleotides), piwi-interacting RNAs (piRNAs, <200 nucleotides), and long non-coding RNAs (lncRNAs, >200 nucleotides) (7). Among them, lncRNAs play a critical role in maintaining the cellular homeostasis of the biological system and modulating the hallmarks of cancers such as cell proliferation, migration, invasion, and epithelial-mesenchymal transition (EMT) (7, 8). Moreover, recent studies have validated the tissue/stage-specific expression of various lncRNAs, making it an ideal diagnostic and prognostic marker for several other tumors (7–10). Furthermore, lncRNAs possess an inherent ability to interact with RNA, DNA, and proteins, which facilitates its potential as therapeutic target molecule (6–8, 10–12). Interestingly, the previous transcriptome analysis also reported various cancer-associated lncRNAs (13–21) and among them, *PCA3* lncRNA is approved by FDA for clinical use of prostate cancer. Unfortunately, the blood-based biopsy marker and its comprehensive profiling and validation of clinically relevant lncRNA in the context of ESCC is still missing. Thus, the identification of novel lncRNA-based liquid biopsy biomarkers for the early diagnosis of ESCC will have significant clinical benefits. Therefore, we have designed a pilot study to investigate the potential circulating lncRNAs biomarker in ESCC patients’ blood samples compared to healthy individuals.

Overall, our pilot study addressed the gap in the clinical settings for the diagnosis/prognosis of ESCC through a non-invasive approach using a circulating lncRNA marker. For this, we utilized high throughput NGS technology to screen the circulating dysregulated lncRNAs in ESCC patients. By the qRT-PCR and Receiver Operating Characteristic (ROC) curve, we validated and established the diagnostic efficiency of *LINC00324* and *LOC100507053* in ESCC patients. Further, based on the follow-up of the ESCC patients, we estimated the prognostic efficiency of the *LINC00324* and *LOC100507053* using the Kaplan-Meier survival plot and log-rank analysis. We observed various molecular cues interacting with the *LINC00324* and *LOC100507053*, which could be a pivotal molecule regulating the expression of lncRNAs.

## Methodology

### Clinical Sample Collection

The present study was conducted in accordance with the Declaration of Helsinki and approved by the Ethics Review Board of our institution (ERB/UCER/2019/4/17 and CUPB/IEC/2018/12). ESCC patients were diagnosed by the pathologists through histopathological examination of tissue biopsies according to the Union for International cancer control (UICC) and American Joint Committee on Cancer (AJCC) TNM staging 8th edition. The peripheral blood samples (3 ml) were collected from freshly diagnosed ESCC patients (Age≥18 years) and healthy individuals (Age≥18 years) by the phlebotomist in the Tempus Blood RNA Tube (Cat. No. 4342792; Applied Biosystems, CA, USA) from April 2019 to July 2020. The patients’ complete follow-up and medical records, including age, gender, consumption of tobacco smoking, alcohol, hot beverages per day, tumor grade, and TNM stages, were collected.

The representative ESCC sample size was calculated using an online available sample size calculator (https://clincalc.com/Stats/SampleSize.aspx). We used a discovery cohort of 4 ESCC patients of different TNM stages (Age range = 50-60) and 4 healthy individuals as control (Age range = 50-60) for NGS. The results were further validated in n = 100 samples (50 ESCC and 50 healthy individuals as control), including samples used in NGS. Additionally, we curated the expression profiling data of esophageal cancer using the TCGA database GEPIA (http://gepia.cancer-pku.cn/) to support our experimental data. The clinical information of all the patients is listed in **Table 1**.

**Table 1:** Correlation of *LINC00324* and LOC100507053 expression with lifestyle status and clinicopathological characteristics in 50 ESCC patients.

Characteristics		*LncRNA* expression group^a^		p-value^b^
		High expression	Low expression	
**Age (in years)**	18 ≤ 50	10	11	0.9634
	> 50	14	15	
**Gender**	Male	13	13	>0.9999
	Female	12	12	
**Tobacco smoking**	Yes	10	11	0.9634
	No	14	15	
**Alcohol consumption**	Yes	13	14	0.9819
	No	11	12	
**Hot beverages (Tea/Coffee)**	Yes	16	16	0.8446
	No	8	9	
**Tumor grade**	Well (G1)	7	7	>0.9999
	Moderate (G2)	5	5	
	Poor (G3)	6	6	
	Unknown (GX)	7	7	
**TNM stage**	Stage (I + II)	20	21	0.8136
	Stage (III + IV)	4	5

a Median expression level was used as a cutoff to divide the 50 patients into high and low group.

b Chi-square test.

### Next-Generation Sequencing and Data Processing

To profile the lncRNAs in ESCC patients, we outsourced the blood samples for next-generation sequencing. The company isolated total RNA from ESCC patients of different stages (n = 4) and healthy individuals’ (n = 4) blood samples using their customized kit of Agilent Technologies. To ensure the good quality of the samples for sequencing, Nanodrop UV-Vis Spectrophotometer 2000cc (Thermo Fisher Scientific, CA, USA) and Agilent 2100 Bioanalyzer (Agilent Technologies, CA, USA) were used to detect the purity, concentration, and integrity of the RNA samples respectively. Later, paired-end libraries from normal and ESCC blood samples were generated using NEBNext Small RNA Sample Library Prep kit (NEB, USA) and sequenced on the Illumina HiSeq 2000 platform (Illumina Inc., San Diego, CA, USA). The raw reads’ quality control was assessed using the FastQC program (version 0.11.8) (22). The low-quality reads (Phred quality score < 30) were discarded, and the remaining were considered as “high-quality reads”, whose adaptor sequences were removed using Cutadapt (23), and utilized for further downstream analysis. The high-quality reads were then mapped against the human reference genome hg19 (human genome19) using Bowtie 2 aligner (24), and normalized as reads per kilobase per million reads (RPKM). Furthermore, the assembled RNA transcripts’ differential expression profiles were analyzed using integrated differential expression and pathway analysis (iDEP) v0.90 (25). The RPKM values were then transformed into log2 Fold Change (Log2 FC) values for each lncRNA transcript in ESCC patients compared to a healthy individual sample. Besides, identification and annotation of lncRNAs were made using NONCODE v3.0 (Please refer to the **Supplementary File S1**). The overall workflow of NGS is shown in **Supplementary Figure F1**.

### LncRNA Isolation, cDNA Synthesis, and Quantitative Real-Time PCR (qRT-PCR)

We performed expression analysis of lncRNAs in esophageal samples using the TCGA database as a training set to verify the expression levels of top five upregulated and top five downregulated lncRNAs obtained in the NGS data. Further, we performed qRT-PCR in 50 ESCC blood samples and age-sex matched healthy individuals as control (including samples used in NGS). For this, lncRNAs were isolated from 3 mL blood samples of ESCC patients and age-sex matched healthy controls using the QIamp Blood mini kit (Cat. No. 330404, Qiagen, Inc., Valencia, CA, USA) as described previously (26). We measured RNA concentration and quality using Nano"_xm_rop UV-Vis Spectrophotometer 2000cc (Thermo Fisher Scientific, CA, USA) followed by cDNA synthesis described previously (26). The qRT-PCR reactions were carried out as described previously (26). We normalized the expression of these lncRNAs using Glyceraldehyde-3-phosphate dehydrogenase (*GAPDH)* as the reference gene and calculated the fold change with the 2^-ΔΔCT^ method. We plotted normalized expression levels (ΔCt) of lncRNAs in the form of scatter plots.

### Diagnostic and Prognostic Value of Validated lncRNA(s) in ESCC Patients

Based on the normalized Ct value (ΔCt), ROC and Kaplan–Meier survival curves were plotted to evaluate the diagnostic and prognostic potential of *LINC00324* and *LOC100507053* for ESCC patients, respectively. For this, ESCC patients (n = 50) were classified as “high expression group” (n = 25) and “low expression group” (n = 25) or “high-risk” and “low-risk” group based on the median ΔCt value of *LINC00324* (5.41) and median ΔCt value of *LOC100507053* (9.56). Furthermore, patients were followed up for 15 months by either telephone or in-person from the hospital outpatient department. We used Kaplan–Meier method and log-rank test to compare the differences in overall survival time between the high-risk” and “low-risk” groups. We used “1” as the death event, while “0” was used for the censored cases.

### LncRNA Target Prediction

It is well established that lncRNA acts as a competitive endogenous RNA (ceRNA) by harboring multiple miRNA binding sites, thereby regulating their downstream targets by “sponging” those miRNAs (7). Therefore, we were interested to understand the miRNA-mRNA target of *LINC00324* and *LOC100507053.* We utilized lncRnome (http://genome.igib.res.in/lncRNome/) (27), RAID v2.0 (RNA Interactome) (http://www.rna-society.org/raid/), (28) Starbase (http://starbase.sysu.edu.cn/) (29), miRDB (microRNA database) (http://mirdb.org/) (30), TargetScan (http://www.targetscan.org/vert_72/) database (31) for predicting the targets (Please refer to the **Supplementary File S1**).

### Gene Ontology (GO) and KEGG Pathway Analysis

We performed GO and KEGG pathway analysis to determine the role of *LINC00324*, *LOC100507053*, and their targets in ESCC pathogenesis. The predicted targets were uploaded on the Starbase database for annotation, and functional analysis, including gene set enrichment analysis and mapping gene sets to the KEGG pathway. We plotted the top 15 processes and the pathways based on the –log2(FDR) value (Please refer to the **Supplementary File S1** **).**

### Co-Expression Network of Validated lncRNAs

To examine the potential functions of validated differentially expressed lncRNAs, we performed co-expression gene analysis using a co-express database (https://coxpresdb.jp/) (32) and constructed a network for co-expressed genes using Cytoscape (33). The co-expression network was constructed by calculating the correlation value’s rank, which partially normalizes genes’ density in correlation space. Further, we evaluated the associated KEGG pathways and the molecular location of co-expressed genes. For co-expression gene analysis, the correlation coefficient was set to 0.85 and *p-values* < 0.05.

### Statistical Analysis

GraphPad Prism v8.0 (La Jolla, California, USA) was used for all data analysis. Differences in the expression levels between lncRNAs and healthy individuals (control) were evaluated using the Mann-Whitney test (non-parametric test) and represented the data as mean ± standard error mean. The relationship between lncRNAs’ expression and clinicopathological characteristics were evaluated using the Chi-square test. For statistical significance, **** represent *p-value* < 0.0001, *** represent *p-value* < 0.001, ** represent *p-value* < 0.01 and * represent *p-value* < 0.05 while ns represent non-significant differences.

## Results

### LncRNAs Differentially Expressed in ESCC and Possess the Diagnostic Potential

**Figure 1:**
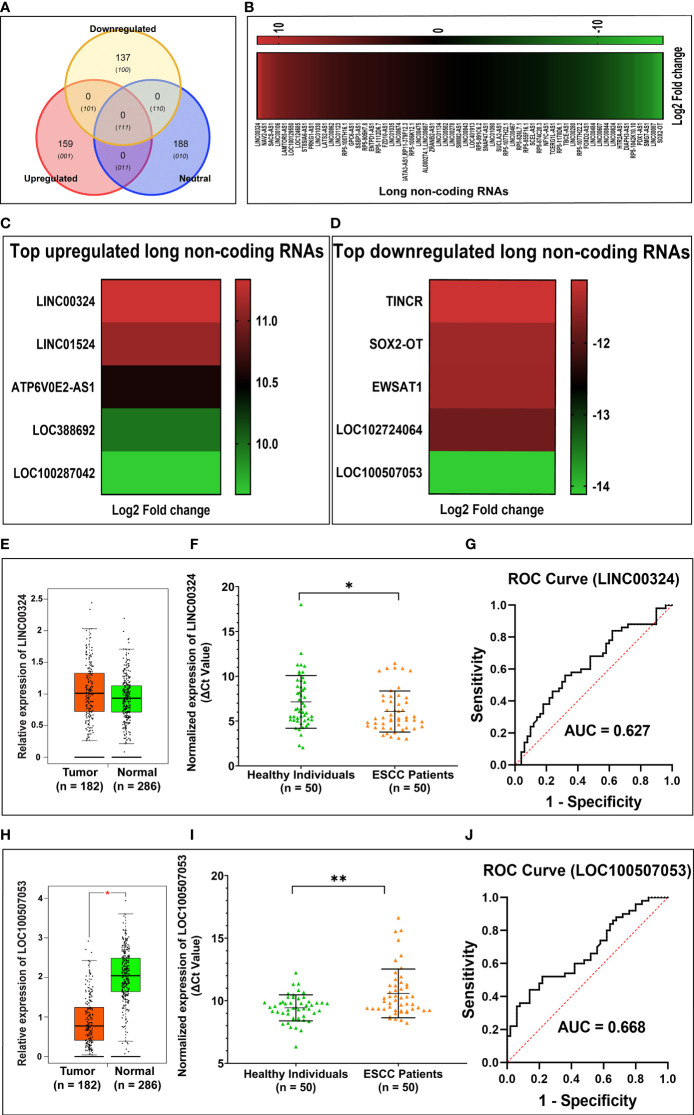
Summary of differentially expressed lncRNAs identified in esophageal squamous cell carcinoma (ESCC) patients’ blood samples. **(A)** Venn diagram showing the distribution of differentially expressed lncRNAs in ESCC. **(B)** Heatmap showing the list of differentially expressed lncRNAs according to their Log2 Fold change (*p-value* < 0.05). **(C)** Top five upregulated lncRNAs in ESCC. **(D)** Top five downregulated lncRNAs in ESCC. **(E)** Relative expression of *LINC00324* using TCGA database **(F)** Normalized expression of *LINC00324* (ΔCt values). **(G)** ROC curve to demonstrate the diagnostic potential of *LINC00324*. **(H)** Relative expression of *LINC00324* using TCGA database. **(I)** Normalized expression of *LOC100507053* (ΔCt values). **(J)** ROC curve to demonstrate the diagnostic potential of *LOC100507053*. Experiments were performed in triplicates for at least three independent times. Data are presented as the mean ± SEM. *represents *p*<0.05, **represents *p*<0.01 and calculated using a Mann Whitney test. ΔCt value was used to show relative expression of *LINC00324* and *LOC100507053* using ΔCt = Ct(Mean Ct value of LncRNA target - Mean Ct value of *GAPDH*). Small ΔCt value indicates higher expression while large ΔCt value indicates lower expression.

Through Illumina RNA sequencing technology, we generated ~50 million reads. After bioinformatics analysis, we found 484 lncRNAs transcripts. Among them, we observed 296 lncRNAs were differentially expressed in ESCC patients (n = 4) compared to healthy control (n = 4) blood samples (**Supplementary Table S1** and **Figures 1A, B**). Out of these, 159 lncRNAs were found to be upregulated (log2 FC ≥ 2 and *p-value* < 0.05), 137 lncRNAs were downregulated (log2 FC ≤ 2 and *p-values* < 0.05). The remaining 188 lncRNAs were neutrally expressed (-2<log2 FC<2 and *p-value* > 0.05) in ESCC patients’ samples (**Table 2** and **Figures 1A, B**). The volcano plot and hierarchical cluster depicting the expression of each lncRNAs shown in **Supplementary Figure F2**. To further support the above discovery, we explored the expression of the top five upregulated and downregulated lncRNAs in esophageal cancer samples using the TCGA database as the training set. Later, we validated the expression level of the top five upregulated (*LINC00324, LINC01524, ATP6V0E2-AS1, LOC388692*, and *LOC100287042*) (**Table 3** and **Figure 1C**) and top five downregulated (*TINCR, SOX2-OT, EWSAT1, LOC102724064*, and *LOC100507053*) (**Table 4** and **Figure 1D**) lncRNAs in (n = 50) samples by qRT-PCR. The results showed that *LINC00324 was* significantly upregulated (fold-change = 2.11, *p-value =* 0.0032) (**Figure 1F**), while *LOC100507053 was* downregulated (fold-change = 2.22; *p-value =* 0.0001) (**Figure 1I**) in ESCC blood samples compared to matched healthy individuals and in line with the TCGA dataset (**Figures 1E, H**). Next, we found that *LINC00324* and *LOC100507053* possess the clinical diagnostic potential with Area under the ROC curve (AUC) value was 0.627 [95% CI = 0.5172 to 0.7368; *p-value* = 0.0286] (**Figure 1G**) and 0.668 [95% CI = 0.5629 to 0.7735; *p-value* = 0.0037] respectively (**Figure 1J**). The criteria for selection of *LINC00324* and *LOC100507053* were: **1.** LncRNAs should be expressed in ≥ 80% of the samples and their levels should be matching with the TCGA data. **2.** Consistency of lncRNAs’ expression in all samples. **3.** lncRNAs whose fold change is ≥ +2 or ≤ -2. **4.** Highly sensitive and specific lncRNAs, which can significantly discriminate the ESCC patients from healthy individuals. **5.** A better AUC curve value (≥ 0.600) among the top five upregulated and top five downregulated lncRNAs. Overall, these data suggest the efficacy of lncRNAs *LINC00324* and *LOC100507053* in the diagnosis of ESCC.

**Table 2:** Differential gene expression of lncRNAs in ESCC vs. Control samples.

	No. of Upregulated lncRNAs	No. of Downregulated lncRNAs	No. of Neutrally Regulated lncRNAs
ESCC vs. Control sample	159	137	188
Conditions	Log2Fold change ≥ +2	Log2Fold change ≤ -2	-2<Log2Fold change <+2

**Table 3:** Characteristics of top 5 upregulated lncRNAs.

S.No	LncRNA ID	Accession No.	Ensemble ID	Locus	Log2 Fold Change (*p-value*<0.05)	LncRNAs Reported on Cancers
1	LINC00324	NR_026951	ENSG00000178977	17:8,123,948-8,127,361	~ 11.33	Gastric cancer, colorectal cancer, acute myeloid leukemia, osteosarcoma, lung adenocarcinoma, liver cancer, breast cancer, retinoblastoma
2	LINC01524	NR_110038	ENSG00000234948	20:50,827,184-51,266,970	~ 11.11	Gastric cancer
3	ATP6V0E2-AS1	NR_027040	ENSG00000204934	7:149,564,783-149,577,699	~ 10.56	Not reported in any cancer
4	LOC388692	NR_027002	ENSG00000263590	-	~ 9.97	Not reported in any cancer
5	LOC100287042	NR_036520	-	17:73,267,380-73,269,976	~ 9.59	Not reported in any cancer

**Table 4:** Characteristics of top 5 downregulated lncRNAs.

S.No	LncRNA ID	Accession No.	Ensemble ID	Locus	Log2 Fold Change (*p-value*<0.05)	LncRNAs Reported on Cancers
1	TINCR	NR_027064.3	ENSG00000223573	19:5,558,178-5,568,045	~ -11.12	Gastric cancer, colon cancer, breast cancer, lung cancer, esophageal cancer, bladder cancer, prostate cancer, hepatocellular carcinoma
2	SOX2-OT	NR_075093.1	ENSG00000242808	3:180,707,558-181,554,668	~ -11.47	Prostate cancer, Osteosarcoma, Cholangiocarcinoma, laryngeal cancer, Lung squamous cell carcinoma, Esophageal squamous cell carcinoma
3	EWSAT1	NR_026949	ENSG00000212766	15:69,365,266-69,388,164	~ -11.49	Colorectal cancer, Ovarian cancer, Osteosarcoma, Nasopharyngeal carcinoma, Ewing sarcoma
4	LOC102724064	XR_428946	-	-	~ -11.80	Not reported in any cancer
5	LOC100507053	NR_037884	-	-	~ -14.11	Not reported in any cancer

### *LINC00324* and *LOC100507053* Expression Correlates With Lifestyle Status of ESCC Patients Compared to Healthy Individuals

**Figure 2:**
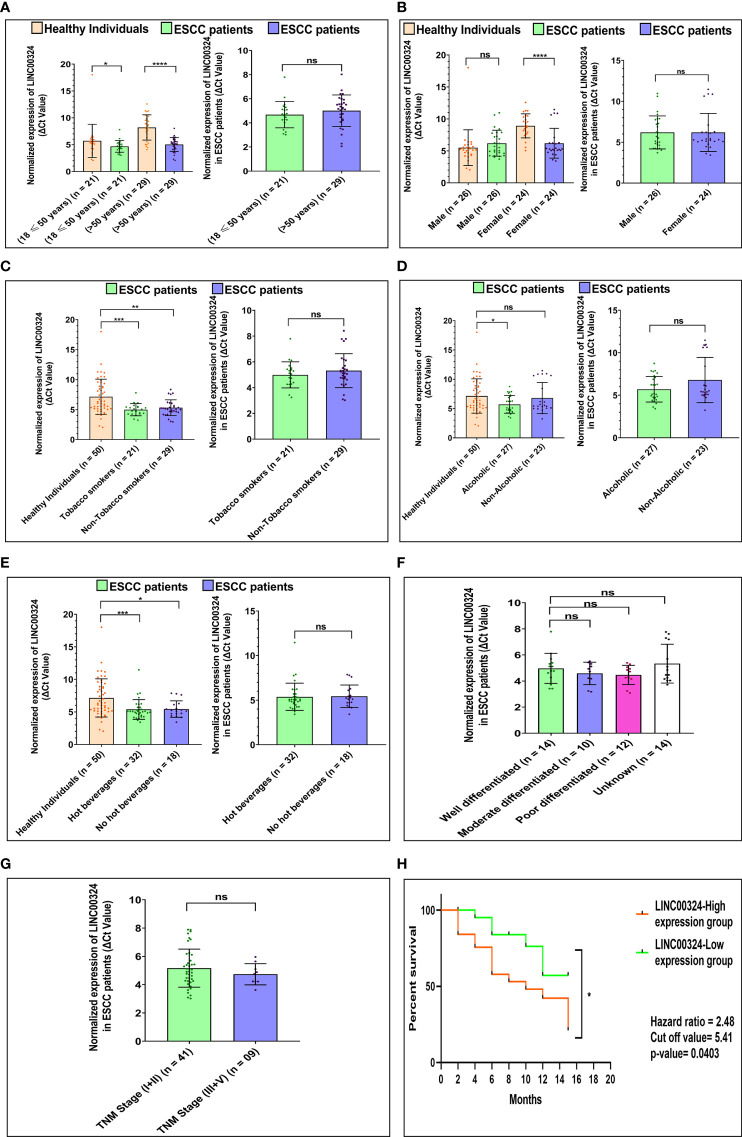
Association of *LINC00324* expression with ESCC patients’ lifestyle status and clinicopathological characteristics compared to healthy individuals. **(A)** Age of ESCC patient. **(B)** Gender of ESCC patients. **(C)** Tobacco smoking status of ESCC patients. **(D)** Alcoholic status of ESCC patients. **(E)** Consumption of hot beverages status. **(F)** Histopathological grading of the ESCC patients. **(G)** TNM staging of the ESCC patients. A Scatter plot with Bar graphs represents the normalized expression of *LINC00324* in ESCC patients compared to healthy individuals. The data are expressed as mean ± SEM where *represents *p-value* < 0.05, **represents *p-value* < 0.01, ***represents *p-value* < 0.001, and ****represents *p-value* < 0.0001 calculated using unpaired (Mann-Whitney test), paired *t-tests* and Chi-square test. **(H)** Kaplan-Meier survival plot represents the relationship between the levels of *LINC00324* expression and survival percentage of ESCC patients [TNM: tumor node metastasis].

Next, we deciphered the association of altered levels of *LINC00324* and *LOC100507053* with the clinicopathological characteristics and lifestyle factors such as age, gender, tobacco smoking, alcohol consumption, hot beverages, tumor grade, and TNM stages (**Table 1**) of the ESCC patients. For this, based on the median expression level of both lncRNAs, samples were grouped into two: high expression group and low expression group (**Table 1**). Notably, we observed an ~2.02-fold upregulation of *LINC00324* in ESCC patients having age 18 ≤ 50 years (n = 21; *p-value* = 0.03), while ~9.08-fold upregulation in ESCC patients with age greater than 50 years (n = 29; *p-value* = <0.0001) compared to respective age-matched healthy individuals (**Figure 2A**). However, no significant differences in the expression of *LINC00324* were observed between the two age groups of ESCC patients (*p-value* = 0.1459) (**Table 1** and **Figure 2A**). Similarly, we could not observe the significant difference in *LINC00324* expression between the two groups of ESCC patients’ gender (*p-value* = >0.9999). However, female ESCC patients showed an ~7.40-fold upregulation (n = 24; *p-value <*0.0001) of *LINC00324* expression compared to female healthy individuals, whereas no significant association of *LINC00324* expression was observed in male ESCC patients compared to male healthy individuals (n = 26; *p-value* = 0.2003) (**Table 1** and **Figure 2B** **).**

Further, ESCC patients with a history of tobacco smoking showed an ~4.45-fold upregulation (n = 21; *p-value* = 0.0005), and non-smokers showed an ~3.55-fold upregulation (n = 29; *p-value* = 0.0015) of *LINC00324* compared to healthy individuals (**Figure 2C**). We could not find any significant differences among tumorous smokers and non-smokers **(** *p-value* = 0.9634) (**Table 1** and **Figure 2C**). Similarly, ESCC patients with a history of alcohol consumption showed an ~2.72-fold upregulation (n = 27; *p-value* = 0.0160), while non-alcoholic patients did not show any significant differences compared to the healthy individuals (*p-value* = 0.5416) (**Figure 2D**) and between the two groups of ESCC patients **(** *p-value* = 0.9819) (**Table 1** and **Figure 2D**). Additionally, patients with a history of consumption of hot beverages showed an ~3.55-fold upregulation (n = 32; *p-value* = 0.0007), while patients with no history of consumption of hot beverages showed an ~3.53-fold upregulation (n = 18; *p-value* = 0.0111) compared to the healthy individuals (**Figure 2E**). No significant difference observed between the two groups of ESCC patients (*p-value =* 0.8446) (**Table 1** and **Figure 2E**).

When we evaluated the expression of *LINC00324* with *various* tumor grades of the ESCC patients, no significant differences were observed among the groups of ESCC patients. (**Table 1** and **Figure 2F**). Similarly, ESCC patients with the TNM stage (I+II) and (III+IV) could not show significant differences between the two groups of ESCC patients (*p-value* = 0.8136) (**Table 1** and **Figure 2G**).

**Figure 3:**
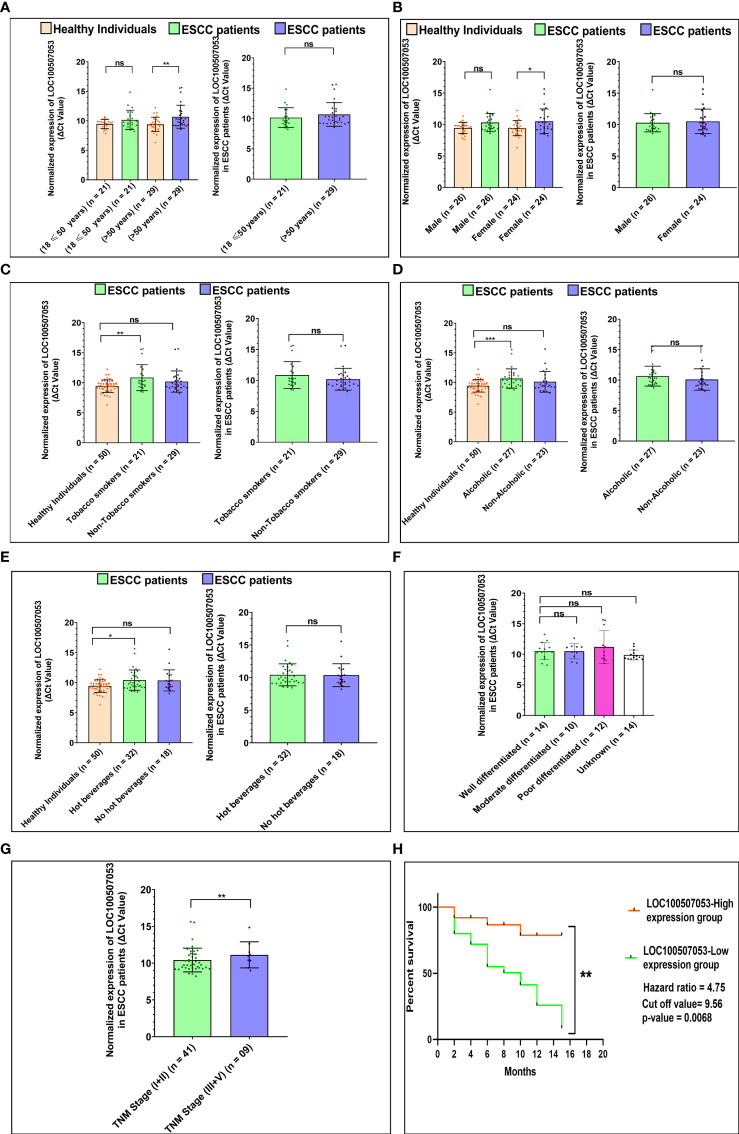
Association of *LOC100507053* expression with ESCC patients’ lifestyle status and clinicopathological characteristics compared to healthy individuals. **(A)** Age of ESCC patient. **(B)** Gender of ESCC patients. **(C)** Tobacco smoking status of ESCC patients. **(D)** Alcoholic status of ESCC patients. **(E)** Consumption of hot beverages status. **(F)**. Histopathological grading of the ESCC patients. **(G)** TNM staging of the ESCC patients. A scattered plot with Bar graphs represents the normalized expression of *LOC100507053* in ESCC patients compared to healthy individuals. The data are expressed as mean ± SEM where *represents *p-value* < 0.05, **represents *p-value* < 0.01, ***represents *p-value* < 0.001, and ****represents *p-value* < 0.0001 calculated using unpaired (Mann-Whitney test), paired *t-tests* and Chi-square test. **(H)** Kaplan-Meier survival plot represents the relationship between the levels of *LOC100507053* expression and survival percentage of ESCC patients.

Similar to the above section, for *LOC100507053*, we observed an ~1.62-fold downregulation in ESCC patients with age 18 ≤ 50 years (n = 21; *p-value* = 0.3395), and an ~2.42 -fold downregulation in ESCC patients with age greater than 50 years (n = 29; *p-value* = 0.0072) compared to respective age-matched healthy individuals (**Figure 3A**). In contrast, no significant differences in *LOC100507053* levels observed between the age groups of ESCC patients. (**Table 1** and **Figure 3A**). Moreover, we observed an ~1.82-fold downregulation in male ESCC patients (n = 26; *p-value* = 0.0683) and an ~2.11-fold downregulation in female ESCC patients (n = 24; *p-value* = 0.0489) compared to respective healthy individuals (**Figure 3B**). However, no significant difference in *LOC100507053* expression observed between the two groups of ESCC patients (**Table 1** and **Figure 3B** **).**

Furthermore, ESCC patients with a history of tobacco smoking showed an ~2.69-fold downregulation (n = 21; *p-value* = 0.0058) and non-smokers showed an ~1.69-fold downregulation (n = 29; *p-value* = 0.1045) of *LOC100507053* compared to healthy individuals (**Figure 3C**). No significant difference was observed between the two groups of ESCC patients (**Table 1** and **Figure 3C**). Similarly, we observed an ~2.34-fold downregulation (n = 27; *p-value* = 0.0005) in ESCC patients with a history of alcohol consumption, while non-alcoholic patients showed an ~1.60-fold downregulation (n = 23; *p-value* = 0.3790) compared to the healthy individuals (**Figure 3D**). However, no significant association was observed between the two groups of ESCC patients (**Table 1** and **Figure 3D**). Additionally, patients with a history of consumption of hot beverages showed an ~1.99-fold downregulation (n = 32; *p-value* = 0.0118), while patients with no history of consumption of hot beverages showed an ~1.91-fold downregulation (n = 18; *p-value* = 0.0652) compared to the healthy individuals (**Figure 3E**). No significant difference was observed between the two groups of ESCC patients (**Table 1** and **Figure 3E**).

In addition, we could not observe a significant association of *LOC100507053* expression among the various tumor grades of the ESCC patients (**Table 1** and **Figure 3F**). Similarly, we could not observe a significant association of *LOC100507053* expression with the TNM stage (I+II) and TNM stage (III+IV) group (**Table 1** and **Figure 3G**). From the above data, we can conclude that the expression of *LINC00324* and *LOC100507053* were affected by the ESCC patients’ lifestyle status compared to healthy individuals. In contrast, the clinicopathological characteristics of the ESCC patients could not affect the expression of *LINC00324* and *LOC100507053*.

### Expression of lncRNA *LINC00324* and *LOC100507053* Correlates With Poor Prognosis in ESCC Patients

To know the clinical relevance of *LINC00324 and LOC100507053* as a prognostic marker, ESCC patients were classified into high and low expression groups based on the median ΔCt value of 5.41 for *LINC00324 and* 9.56 *for LOC100507053*. Using Kaplan-Meier and log-rank analyses, we determined the survival of patients for 15 months based on the high and low expressing ESCC patient groups. The results revealed that patients in the low *LINC00324* expression group had a significantly better survival percentage than the high *LINC00324* expression group (*p-value* = 0.0279; 95% CI: 1.055 to 5.835) (**Figure 2H**). Further, ESCC patients in the high *LOC100507053* expression group had a significantly better survival percentage compared to the low *LOC100507053* expression group (*p-value* = 0.0068; 95% CI: 2.098 to 10.76) (**Figure 3H**). The hazard ratio in patients with high *LINC00324* expression was 2.48 (95% CI: 1.055 to 5.835), representing a higher risk when *LINC00324* was higher. Similarly, the hazard ratio in patients with low *LOC100507053* expression was 4.75 (95% CI: 2.098 to 10.76), representing a higher risk when *LOC100507053* was lower. Hence, the above result suggests that *LINC00324* and *LOC100507053* expression levels significantly correlated with the poor prognosis and can be an independent prognostic factor for the overall survival of ESCC patients.

### *LINC00324* Targets miRNAs and mRNAs and Modulates Various Pathways

**Figure 4:**
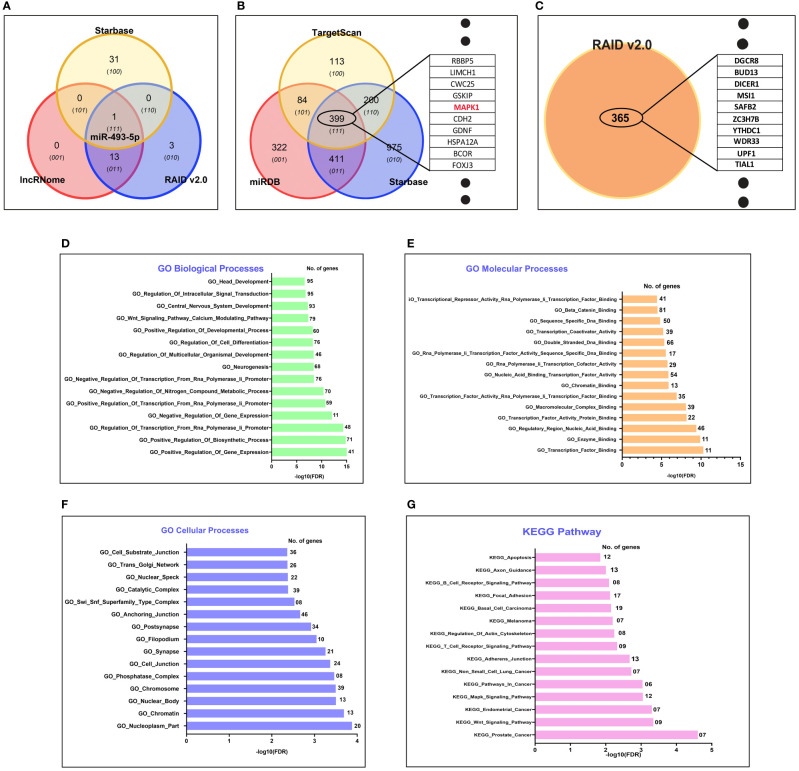
*In silico* target prediction of *LINC00324* and *LOC100507053* using online databases. **(A)** hsa-miR-493-5p predicted as miRNA target of *LINC00324* from Starbase, lncRnome, and RAID v2.0 databases. **(B)** 399 mRNA targets of hsa-miR-493-5p predicted from Starbase, miRdb, and TargetScan databases. **(C)** 365 mRNA targets of *LOC100507053* predicted from the RAID v2.0 database. **(D–G)** Gene ontology (GO) biological, cellular, biological processes and KEGG pathway of predicted target has-miR-493-5p of *LINC00324.*.

To explore the downstream molecular targets of *LINC00324*, we utilized various online databases (see *Materials and Methods*). Interestingly, we observed 14 miRNAs targets from the lncRnome database, 17 miRNAs targets from the RAID v2.0 database, and 32 miRNAs targets from the Starbase database (**Supplementary Table S2**). The common hsa-miR-493-5p was observed to be the direct target of *LINC00324* (**Figure 4A**). We analyzed the mRNA target of hsa-miR-493-5p to assess the pathway axis for its regulation during ESCC development. We observed 1216 mRNAs target from the miRdb database, 1986 mRNAs target from the Starbase database, and 796 mRNAs target from the TargetScan database (**Supplementary Table S3**). 399 mRNA targets were common to miRdb, Starbase, and TargetScan databases (**Figure 4B**).

Interestingly, we used GO analysis to understand the associated biological, molecular, and cellular processes of the hsa-miR-493-5p-*LINC00324* axis. We selected the top 15 enriched GO terms in each domain. Among them, we observed hsa-miR-493-5p was mainly involved in various biological processes, including head development, regulation of intracellular signal transduction, Wnt signaling pathway, regulation of cell differentiation (**Figure 4D**). Likewise, hsa-miR-493-5p facilitates beta-catenin binding, transcription factor binding, double-stranded DNA binding (**Figure 4E**) and was also involved in cellular processes such as anchoring junction, nucleoplasm, chromatin, cell junction (**Figure 4F**). Moreover, the pathway analysis showed that hsa-miR-493-5p enriched in the MAPK pathway, apoptosis pathway, endometrial cancer pathway, adhesion junction pathways in cancer (**Figure 4G**). Since hsa-miR-493-5p is enriched in the MAPK pathway, we are curious to understand the involvement of the MAPK signaling pathway in association with *LINC00324* expression. In the future, we will validate the molecular pathways using various molecular techniques in the wet lab.

Overall, the above data suggest that *LINC00324* may promote tumorigenesis by targeting hsa-miR-493-5p, affecting the MAPK signaling pathway and thus alters the vital biological pathways’ mechanism.

### *LOC100507053* May Regulate Through Targeting 365 mRNAs

We predicted 365 mRNAs targets from the RAID v2.0 database (**Supplementary Table S4** and **Figure 4C**). Moreover, we could not find the miRNAs targets from online databases because it has not been studied in detail yet, leaving only the information of target mRNAs. These predicted targets need experimental verification. Unfortunately, we could not predict the biological, molecular, cellular processes and KEGG pathways for *LOC100507053*. Overall, the above data suggested that *LOC100507053* might promote ESCC pathogenesis by regulating the various predicted mRNA molecules.

### *LINC00324* and *LOC100507053* Were Regulated by Co-Expression Networks of mRNAs and lncRNAs

**Figure 5:**
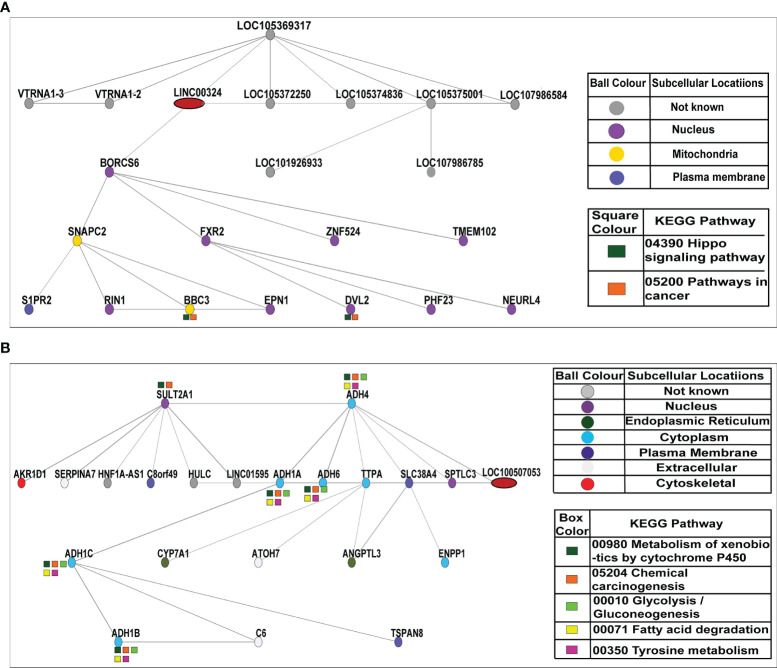
Co-expression network of *LINC00324* and *LOC100507053*. **(A)** A total of nine lncRNAs and twelve mRNAs interacted with *LINC00324*. **(B)** A total of three lncRNAs and eighteen mRNAs interacted with *LOC100507053*.

We constructed co-expression networks to identify co-expressed genes with lncRNA, potentially acting as potential target genes for lncRNA. With this idea, we drew the network of two lncRNAs (*LINC00324* and *LOC100507053*) using the Cytoscape and Adobe Illustrator CC 2015.3 program. The network indicated that each lncRNA correlated with other lncRNAs and mRNAs. Notably, nine lncRNAs *LOC105369317, Vault RNA 1-3* (*VTRNA1-3*)*, Vault RNA 1-2* (*VTRNA1.2*)*, LOC105372250, LOC105374836*, *LOC105375001, LOC107986584, LOC101926933, and LOC107986785*) and twelve mRNAs, BLOC-1 related complex subunit 6 (*BORCS6*), fragile X-mental retardation 1-Like 2 (*FXR2*), zinc finger protein 524 (*ZNF524*), transmembrane protein 102 (*TMEM102*), Ras And Rab interactor 1 (*RIN1*), epsin 1 (*EPN1*), dishevelled segment polarity protein 2 (*DVL2*), PHD finger protein 23 (*PHF23*), neutralized E3 ubiquitin protein ligase 4 (*NEURl4*), nematode anticoagulant protein c2 (*NAPC2*), sphingosine-1-phosphate receptor 2 (*S1PR2*), and BCL2 binding component 3 (*BBC3*) were predicted to be co-expressed with *LINC00324*. Among them, *LOC105375001*, *BORCS6*, *LOC105369317*, *LOC105372250* directly co-expressed with *LINC00324* on the network. Besides, ten mRNAs (*BORCS6*, *FXR2*, *ZNF524*, *TMEM102*, *RIN1*, *EPN1*, *DVL2*, *PHF23*, and *NEURl4*) were localized in the nucleus (as shown in purple ball), two mRNAs (*SNAPC2* and *BBC3*) were localized in the mitochondria (as shown in yellow ball), and one mRNA (*S1PR2*) was localized in the plasma membrane (as shown in the dark blue ball). Furthermore, *BBC3* and *DVL2* were involved in Hippo signaling pathways (shown in dark green color box) and cancer pathways (shown in orange color box) (**Figure 5A**).

Likewise, three lncRNAs HNF1A antisense RNA 1 (HNF1A-AS1), hepatocellular carcinoma up-regulated long non-coding RNA (HULC), and long intergenic non-protein coding RNA 1595 (LINC01595) and eighteen mRNAs sulfotransferase family 2A member 1 (SULT2A1), alcohol dehydrogenase 4 (ADH4), aldo-keto reductase family 1 member D1 (AKR1D1), serpin family A member 7 (SERPINA7), chromosome 8 putative open reading frame 49 (C8orf49), alcohol dehydrogenase 1A (ADH1A), alcohol dehydrogenase 6 (ADH6), alpha tocopherol transfer protein (TTPA), solute carrier family 38 member 4 (SLC38A4), serine palmitoyltransferase long chain base subunit 3 (SPTLC3), alcohol dehydrogenase 1C (ADH1C), cytochrome P450 family 7 subfamily A member 1 (CYP7A1), atonal BHLH transcription factor 7 (ATOH7), angiopoietin like 3 (ANGPTL3), ectonucleotide pyrophosphatase/phosphodiesterase 1 (ENPP1), alcohol dehydrogenase 1B (ADH1B), complement C6 (C6), tetraspanin 8 (TSPAN8) were predicted to be co-expressed with LOC100507053. Among them, ADH6, ADH1A, and ADH4 were directly co-expressed with LOC100507053 on the network. Besides, two mRNAs (SULT2A1 and SPTLC3) were localized in the nucleus (as shown in purple ball), 2 mRNAs (CYP7A1 and ANGPTL3) were localized in the endoplasmic reticulum (as shown in dark green ball), 7 mRNAs (ADH4, ADH1A, ADH6, TTPA, ADH1C, ENPP1, ADH1B) were localized in the cytoplasm (as shown in blue ball), 3 mRNAs (SERPINA7, ATOH7, and C6) were localized in the extracellular spaces (as shown in pale white ball), and one mRNA (AKR1D1) was localized in the cytoskeletal (as shown in red ball). As it was reported that LOC100507053 is a metabolic pathway associated-lncRNA (30320481), we observed 6 co-expressed mRNAs (SULT2A1, ADH4, ADH1A, ADH6, ADH1C, ADH1B) were involved in the metabolism of xenobiotic by P450 (shown in dark green color box) and in the chemical carcinogenesis pathways (shown in orange color box). Moreover, five mRNAs (ADH4, ADH1A, ADH6, ADH1C, and ADH1B) were involved in the glycolysis/gluconeogenesis pathways (shown in light green color box), fatty acid degradation pathways (shown in yellow color box), and tyrosine metabolism pathways (shown in pink color box) (**Figure 5B**).

Overall these data suggest that dysregulation of lncRNAs *LINC00324* and *LOC100507053* in ESCC simultaneously affects the expression of various other lncRNAs and mRNAs that are involved in the pivotal signaling pathways in the human biological system. This approach may help develop the possible treatment strategy by controlling the affected gene expression in combination with *LINC00324* and *LOC100507053*.

## Discussion

ESCC is a highly invasive tumor of the digestive system worldwide, with a high incidence in certain parts of India like Punjab (the studied region). At present, the detection and prognosis of ESCC depend mainly on some non-specific markers, invasive endoscopy, and painful histopathological biopsy (34); therefore, there is an unmet need for a blood-based circulating biomarker to detect and manage ESCC better.

Notably, with the advancement of high throughput next-generation sequencing methods, many lncRNAs have been explored to be used for cancer management (7, 8, 10–12, 26). However, we could not find any studies demonstrating the comprehensive investigation of lncRNAs as diagnostic/prognostic biomarkers using blood samples of ESCC patients. Hence, with the above idea, we planned a pilot study to discover the potential diagnostic and prognostic blood-based biomarker for ESCC. We utilized next-generation sequencing in the discovery set of samples to find the dysregulated lncRNAs in ESCC blood samples. We found 296 differentially expressed lncRNAs in ESCC blood samples compared to matched healthy individuals (**Figure 1**). Further, we utilized the TCGA samples as a training set to verify the expression of the top five upregulated and downregulated lncRNAs. Additionally, Using qRT-PCR validation study in n = 100 samples, we observed *LINC00324* and *LOC100507053* lncRNAs were significantly dysregulated and in line with our NGS and TCGA data (**Figure 1**). Our finding contrasts with an earlier study where Khadirnaikar etal. (35), through TCGA data retrieval, showed 127 differentially expressed lncRNAs in EC patients samples compared to normal individuals (35). However, they have taken mixed sample types like ESCC and AC patients’ tissues and blood. Similarly, Li etal. (36) demonstrated 127 differentially expressed lncRNAs in ESCC tissue samples compared to control tissue samples. Further, through qRT-PCR, four significantly dysregulated lncRNAs, *RP11-334E6.12* and *RP11-150O12.6*, *AC103563.9*, and *RP11-7 K24.3* found in ESSC tissue samples (36). Moreover, Li et al. have used cutoff fold-change >4, whereas, here, in this study, we have taken cutoff fold-change ≥ 2. Prominently, these differentially expressed lncRNAs are not the same as we have seen in our research here. The reason could be varying population profiles, genetics/epigenetics differences in patients, and samples (tissue Vs. blood).

It is well known that some extrinsic lifestyle factors also play an essential role in the etiology of ESCC, like intake of hot beverages, amount of fresh food and vegetables, obesity, HPV infection (37–39). Indeed, previous studies suggested the association of validated lncRNAs with various lifestyle factors of cancer patients (40–45). Unfortunately, their association with ESCC patients is still missing. Therefore, we were interested in deciphering the implications of *LINC00324* and *LOC100507053* expressions with lifestyle status and clinicopathological characteristics of ESCC patients as well as the diagnostic and prognostic potential of lncRNAs. Interestingly, we observed upregulated expression of *LINC00324* and downregulated expression of *LOC100507053* linked with the age (>50 years) of the ESCC patients compared to age-matched healthy individuals (**Table 1**, **Figures 2A** and **3A**), which suggests that the age above 50 years might affect the levels of *LINC00324* and *LOC100507053* expressions. However, no correlation was observed with *LINC00324* and *LOC100507053 expression* in the ESCC patients’ age group (**Table 1**, **Figures 2A** and **3A**). In line with this, previous reports were also unable to find the correlation of *LINC00324* and *LOC100507053* expression within the cancer patients’ age (40, 42, 44–46). Further, in our study, we found ESCC patients’ gender (Female) was associated with *LINC00324* and *LOC100507053 expression* compared to healthy individuals but not correlated with *LINC00324* and *LOC100507053 expression* in the ESCC patients’ gender (**Table 1**, **Figures 2B** and **3B**). Unlike previous reports (40, 42–45), we observed that *LINC00324* and *LOC100507053* expression were correlated with ESCC patients’ those who smoked tobacco, consumed alcohol, hot beverages compared to healthy individuals, suggesting that these factors may modulate the expression of *LINC00324* and *LOC100507053* in ESCC patients, but these factors were not correlated within the ESCC patients. (**Table 1**, **Figures 2C–E** and **3C–E**). Moreover, we could not find the association of progressive TNM stages and tissue grades with *LINC00324* and *LOC100507053* expression within the ESCC group; however, the previous findings suggested the association of *LINC00324* and TNM stages (40, 42–45) (**Table 1**, **Figures 2F, G** and **3F, G**). Furthermore, dysregulated expression of *LINC00324* and *LOC100507053* was linked to ESCC patients’ survival, suggesting the association of both lncRNAs with poor prognosis of ESCC patients (**Figures 2H** and **3H**). Therefore, we hypothesized that both the lncRNAs might have the diagnostic potential for ESCC screening. With this idea, in this study, the diagnostic value of *LINC00324* and *LOC100507053* in blood samples of ESCC patients was examined and observed that AUC value for *LINC00324* = 0.627 [95% CI = 0.5172 to 0.7368; *p-value* = 0.0286] (**Figure 1G**) and AUC value for *LOC100507053* = 0.668 [95% CI = 0.5629 to 0.7735; *p-value* = 0.0037] [**Figure 1H**].

As mentioned above, lncRNAs have an inherent ability to interact with various molecular cues during cancer pathogenesis. In this context, we identified hsa-miR-493-5p as the direct molecular target of *LINC00324* (**Figure 4A**) and three hundred ninety-nine mRNA targets of hsa-miR-493-5p through online bioinformatics software (**Figure 4B**). Out of three hundred ninety-nine mRNA, we selected MAPK based on the pathway analysis for validation in the *in vitro* specimen (**Figure 4B**). Through co-expression analysis, we identified nine lncRNAs and twelve mRNAs co-expressed with *LINC00324* (**Figure 5A**). Similarly, we identified 365 mRNAs targets of *LOC100507053* from the RAID v2.0 database (**Figure 4C**), and the co-expression analysis suggested three lncRNAs and twelve mRNAs were co-expressed with *LOC100507053* (**Figure 5B**). Earlier reports indicated that *LINC00324*, by sponging hsa-miR-214-3p, hsa-miR-139-5p, hsa-miR-769-5p promotes the progression of colorectal, lung, and retinoblastoma (41, 42, 47, 48); however, no study was conducted on ESCC. Further, *LINC00324* can enhance the stability of family with sequence similarity 83 member B (*FAM83B*) and WD repeat-containing protein 66 (*WDR66*) messenger RNA through binding to human antigen R (*HuR*), thereby promoting cell proliferation and migration in gastric cancer and osteosarcoma, respectively (40, 45). On the other hand, *LOC100507053* or *RP11-555H23.1*, a metabolic pathway associated-lncRNA, was downregulated in gastric cancer (46).

Interestingly, we saw the *LINC00324*-miR-493-5p-MAPK1 axis involved in MAPK signaling pathway in ESCC (**Figures 4D–G**). Moreover, through the co-expression networks of *LINC00324*, we observed a few mRNAs were involved in Hippo signaling pathways. It is well established that such dysregulation of pathway leads to many cancers, including ESCC (49–51). Mechanistically, MAPK molecules phosphorylate many transcription factors such as c-Myc, fos, and EGR1 (52), modulating cell proliferation and migration (53). Besides, Wnt signaling can activate both β-catenin–dependent and -independent signal transduction cascades. Constitutive activation of this pathway leads to the development of cancer (50).

Similarly, in the Hippo signaling pathway, upstream molecules interact with transcriptional factors, such as TEA domain transcription factor 1 (TEAD) and Runt-related transcription factor (RUNX), to regulate cell growth, migration, survival, and metabolism (51, 54, 55). Likewise, through a co-expression network, we observed *LOC100507053*-mRNA axis is enriched in glycolysis/gluconeogenesis pathways and tyrosine metabolism pathways (**Figure 5B**). Aberrant tyrosine kinase signaling alters the cellular metabolism and promotes the Warburg effect in cancer cells *via* transcriptional regulation of glycolytic enzymes (56).

Thus, our pilot study addresses the scientific gap in the early staged non-invasive biomarker for ESCC. A systematic approach was designed to discover the potential biomarker for ESCC. We observed an altered expression of circulating *LINC00324* and *LOC100507053* were able to distinguish the ESCC patients from healthy individuals. Their expression is associated with the lifestyle factors of ESCC patients compared to healthy individuals but not within the ESCC patients’ group. Moreover, online bioinformatics tools revealed the putative targets of *LINC00324* and *LOC100507053*.

## Conclusion, Future Aspects and Limitations of the Study

Overall, our pilot study revealed 296 differentially expressed lncRNAs in ESCC blood samples compared to healthy control individuals. Out of these 296 lncRNAs, *LINC00324* and *LOC100507053* were significantly up and downregulated respectively in ESCC patients compared to healthy individuals. Our study further demonstrated that lifestyle status affects the expression of *LINC00324* and *LOC100507053* in ESCC patients compared to healthy individuals. However, clinicopathological characteristics could not affect the *LINC00324* and *LOC100507053* expression in ESCC patients. Mechanistically, we observed miR-493-5p as the direct molecular target of *LINC00324* and interacted with the MAPK1 molecule of MAPK signaling pathway in ESCC pathogenesis.

Thus, our pilot study explores the comprehensive profiling of circulating lncRNAs to establish a liquid biopsy biomarker for the diagnosis/prognosis of ESCC in clinical settings. We identified *LINC00324* and *LOC100507053* as the potential candidate marker for ESCC detection. Furthermore, this study can be translated into clinical settings, which provides an alternative approach to conventional tissue biopsy using circulating lncRNAs to diagnose ESCC patients. Although our study can be translated, it possesses several limitations at the current stage, such as limited cohort size, experimental verification of the identified molecular underpinnings using wet-lab experiments, and multicentric studies are still warranted to translate our findings into clinical settings.

## Data Availability Statement

The original contributions presented in the study are included in the article/**Supplementary Material**. Further inquiries can be directed to the corresponding author.

## Ethics Statement

The studies involving human participants were reviewed and approved by the Ethics Review Board of Baba Farid University of Health Sciences, Faridkot (ERB/UCER/2019/4/17), and Institutional Ethics Committee of Central University of Punjab, Bathinda (CUPB/IEC/2018/12). The patients/participants provided their written informed consent to participate in this study.

## Author Contributions

US and AJ are the guarantors of the integrity of the entire study. US and AJ conceived the original idea and planned the experiments. US carried out the experiments and wrote the manuscript. US performed the bioinformatics analysis and prepared the figures. US and TB formatted references. MR, KS, and AR provided the clinical samples and their data. AJ, US, TB, and AK contributed to the final version of the manuscript. AJ supervised and supported the research. All authors contributed to the article and approved the submitted version.

## Funding

Department of Science and Technology of India supported this work through the Indo-Russia grant (INT/RUS/RFBR/P-311) to AJ and DST-INSPIRE fellowship (IF180680) to USA. AJ is also thankful to ICMR for providing grants (5/13/81/2013-NCD-III).

## Conflict of Interest

The authors declare that the research was conducted in the absence of any commercial or financial relationships that could be construed as a potential conflict of interest.

## Publisher’s Note

All claims expressed in this article are solely those of the authors and do not necessarily represent those of their affiliated organizations, or those of the publisher, the editors and the reviewers. Any product that may be evaluated in this article, or claim that may be made by its manufacturer, is not guaranteed or endorsed by the publisher.

## Supplementary Material

Supplementary Figure 1 Workflow of next-generation sequencing of ESCC patients and healthy individuals’ blood samples.

Supplementary Figure 2
**(A)** Volcano plot represents the expression pattern of lncRNAs in ESCC. The red dots represent the top upregulated lncRNAs, and the green dots represent top downregulated lncRNAs. **(B)** Hierarchical cluster represents the pattern of lncRNAs expression in ESCC and normal cohorts and demonstrates the expression similarity among lncRNAs in the respective cohorts.
