# Supplementary figures and images for: Circulating Long Non-Coding RNAs LINC00324 and LOC100507053 as Potential Liquid Biopsy Markers for Esophageal Squamous Cell Carcinoma: A Pilot Study
Source: Front Oncol. 2022 Feb 14;12:823953. doi: 10.3389/fonc.2022.823953 (PMC8882835; doi:10.3389/fonc.2022.823953)

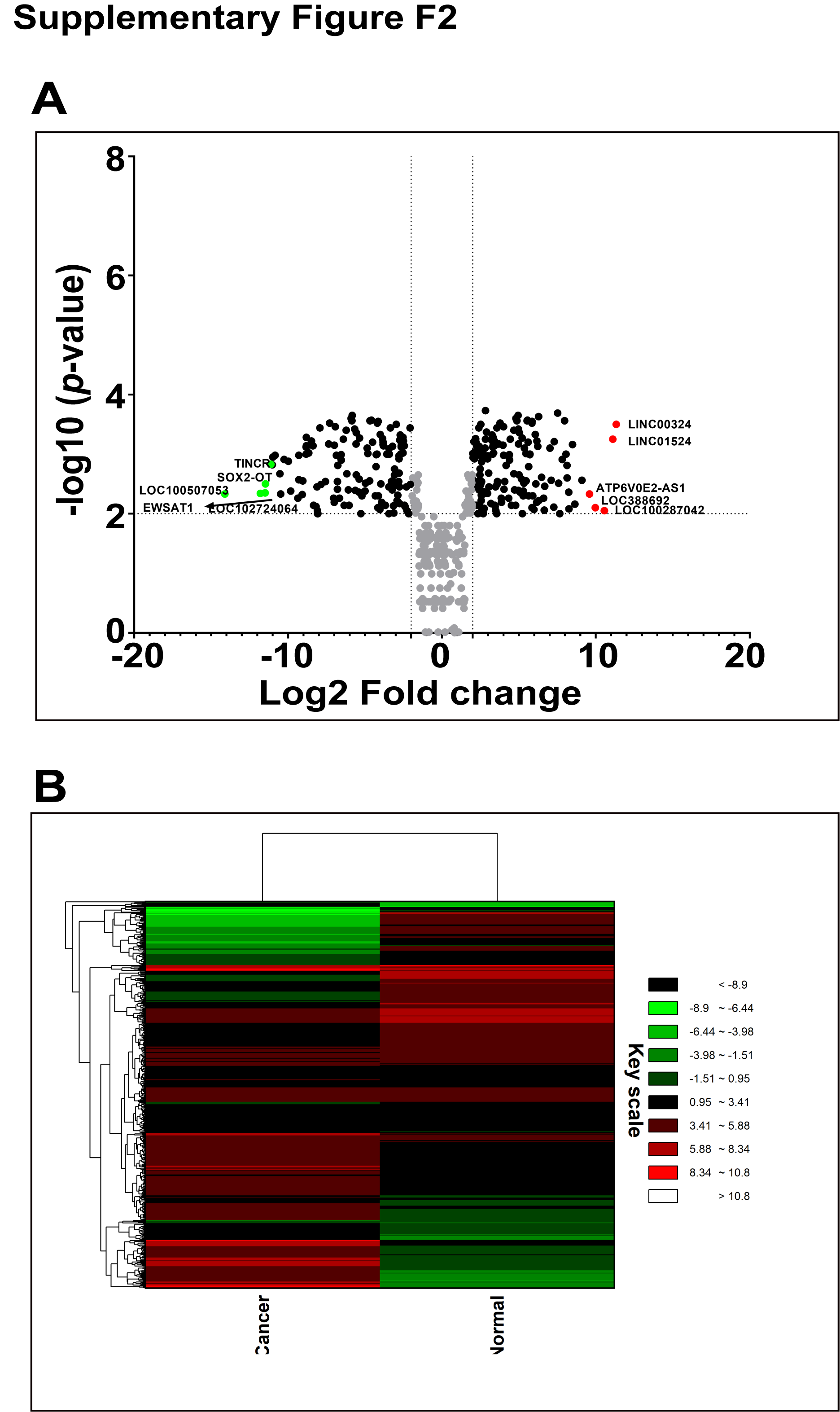

Supplement: Supplementary Figure 2 — (A) Volcano plot represents the expression pattern of lncRNAs in ESCC. The red dots represent the top upregulated lncRNAs, and the green dots represent top downregulated lncRNAs. (B) Hierarchical cluster represents the pattern of lncRNAs expression in ESCC and normal cohorts and demonstrates the expression similarity among lncRNAs in the respective cohorts. [file Image_2.tif]
