# Supplementary material for: Circulating Long Non-Coding RNAs LINC00324 and LOC100507053 as Potential Liquid Biopsy Markers for Esophageal Squamous Cell Carcinoma: A Pilot Study
Source: Front Oncol. 2022 Feb 14;12:823953. doi: 10.3389/fonc.2022.823953 (PMC8882835; doi:10.3389/fonc.2022.823953)
Supplement: Supplementary file 3 [file Table_1.docx]

**Supplementary Table 1: List of differentially expressed lncRNAs in ESCC**

| **S No.** | **Gene** | **Log2 Fold Change** | **Colour coding** | **Regulation** | **No.** |
| --- | --- | --- | --- | --- | --- |
| **1** | **LINC00324** | **11.33** |  | **Upregulated** | **159** |
| **2** | **LINC01524** | **11.11** |  | **Downregulated** | **137** |
| **3** | **ATP6V0E2-AS1** | **10.56** |  | **Neutral** | **188** |
| **4** | **LOC388692** | **9.97** |  |  |  |
| **5** | **LOC100287042** | **9.59** |  |  |  |
| **6** | **RP5-1120P11.1** | **9.10** |  |  |  |
| **7** | **RP5-884M6.1** | **8.64** |  |  |  |
| **8** | **OR5AS1** | **8.49** |  |  |  |
| **9** | **NAV2-AS1** | **8.26** |  |  |  |
| **10** | **SLC25A21-AS1** | **8.24** |  |  |  |
| **11** | **LINC00303** | **8.14** |  |  |  |
| **12** | **PROX1-AS1** | **8.13** |  |  |  |
| **13** | **RP5-1148A21.3** | **8.06** |  |  |  |
| **14** | **RP5-973N23.4** | **7.97** |  |  |  |
| **15** | **RP5-966M1.6** | **7.82** |  |  |  |
| **16** | **SORCS3-AS1** | **7.66** |  |  |  |
| **17** | **SACS-AS1** | **7.58** |  |  |  |
| **18** | **TEX36-AS1** | **7.52** |  |  |  |
| **19** | **OR2AS1P** | **7.30** |  |  |  |
| **20** | **RP5-899B16.2** | **7.27** |  |  |  |
| **21** | **DPYD-AS1** | **7.10** |  |  |  |
| **22** | **LINC01036** | **7.02** |  |  |  |
| **23** | **RP11-716O23.2** | **6.68** |  |  |  |
| **24** | **ITPKB-AS1** | **6.65** |  |  |  |
| **25** | **LINC00106** | **6.61** |  |  |  |
| **26** | **LBX1-AS1** | **6.48** |  |  |  |
| **27** | **UNC5B-AS1** | **6.30** |  |  |  |
| **28** | **LINC01032** | **6.28** |  |  |  |
| **29** | **ZNF32-AS1** | **6.23** |  |  |  |
| **30** | **LINC00094** | **6.17** |  |  |  |
| **31** | **RP5-857K21.15** | **6.16** |  |  |  |
| **32** | **LINC00595** | **6.10** |  |  |  |
| **33** | **LAMTOR5-AS1** | **6.07** |  |  |  |
| **34** | **IFNG-AS1** | **5.98** |  |  |  |
| **35** | **FAM222A-AS1** | **5.90** |  |  |  |
| **36** | **LINC00659** | **5.86** |  |  |  |
| **37** | **LINC00474** | **5.85** |  |  |  |
| **38** | **SPATA13-AS1** | **5.85** |  |  |  |
| **39** | **LINC00963** | **5.72** |  |  |  |
| **40** | **LINC00970** | **5.71** |  |  |  |
| **41** | **LOC100129550** | **5.64** |  |  |  |
| **42** | **LINC00342** | **5.64** |  |  |  |
| **43** | **RP5-905H7.5** | **5.60** |  |  |  |
| **44** | **LINC00176** | **5.56** |  |  |  |
| **45** | **LINC01136** | **5.53** |  |  |  |
| **46** | **JMJD1C-AS1** | **5.47** |  |  |  |
| **47** | **LINC00587** | **5.46** |  |  |  |
| **48** | **LINC00869** | **5.36** |  |  |  |
| **49** | **LOC124685** | **5.33** |  |  |  |
| **50** | **RNF219-AS1** | **5.29** |  |  |  |
| **51** | **LINC00968** | **5.28** |  |  |  |
| **52** | **C10orf71-AS1** | **5.23** |  |  |  |
| **53** | **LINC00633** | **5.21** |  |  |  |
| **54** | **PXN-AS1** | **5.18** |  |  |  |
| **55** | **LINC01135** | **5.08** |  |  |  |
| **56** | **TEX26-AS1** | **5.03** |  |  |  |
| **57** | **ST8SIA6-AS1** | **5.01** |  |  |  |
| **58** | **LINC00702** | **4.98** |  |  |  |
| **59** | **TRAM2-AS1** | **4.97** |  |  |  |
| **60** | **LINC00654** | **4.97** |  |  |  |
| **61** | **PROSER2-AS1** | **4.95** |  |  |  |
| **62** | **RP5-1112D6.8** | **4.87** |  |  |  |
| **63** | **NRG3-AS1** | **4.81** |  |  |  |
| **64** | **RP5-899E9.1** | **4.79** |  |  |  |
| **65** | **PRKG1-AS1** | **4.72** |  |  |  |
| **66** | **LINC00202-2** | **4.68** |  |  |  |
| **67** | **RP5-933K21.2** | **4.68** |  |  |  |
| **68** | **MALAT1** | **4.52** |  |  |  |
| **69** | **TMPO-AS1** | **4.44** |  |  |  |
| **70** | **RP5-966M1.5** | **4.39** |  |  |  |
| **71** | **RP5-966M1.4** | **4.38** |  |  |  |
| **72** | **RP5-933K21.3** | **4.36** |  |  |  |
| **73** | **LINC01030** | **4.34** |  |  |  |
| **74** | **LINC00266-2P** | **4.17** |  |  |  |
| **75** | **LINC00708** | **4.08** |  |  |  |
| **76** | **LINC00703** | **4.03** |  |  |  |
| **77** | **C1QTNF9-AS1** | **4.01** |  |  |  |
| **78** | **DYNLL1-AS1** | **4.00** |  |  |  |
| **79** | **RP5-1039K5.16** | **3.97** |  |  |  |
| **80** | **RP5-1147A1.1** | **3.87** |  |  |  |
| **81** | **LATS2-AS1** | **3.86** |  |  |  |
| **82** | **SLC39A12-AS1** | **3.78** |  |  |  |
| **83** | **LINC01155** | **3.69** |  |  |  |
| **84** | **LINC00489** | **3.61** |  |  |  |
| **85** | **KCNIP2-AS1** | **3.58** |  |  |  |
| **86** | **LINC00622** | **3.57** |  |  |  |
| **87** | **LINC01139** | **3.56** |  |  |  |
| **88** | **KCNQ1-AS1** | **3.55** |  |  |  |
| **89** | **LINC00862** | **3.50** |  |  |  |
| **90** | **LINC00266-4P** | **3.48** |  |  |  |
| **91** | **EGLN3-AS1** | **3.46** |  |  |  |
| **92** | **LINC01115** | **3.44** |  |  |  |
| **93** | **LINC00493** | **3.41** |  |  |  |
| **94** | **C6orf47-AS1** | **3.41** |  |  |  |
| **95** | **LINC00589** | **3.36** |  |  |  |
| **96** | **RP5-1157M23.2** | **3.33** |  |  |  |
| **97** | **LINC01123** | **3.32** |  |  |  |
| **98** | **KCNC4-AS1** | **3.30** |  |  |  |
| **99** | **ZMIZ1-AS1** | **3.30** |  |  |  |
| **100** | **LOC732538** | **3.26** |  |  |  |
| **101** | **RP5-1120P11.4** | **3.26** |  |  |  |
| **102** | **UBE2Q1-AS1** | **3.24** |  |  |  |
| **103** | **LINC00707** | **3.18** |  |  |  |
| **104** | **PCED1B-AS1** | **3.06** |  |  |  |
| **105** | **RP5-1007H16.1** | **3.04** |  |  |  |
| **106** | **SPATA17-AS1** | **2.97** |  |  |  |
| **107** | **USP30-AS1** | **2.97** |  |  |  |
| **108** | **OAS1** | **2.96** |  |  |  |
| **109** | **ZNF436-AS1** | **2.90** |  |  |  |
| **110** | **LINC01164** | **2.83** |  |  |  |
| **111** | **LINC00853** | **2.83** |  |  |  |
| **112** | **LINC01104** | **2.80** |  |  |  |
| **113** | **GPC6-AS1** | **2.74** |  |  |  |
| **114** | **RP5-965K10.3** | **2.74** |  |  |  |
| **115** | **LINC01114** | **2.69** |  |  |  |
| **116** | **RP5-892F13.2** | **2.68** |  |  |  |
| **117** | **MED4-AS1** | **2.68** |  |  |  |
| **118** | **LINC01132** | **2.64** |  |  |  |
| **119** | **TTLL10-AS1** | **2.63** |  |  |  |
| **120** | **LINC00705** | **2.61** |  |  |  |
| **121** | **SSBP3-AS1** | **2.61** |  |  |  |
| **122** | **LINC00583** | **2.60** |  |  |  |
| **123** | **LINC01143** | **2.58** |  |  |  |
| **124** | **DNAJC3-AS1** | **2.58** |  |  |  |
| **125** | **ADAMTSL4-AS1** | **2.56** |  |  |  |
| **126** | **LINC00211** | **2.55** |  |  |  |
| **127** | **LINC01121** | **2.53** |  |  |  |
| **128** | **RP5-1120P11.3** | **2.52** |  |  |  |
| **129** | **RP5-905H7.8** | **2.50** |  |  |  |
| **130** | **IPO9-AS1** | **2.48** |  |  |  |
| **131** | **LINC00529** | **2.47** |  |  |  |
| **132** | **FAM170B-AS1** | **2.47** |  |  |  |
| **133** | **RP5-821D11.7** | **2.45** |  |  |  |
| **134** | **CARS-AS1** | **2.42** |  |  |  |
| **135** | **LYST-AS1** | **2.41** |  |  |  |
| **136** | **MRVI1-AS1** | **2.41** |  |  |  |
| **137** | **ENTPD1-AS1** | **2.41** |  |  |  |
| **138** | **LINC00865** | **2.40** |  |  |  |
| **139** | **LINC00029** | **2.36** |  |  |  |
| **140** | **DENND5B-AS1** | **2.34** |  |  |  |
| **141** | **DAOA-AS1** | **2.34** |  |  |  |
| **142** | **LINC00926** | **2.33** |  |  |  |
| **143** | **LINC01087** | **2.30** |  |  |  |
| **144** | **CCND2-AS1** | **2.29** |  |  |  |
| **145** | **RP5-1112D6.7** | **2.28** |  |  |  |
| **146** | **RBM26-AS1** | **2.27** |  |  |  |
| **147** | **RP5-905H7.6** | **2.25** |  |  |  |
| **148** | **LINC00851** | **2.24** |  |  |  |
| **149** | **LINC00685** | **2.21** |  |  |  |
| **150** | **RRM1-AS1** | **2.19** |  |  |  |
| **151** | **SPTY2D1-AS1** | **2.18** |  |  |  |
| **152** | **LINC-PINT** | **2.15** |  |  |  |
| **153** | **FZD10-AS1** | **2.14** |  |  |  |
| **154** | **LINC01151** | **2.13** |  |  |  |
| **155** | **LINC00684** | **2.11** |  |  |  |
| **156** | **LRP4-AS1** | **2.08** |  |  |  |
| **157** | **LINC01158** | **2.03** |  |  |  |
| **158** | **ANO1-AS1** | **2.03** |  |  |  |
| **159** | **DNAJC9-AS1** | **2.01** |  |  |  |
| **160** | **LINC00184** | **1.97** |  |  |  |
| **161** | **LINC01035** | **1.96** |  |  |  |
| **162** | **ASH1L-AS1** | **1.95** |  |  |  |
| **163** | **RP5-849L7.1** | **1.93** |  |  |  |
| **164** | **LINC00843** | **1.92** |  |  |  |
| **165** | **ZNF503-AS1** | **1.91** |  |  |  |
| **166** | **RP5-919F19.5** | **1.91** |  |  |  |
| **167** | **LINC00658** | **1.90** |  |  |  |
| **168** | **BCDIN3D-AS1** | **1.86** |  |  |  |
| **169** | **LINC00674** | **1.85** |  |  |  |
| **170** | **KCTD21-AS1** | **1.84** |  |  |  |
| **171** | **RP5-1104E15.6** | **1.80** |  |  |  |
| **172** | **PCCA-AS1** | **1.76** |  |  |  |
| **173** | **FANK1-AS1** | **1.74** |  |  |  |
| **174** | **LINC01141** | **1.68** |  |  |  |
| **175** | **UBAC2-AS1** | **1.66** |  |  |  |
| **176** | **LINC00619** | **1.66** |  |  |  |
| **177** | **RP11-379F12.3** | **1.61** |  |  |  |
| **178** | **LINC00115** | **1.57** |  |  |  |
| **179** | **TNKS2-AS1** | **1.57** |  |  |  |
| **180** | **RP5-1049N15.2** | **1.54** |  |  |  |
| **181** | **C1QTNF9B-AS1** | **1.51** |  |  |  |
| **182** | **LINC01138** | **1.51** |  |  |  |
| **183** | **LINC01160** | **1.48** |  |  |  |
| **184** | **RP5-996D20.3** | **1.48** |  |  |  |
| **185** | **RP5-1006K12.1** | **1.43** |  |  |  |
| **186** | **FGF14-AS1** | **1.43** |  |  |  |
| **187** | **LINC00092** | **1.43** |  |  |  |
| **188** | **RP5-1115A15.1** | **1.41** |  |  |  |
| **189** | **CYP17A1-AS1** | **1.41** |  |  |  |
| **190** | **ATP11A-AS1** | **1.34** |  |  |  |
| **191** | **LINC01054** | **1.31** |  |  |  |
| **192** | **ZEB1-AS1** | **1.30** |  |  |  |
| **193** | **LINC00475** | **1.28** |  |  |  |
| **194** | **RP5-1147A1.2** | **1.28** |  |  |  |
| **195** | **LINC00272** | **1.26** |  |  |  |
| **196** | **LINC00710** | **1.24** |  |  |  |
| **197** | **F10-AS1** | **1.20** |  |  |  |
| **198** | **LINC00845** | **1.19** |  |  |  |
| **199** | **LINC01168** | **1.13** |  |  |  |
| **200** | **LINC00261** | **1.12** |  |  |  |
| **201** | **LINC00687** | **1.10** |  |  |  |
| **202** | **LINC00840** | **1.09** |  |  |  |
| **203** | **SBF2-AS1** | **1.09** |  |  |  |
| **204** | **LINC00857** | **1.09** |  |  |  |
| **205** | **LINC01116** | **1.08** |  |  |  |
| **206** | **TMEM72-AS1** | **1.00** |  |  |  |
| **207** | **IBA57-AS1** | **0.99** |  |  |  |
| **208** | **GPC5-AS1** | **0.97** |  |  |  |
| **209** | **ZRANB2-AS1** | **0.92** |  |  |  |
| **210** | **LOC100421166** | **0.91** |  |  |  |
| **211** | **FLVCR1-AS1** | **0.84** |  |  |  |
| **212** | **KRTAP5-AS1** | **0.82** |  |  |  |
| **213** | **LINC00264** | **0.80** |  |  |  |
| **214** | **PRMT5-AS1** | **0.76** |  |  |  |
| **215** | **RP5-1112D6.4** | **0.69** |  |  |  |
| **216** | **LINC00629** | **0.67** |  |  |  |
| **217** | **LINC01134** | **0.66** |  |  |  |
| **218** | **LINC00709** | **0.57** |  |  |  |
| **219** | **FAM212B-AS1** | **0.56** |  |  |  |
| **220** | **RP5-988G15.1** | **0.56** |  |  |  |
| **221** | **TBX5-AS1** | **0.50** |  |  |  |
| **222** | **LINC00302** | **0.49** |  |  |  |
| **223** | **RP5-894D12.3** | **0.46** |  |  |  |
| **224** | **LINC01103** | **0.46** |  |  |  |
| **225** | **LINC00582** | **0.45** |  |  |  |
| **226** | **RP5-945F2.2** | **0.41** |  |  |  |
| **227** | **NOVA1-AS1** | **0.37** |  |  |  |
| **228** | **MRGPRG-AS1** | **0.32** |  |  |  |
| **229** | **RP5-1039K5.13** | **0.30** |  |  |  |
| **230** | **MYCBP2-AS1** | **0.27** |  |  |  |
| **231** | **LINC00535** | **0.26** |  |  |  |
| **232** | **LINC00839** | **0.23** |  |  |  |
| **233** | **LINC00278** | **0.22** |  |  |  |
| **234** | **ENOX1-AS1** | **0.21** |  |  |  |
| **235** | **FARP1-AS1** | **0.20** |  |  |  |
| **236** | **RUSC1-AS1** | **0.20** |  |  |  |
| **237** | **LINC00309** | **0.18** |  |  |  |
| **238** | **LINC01037** | **0.15** |  |  |  |
| **239** | **LINC00269** | **0.13** |  |  |  |
| **240** | **NEXN-AS1** | **0.13** |  |  |  |
| **241** | **SMIM2-AS1** | **0.13** |  |  |  |
| **242** | **RNASEH2B-AS1** | **0.12** |  |  |  |
| **243** | **NDFIP2-AS1** | **0.12** |  |  |  |
| **244** | **HLA-AS1** | **0.09** |  |  |  |
| **245** | **STK24-AS1** | **0.09** |  |  |  |
| **246** | **PDCD4-AS1** | **0.09** |  |  |  |
| **247** | **GCSAML-AS1** | **0.06** |  |  |  |
| **248** | **LINC00858** | **0.05** |  |  |  |
| **249** | **RMST** | **0.01** |  |  |  |
| **250** | **FLG-AS1** | **-0.01** |  |  |  |
| **251** | **LINC00890** | **-0.02** |  |  |  |
| **252** | **CACNA1C-AS1** | **-0.03** |  |  |  |
| **253** | **CLYBL-AS1** | **-0.06** |  |  |  |
| **254** | **LINC01137** | **-0.10** |  |  |  |
| **255** | **MAPKAPK5-AS1** | **-0.11** |  |  |  |
| **256** | **LINC00051** | **-0.13** |  |  |  |
| **257** | **LOC401913** | **-0.13** |  |  |  |
| **258** | **DLG5-AS1** | **-0.16** |  |  |  |
| **259** | **RP5-983L19.2** | **-0.16** |  |  |  |
| **260** | **LINC01133** | **-0.17** |  |  |  |
| **261** | **DDX39B-AS1** | **-0.20** |  |  |  |
| **262** | **LINC00626** | **-0.21** |  |  |  |
| **263** | **LINC00657** | **-0.22** |  |  |  |
| **264** | **ARHGEF7-AS1** | **-0.23** |  |  |  |
| **265** | **RP5-991C6.2** | **-0.24** |  |  |  |
| **266** | **OVCH1-AS1** | **-0.24** |  |  |  |
| **267** | **LINC00701** | **-0.26** |  |  |  |
| **268** | **LINC01102** | **-0.28** |  |  |  |
| **269** | **LINC00894** | **-0.29** |  |  |  |
| **270** | **MYO16-AS1** | **-0.29** |  |  |  |
| **271** | **LINC00861** | **-0.31** |  |  |  |
| **272** | **VIM-AS1** | **-0.31** |  |  |  |
| **273** | **SNAP47-AS1** | **-0.34** |  |  |  |
| **274** | **LINC01153** | **-0.38** |  |  |  |
| **275** | **FAM53B-AS1** | **-0.39** |  |  |  |
| **276** | **LOC440461** | **-0.45** |  |  |  |
| **277** | **ADARB2-AS1** | **-0.49** |  |  |  |
| **278** | **RP5-905H7.7** | **-0.50** |  |  |  |
| **279** | **ARAP1-AS1** | **-0.51** |  |  |  |
| **280** | **LINC01124** | **-0.53** |  |  |  |
| **281** | **LINC01090** | **-0.53** |  |  |  |
| **282** | **LINC00502** | **-0.58** |  |  |  |
| **283** | **LINC00116** | **-0.67** |  |  |  |
| **284** | **CPB2-AS1** | **-0.68** |  |  |  |
| **285** | **CLDN10-AS1** | **-0.71** |  |  |  |
| **286** | **RP5-1189B24.1** | **-0.74** |  |  |  |
| **287** | **KIRREL3-AS1** | **-0.75** |  |  |  |
| **288** | **LINC01125** | **-0.75** |  |  |  |
| **289** | **SUCLA2-AS1** | **-0.76** |  |  |  |
| **290** | **LINC01117** | **-0.79** |  |  |  |
| **291** | **LINC00608** | **-0.79** |  |  |  |
| **292** | **LINC00339** | **-0.82** |  |  |  |
| **293** | **RP5-1046G13.2** | **-0.83** |  |  |  |
| **294** | **AC018730.4,LINC01159** | **-0.85** |  |  |  |
| **295** | **PITRM1-AS1** | **-0.86** |  |  |  |
| **296** | **COL4A2-AS1** | **-0.87** |  |  |  |
| **297** | **RP5-1077H22.1** | **-0.89** |  |  |  |
| **298** | **LINC00700** | **-0.91** |  |  |  |
| **299** | **ELOVL2-AS1** | **-0.92** |  |  |  |
| **300** | **LINC00652** | **-0.94** |  |  |  |
| **301** | **CD81-AS1** | **-1.02** |  |  |  |
| **302** | **LINC00630** | **-1.02** |  |  |  |
| **303** | **LINC00999** | **-1.03** |  |  |  |
| **304** | **WASF3-AS1** | **-1.03** |  |  |  |
| **305** | **LINC00467** | **-1.06** |  |  |  |
| **306** | **LINC00568** | **-1.06** |  |  |  |
| **307** | **LINC00299** | **-1.08** |  |  |  |
| **308** | **NKX2-1-AS1** | **-1.08** |  |  |  |
| **309** | **LINC00704** | **-1.10** |  |  |  |
| **310** | **RP5-856G1.1** | **-1.11** |  |  |  |
| **311** | **RASSF8-AS1** | **-1.13** |  |  |  |
| **312** | **RASAL2-AS1** | **-1.15** |  |  |  |
| **313** | **RP5-826L7.1** | **-1.18** |  |  |  |
| **314** | **RP5-894D12.5** | **-1.19** |  |  |  |
| **315** | **LINC00237** | **-1.21** |  |  |  |
| **316** | **CD27-AS1** | **-1.22** |  |  |  |
| **317** | **LINC00961** | **-1.26** |  |  |  |
| **318** | **CHRM3-AS1** | **-1.26** |  |  |  |
| **319** | **GAS6-AS1** | **-1.26** |  |  |  |
| **320** | **LINC00243** | **-1.26** |  |  |  |
| **321** | **RP5-855F16.1** | **-1.29** |  |  |  |
| **322** | **RAB30-AS1** | **-1.32** |  |  |  |
| **323** | **RP5-1166F10.1** | **-1.33** |  |  |  |
| **324** | **TMPRSS4-AS1** | **-1.38** |  |  |  |
| **325** | **LINC01031** | **-1.38** |  |  |  |
| **326** | **CDH23-AS1** | **-1.41** |  |  |  |
| **327** | **LINC00263** | **-1.41** |  |  |  |
| **328** | **MRPL23-AS1** | **-1.43** |  |  |  |
| **329** | **SCEL-AS1** | **-1.43** |  |  |  |
| **330** | **LINC00265-3P** | **-1.44** |  |  |  |
| **331** | **LINC01107** | **-1.46** |  |  |  |
| **332** | **JRKL-AS1** | **-1.48** |  |  |  |
| **333** | **STT3A-AS1** | **-1.49** |  |  |  |
| **334** | **RP5-1186N24.3** | **-1.54** |  |  |  |
| **335** | **A2ML1-AS1** | **-1.54** |  |  |  |
| **336** | **NUTM2A-AS1** | **-1.55** |  |  |  |
| **337** | **RP5-874C20.3** | **-1.56** |  |  |  |
| **338** | **RP5-894D12.4** | **-1.57** |  |  |  |
| **339** | **DAB1-AS1** | **-1.59** |  |  |  |
| **340** | **RP5-857K21.2** | **-1.63** |  |  |  |
| **341** | **NALCN-AS1** | **-1.66** |  |  |  |
| **342** | **NEBL-AS1** | **-1.67** |  |  |  |
| **343** | **TMEM51-AS1** | **-1.71** |  |  |  |
| **344** | **GPR123-AS1** | **-1.77** |  |  |  |
| **345** | **NFYC-AS1** | **-1.85** |  |  |  |
| **346** | **RP5-991C6.3** | **-1.89** |  |  |  |
| **347** | **PARD3-AS1** | **-1.93** |  |  |  |
| **348** | **ALKBH3-AS1** | **-2.05** |  |  |  |
| **349** | **A2M-AS1** | **-2.07** |  |  |  |
| **350** | **AGAP2-AS1** | **-2.16** |  |  |  |
| **351** | **LINC00035** | **-2.18** |  |  |  |
| **352** | **LINC00200** | **-2.27** |  |  |  |
| **353** | **TCERG1L-AS1** | **-2.31** |  |  |  |
| **354** | **RP13-279N23.2** | **-2.40** |  |  |  |
| **355** | **IDI2-AS1** | **-2.43** |  |  |  |
| **356** | **SZT2-AS1** | **-2.45** |  |  |  |
| **357** | **DDX11-AS1** | **-2.50** |  |  |  |
| **358** | **SSSCA1-AS1** | **-2.53** |  |  |  |
| **359** | **TMEM5-AS1** | **-2.53** |  |  |  |
| **360** | **KCND3-AS1** | **-2.64** |  |  |  |
| **361** | **RP5-1170D6.1** | **-2.64** |  |  |  |
| **362** | **FREM2-AS1** | **-2.68** |  |  |  |
| **363** | **MIR600HG** | **-2.71** |  |  |  |
| **364** | **RP5-945F2.1** | **-2.73** |  |  |  |
| **365** | **ATE1-AS1** | **-2.73** |  |  |  |
| **366** | **ATP8A2P1,LINC00993** | **-2.75** |  |  |  |
| **367** | **RP5-991C6.4** | **-2.76** |  |  |  |
| **368** | **LINC00487** | **-2.78** |  |  |  |
| **369** | **TBCE-AS1** | **-2.79** |  |  |  |
| **370** | **LINC00982** | **-2.79** |  |  |  |
| **371** | **TMEM9B-AS1** | **-2.81** |  |  |  |
| **372** | **LGALS8-AS1** | **-2.87** |  |  |  |
| **373** | **HOXC-AS1** | **-2.91** |  |  |  |
| **374** | **LINC01149** | **-2.93** |  |  |  |
| **375** | **AP4B1-AS1** | **-2.97** |  |  |  |
| **376** | **MTUS2-AS1** | **-3.02** |  |  |  |
| **377** | **LINC00280** | **-3.08** |  |  |  |
| **378** | **SRGAP2-AS1** | **-3.08** |  |  |  |
| **379** | **MTOR-AS1** | **-3.13** |  |  |  |
| **380** | **LINC00210** | **-3.15** |  |  |  |
| **381** | **LINC00537** | **-3.21** |  |  |  |
| **382** | **GNG12-AS1** | **-3.21** |  |  |  |
| **383** | **LINC00471** | **-3.23** |  |  |  |
| **384** | **SERTAD4-AS1** | **-3.28** |  |  |  |
| **385** | **RP5-1077H22.2** | **-3.35** |  |  |  |
| **386** | **CELF2-AS1** | **-3.42** |  |  |  |
| **387** | **RP5-902P8.10** | **-3.44** |  |  |  |
| **388** | **LINC00892** | **-3.45** |  |  |  |
| **389** | **LINC00599** | **-3.45** |  |  |  |
| **390** | **USP12-AS1** | **-3.58** |  |  |  |
| **391** | **RP5-1168A5.1** | **-3.58** |  |  |  |
| **392** | **RP5-1052M9.4** | **-3.68** |  |  |  |
| **393** | **FOXD2-AS1** | **-3.68** |  |  |  |
| **394** | **LINC00102** | **-3.80** |  |  |  |
| **395** | **LINC00028** | **-3.82** |  |  |  |
| **396** | **LEMD1-AS1** | **-3.82** |  |  |  |
| **397** | **CACNA2D3-AS1** | **-3.86** |  |  |  |
| **398** | **RP5-1185I7.1** | **-3.92** |  |  |  |
| **399** | **LINC01106** | **-3.97** |  |  |  |
| **400** | **LINC00543** | **-4.01** |  |  |  |
| **401** | **LINC00484** | **-4.03** |  |  |  |
| **402** | **ZMYM4-AS1** | **-4.13** |  |  |  |
| **403** | **DNMBP-AS1** | **-4.14** |  |  |  |
| **404** | **SLC16A1-AS1** | **-4.16** |  |  |  |
| **405** | **LINC00265-2P** | **-4.20** |  |  |  |
| **406** | **EDRF1-AS1** | **-4.22** |  |  |  |
| **407** | **LINC00632** | **-4.27** |  |  |  |
| **408** | **WAC-AS1** | **-4.28** |  |  |  |
| **409** | **LINC00607** | **-4.33** |  |  |  |
| **410** | **LINC01120** | **-4.51** |  |  |  |
| **411** | **LINC00032** | **-4.58** |  |  |  |
| **412** | **LINC00494** | **-4.68** |  |  |  |
| **413** | **LINC00533** | **-4.70** |  |  |  |
| **414** | **LINC01128** | **-4.82** |  |  |  |
| **415** | **RP5-1186P10.2** | **-4.86** |  |  |  |
| **416** | **TBC1D4-AS1** | **-4.97** |  |  |  |
| **417** | **LINC00844** | **-5.00** |  |  |  |
| **418** | **LINC00279** | **-5.20** |  |  |  |
| **419** | **DLEU7-AS1** | **-5.27** |  |  |  |
| **420** | **GRM5-AS1** | **-5.35** |  |  |  |
| **421** | **OVOL1-AS1** | **-5.38** |  |  |  |
| **422** | **RP5-928E24.2** | **-5.39** |  |  |  |
| **423** | **RERG-AS1** | **-5.57** |  |  |  |
| **424** | **ACTA2-AS1** | **-5.57** |  |  |  |
| **425** | **LINC00624** | **-5.58** |  |  |  |
| **426** | **RP5-874C20.6** | **-5.63** |  |  |  |
| **427** | **MCF2L-AS1** | **-5.67** |  |  |  |
| **428** | **LINC00841** | **-5.83** |  |  |  |
| **429** | **GPR158-AS1** | **-5.85** |  |  |  |
| **430** | **USP2-AS1** | **-5.90** |  |  |  |
| **431** | **C1RL-AS1** | **-6.03** |  |  |  |
| **432** | **LINC01057,PGBD4P7** | **-6.08** |  |  |  |
| **433** | **HTR2A-AS1** | **-6.18** |  |  |  |
| **434** | **RP5-899B16.1** | **-6.22** |  |  |  |
| **435** | **FRY-AS1** | **-6.35** |  |  |  |
| **436** | **LINC01101** | **-6.41** |  |  |  |
| **437** | **RP5-905H7.4** | **-6.54** |  |  |  |
| **438** | **LINC01015** | **-6.55** |  |  |  |
| **439** | **RP5-857K21.7** | **-6.65** |  |  |  |
| **440** | **LINC00536** | **-6.66** |  |  |  |
| **441** | **DIAPH3-AS1** | **-6.77** |  |  |  |
| **442** | **VAV3-AS1** | **-6.79** |  |  |  |
| **443** | **SLC25A30-AS1** | **-6.79** |  |  |  |
| **444** | **LINC00867** | **-6.86** |  |  |  |
| **445** | **RP5-859M6.1** | **-6.93** |  |  |  |
| **446** | **RP5-1086L22.1** | **-6.99** |  |  |  |
| **447** | **GRTP1-AS1** | **-7.00** |  |  |  |
| **448** | **TOLLIP-AS1** | **-7.04** |  |  |  |
| **449** | **RP5-1042K10.10** | **-7.30** |  |  |  |
| **450** | **CASC0** | **-7.36** |  |  |  |
| **451** | **PAN3-AS1** | **-7.57** |  |  |  |
| **452** | **RP5-890O3.3** | **-7.86** |  |  |  |
| **453** | **LINC00086** | **-7.93** |  |  |  |
| **454** | **LINC00276** | **-8.06** |  |  |  |
| **455** | **TP73-AS1** | **-8.08** |  |  |  |
| **456** | **LINC00977** | **-8.09** |  |  |  |
| **457** | **PDX1-AS1** | **-8.12** |  |  |  |
| **458** | **GAS5-AS1** | **-8.18** |  |  |  |
| **459** | **LINC00202-1** | **-8.34** |  |  |  |
| **460** | **ZBED5-AS1** | **-8.38** |  |  |  |
| **461** | **UBXN10-AS1** | **-8.48** |  |  |  |
| **462** | **SMAD9-AS1** | **-8.65** |  |  |  |
| **463** | **LINC00466** | **-8.81** |  |  |  |
| **464** | **KDM4A-AS1** | **-8.81** |  |  |  |
| **465** | **SMG7-AS1** | **-8.83** |  |  |  |
| **466** | **CALML3-AS1** | **-8.83** |  |  |  |
| **467** | **C20orf166-AS1** | **-9.04** |  |  |  |
| **468** | **LINC01142** | **-9.08** |  |  |  |
| **469** | **DOCK9-AS1** | **-9.30** |  |  |  |
| **470** | **TRHDE-AS1** | **-9.30** |  |  |  |
| **471** | **PCDH9-AS1** | **-9.36** |  |  |  |
| **472** | **CYP4A22-AS1** | **-9.83** |  |  |  |
| **473** | **LINC00087** | **-9.98** |  |  |  |
| **474** | **RP5-1139I1.1** | **-10.26** |  |  |  |
| **475** | **INTS6-AS1,RPS4XP16** | **-10.48** |  |  |  |
| **476** | **SOX21-AS1** | **-10.53** |  |  |  |
| **477** | **CCDC147-AS1** | **-10.84** |  |  |  |
| **478** | **LINC00337** | **-10.99** |  |  |  |
| **479** | **SOCS2-AS1** | **-11.02** |  |  |  |
| **480** | **TINCR** | **-11.12** |  |  |  |
| **481** | **SOX2-OT** | **-11.47** |  |  |  |
| **482** | **EWSAT1** | **-11.49** |  |  |  |
| **483** | **LOC102724064** | **-11.80** |  |  |  |
| **484** | **LOC100507053** | **-14.11** |  |  |  |
