# Supplementary material for: Circulating Long Non-Coding RNAs LINC00324 and LOC100507053 as Potential Liquid Biopsy Markers for Esophageal Squamous Cell Carcinoma: A Pilot Study
Source: Front Oncol. 2022 Feb 14;12:823953. doi: 10.3389/fonc.2022.823953 (PMC8882835; doi:10.3389/fonc.2022.823953)
Supplement: Supplementary file 4 [file Table_2.docx]

| S.No. | lncRNome | RAID v2.0 | Starbase | Common targets |
| --- | --- | --- | --- | --- |
| 1 | **hsa-miR-23a-5p** | [**hsa-miR-615-5p**](http://www.rna-society.org/raid/php_mysql/search.php?searchType=2&p=1&dataset=actor&method=all&sco1=0.0&sco2=1.0&species=all&keyword=hsa-miR-615-5p&category=miRNA&rna=0) | **hsa-miR-516b-5p** | **hsa-miR-493-5p** |
| 2 | **hsa-miR-27b-5p** | [**hsa-miR-4281**](http://www.rna-society.org/raid/php_mysql/search.php?searchType=2&p=1&dataset=actor&method=all&sco1=0.0&sco2=1.0&species=all&keyword=hsa-miR-4281&category=miRNA&rna=0) | **hsa-miR-214-5p** |  |
| 3 | **hsa-miR-33b-3p** | [**hsa-miR-9-5p**](http://www.rna-society.org/raid/php_mysql/search.php?searchType=2&p=1&dataset=actor&method=all&sco1=0.0&sco2=1.0&species=all&keyword=hsa-miR-9-5p&category=miRNA&rna=0) | **hsa-miR-3164** |  |
| 4 | **hsa-miR-3691-3p** | [**hsa-miR-600**](http://www.rna-society.org/raid/php_mysql/search.php?searchType=2&p=1&dataset=actor&method=all&sco1=0.0&sco2=1.0&species=all&keyword=hsa-miR-600&category=miRNA&rna=0) | **hsa-miR-6820-3p** |  |
| 5 | **hsa-miR-378g** | [**hsa-miR-574-3p**](http://www.rna-society.org/raid/php_mysql/search.php?searchType=2&p=1&dataset=actor&method=all&sco1=0.0&sco2=1.0&species=all&keyword=hsa-miR-574-3p&category=miRNA&rna=0) | **hsa-miR-195-5p** |  |
| 6 | **hsa-miR-4476** | [**hsa-miR-5589-5p**](http://www.rna-society.org/raid/php_mysql/search.php?searchType=2&p=1&dataset=actor&method=all&sco1=0.0&sco2=1.0&species=all&keyword=hsa-miR-5589-5p&category=miRNA&rna=0) | **hsa-miR-497-5p** |  |
| 7 | **hsa-miR-4526** | [**hsa-miR-493-5p**](http://www.rna-society.org/raid/php_mysql/search.php?searchType=2&p=1&dataset=actor&method=all&sco1=0.0&sco2=1.0&species=all&keyword=hsa-miR-493-5p&category=miRNA&rna=0) | **hsa-miR-15a-5p** |  |
| 8 | **hsa-miR-4652-3p** | [**hsa-miR-4779**](http://www.rna-society.org/raid/php_mysql/search.php?searchType=2&p=1&dataset=actor&method=all&sco1=0.0&sco2=1.0&species=all&keyword=hsa-miR-4779&category=miRNA&rna=0) | **hsa-miR-16-5p** |  |
| 9 | **hsa-miR-4779** | [**hsa-miR-4652-3p**](http://www.rna-society.org/raid/php_mysql/search.php?searchType=2&p=1&dataset=actor&method=all&sco1=0.0&sco2=1.0&species=all&keyword=hsa-miR-4652-3p&category=miRNA&rna=0) | **hsa-miR-15b-5p** |  |
| 10 | **hsa-miR-493-5p** | [**hsa-miR-4526**](http://www.rna-society.org/raid/php_mysql/search.php?searchType=2&p=1&dataset=actor&method=all&sco1=0.0&sco2=1.0&species=all&keyword=hsa-miR-4526&category=miRNA&rna=0) | **hsa-miR-424-5p** |  |
| 11 | **hsa-miR-5589-5p** | [**hsa-miR-4476**](http://www.rna-society.org/raid/php_mysql/search.php?searchType=2&p=1&dataset=actor&method=all&sco1=0.0&sco2=1.0&species=all&keyword=hsa-miR-4476&category=miRNA&rna=0) | **hsa-miR-6838-5p** |  |
| 12 | **hsa-miR-574-3p** | [**hsa-miR-378g**](http://www.rna-society.org/raid/php_mysql/search.php?searchType=2&p=1&dataset=actor&method=all&sco1=0.0&sco2=1.0&species=all&keyword=hsa-miR-378g&category=miRNA&rna=0) | **hsa-miR-3942-5p** |  |
| 13 | **hsa-miR-600** | [**hsa-miR-3691-3p**](http://www.rna-society.org/raid/php_mysql/search.php?searchType=2&p=1&dataset=actor&method=all&sco1=0.0&sco2=1.0&species=all&keyword=hsa-miR-3691-3p&category=miRNA&rna=0) | **hsa-miR-4703-5p** |  |
| 14 | **hsa-miR-9-5p** | [**hsa-miR-33b-3p**](http://www.rna-society.org/raid/php_mysql/search.php?searchType=2&p=1&dataset=actor&method=all&sco1=0.0&sco2=1.0&species=all&keyword=hsa-miR-33b-3p&category=miRNA&rna=0) | **hsa-miR-151b** |  |
| 15 | **-** | [**hsa-miR-27b-5p**](http://www.rna-society.org/raid/php_mysql/search.php?searchType=2&p=1&dataset=actor&method=all&sco1=0.0&sco2=1.0&species=all&keyword=hsa-miR-27b-5p&category=miRNA&rna=0) | **hsa-miR-151a-5p** |  |
| 16 | **-** | [**hsa-miR-23a-5p**](http://www.rna-society.org/raid/php_mysql/search.php?searchType=2&p=1&dataset=actor&method=all&sco1=0.0&sco2=1.0&species=all&keyword=hsa-miR-23a-5p&category=miRNA&rna=0) | **hsa-miR-6875-5p** |  |
| 17 | **-** | [**hsa-miR-106a-5p**](http://www.rna-society.org/raid/php_mysql/search.php?searchType=2&p=1&dataset=actor&method=all&sco1=0.0&sco2=1.0&species=all&keyword=hsa-miR-106a-5p&category=miRNA&rna=0) | **hsa-miR-3126-5p** |  |
| 18 | **-** | **-** | **hsa-miR-769-5p** |  |
| 19 | **-** | **-** | **hsa-miR-500b-5p** |  |
| 20 | **-** | **-** | **hsa-miR-362-5p** |  |
| 21 | **-** | **-** | **hsa-miR-3200-5p** |  |
| 22 | **-** | **-** | **hsa-miR-628-5p** |  |
| 23 | **-** | **-** | **hsa-miR-214-3p** |  |
| 24 | **-** | **-** | **hsa-miR-761** |  |
| 25 | **-** | **-** | **hsa-miR-3619-5p** |  |
| 26 | **-** | **-** | **hsa-miR-384** |  |
| 27 | **-** | **-** | **hsa-miR-1276** |  |
| 28 | **-** | **-** | **hsa-miR-493-5p** |  |
| 29 | **-** | **-** | **hsa-miR-545-5p** |  |
| 30 | **-** | **-** | **hsa-miR-154-3p** |  |
| 31 | **-** | **-** | **hsa-miR-487a-3p** |  |
| 32 | **-** | **-** | **hsa-miR-512-3p** |  |

**Supplementary Table 2: LINC00324-miRNA targets prediction using various databases**
