# Supplementary material for: Circulating Long Non-Coding RNAs LINC00324 and LOC100507053 as Potential Liquid Biopsy Markers for Esophageal Squamous Cell Carcinoma: A Pilot Study
Source: Front Oncol. 2022 Feb 14;12:823953. doi: 10.3389/fonc.2022.823953 (PMC8882835; doi:10.3389/fonc.2022.823953)
Supplement: Supplementary file 5 [file Table_3.docx]

| Targets from miRDB database  **Supplementary Table 3: miR-493-5p-mRNA target prediction using various databases** | Targets from Starbase database | Targets from TargetScan database | Common targets |
| --- | --- | --- | --- |
| SP3 | **FUNDC2** | **CAMK2N1** | **SP3** |
| RHOT1 | **CCNL2** | **RASL11B** | **SERTAD2** |
| THSD7A | **NADK** | **MBD5** | **ARFGEF1** |
| SERTAD2 | **GNB1** | **SCAMP2** | **RASL11B** |
| ARFGEF1 | **CLSTN1** | **ARID1B** | **CDH11** |
| RASL11B | **EMC1** | **MARS** | **NDNF** |
| GABRA5 | **CAMK2N1** | **GABRA5** | **MBNL2** |
| CDH11 | **HP1BP3** | **CKS2** | **RASAL2** |
| NDNF | **ZNF43`6** | **RGS10** | **NECAB1** |
| MBNL2 | **SRSF10** | **GNG11** | **TMEM170B** |
| RASAL2 | **STPG1** | **CARTPT** | **LRP12** |
| NECAB1 | **AUNIP** | **AC012360.2** | **HIVEP2** |
| TMEM170B | **AHDC1** | **INSM2** | **DACH1** |
| LRP12 | **RPA2** | **SPINT2** | **ATP2A2** |
| HIVEP2 | **SRSF4** | **ZMYM5** | **ARHGAP44** |
| DACH1 | **PUM1** | **TMEM170B** | **SCAMP2** |
| C5orf15 | **CSMD2** | **FUBP1** | **MBD5** |
| ATP2A2 | **SNIP1** | **GAS1** | **ABTB2** |
| ARHGAP44 | **BMP8B** | **ANKRD61** | **SAT1** |
| SCAMP2 | **RIMS3** | **GOLM1** | **PDS5A** |
| MBD5 | **HIVEP3** | **APOO** | **PGRMC2** |
| IFRD1 | **FOXJ3** | **CWC25** | **KPNA4** |
| NXPH1 | **CC2D1B** | **FOXJ3** | **ZFAND5** |
| CADM2 | **ECHDC2** | **NUDT17** | **AFF3** |
| ABTB2 | **HSPB11** | **HNRNPA0** | **LRIG1** |
| EYA1 | **DAB1** | **AP3S1** | **TGFBRAP1** |
| SAT1 | **OMA1** | **BASP1** | **ANK2** |
| PDS5A | **JUN** | **NECAB1** | **AXIN1** |
| PGRMC2 | **SLC35D1** | **VAMP2** | **TSC22D2** |
| PIK3C3 | **GNG12** | **LMO3** | **FUBP1** |
| KPNA4 | **WLS** | **LRP12** | **ZIC2** |
| ZFAND5 | **DEPDC1** | **FAM76B** | **TCF7L2** |
| AFF3 | **AC118549.1** | **SLC25A12** | **KIAA2026** |
| LRIG1 | **USP33** | **METTL21A** | **FBXL3** |
| TGFBRAP1 | **FUBP1** | **TCF7L2** | **DCUN1D1** |
| ANK2 | **SSX2IP** | **NRXN3** | **HIPK1** |
| HMGN1 | **GBP3** | **CDH11** | **NDST1** |
| AXIN1 | **BARHL2** | **PGRMC2** | **PPP1CC** |
| TSC22D2 | **ZNF644** | **PPP1CC** | **DYRK4** |
| MYT1L | **EVI5** | **SP3** | **SH2B3** |
| ANTXR1 | **ARHGAP29** | **HS3ST3B1** | **ANKRD12** |
| GABRA2 | **CNN3** | **NCAPH** | **TIA1** |
| FUBP1 | **SLC25A24** | **BRINP1** | **ATAD2** |
| FOXO1 | **CLCC1** | **UBE2G1** | **CPNE3** |
| ZIC2 | **SORT1** | **ATAD5** | **LRRC8C** |
| TCF7L2 | **LRIF1** | **IL1A** | **NFKBIA** |
| FAM169A | **RSBN1** | **C4orf46** | **SMG1** |
| SSH2 | **TRIM33** | **DCUN1D1** | **UBE2V2** |
| KIAA2026 | **BCAS2** | **ZIC2** | **COG3** |
| FBXL3 | **NRAS** | **TMEM106B** | **MLLT10** |
| ADAM23 | **CD58** | **ACTR1B** | **MEF2C** |
| SLC2A13 | **GDAP2** | **TPM3** | **DHX36** |
| DCUN1D1 | **TBX15** | **PAPSS2** | **SALL1** |
| HIPK1 | **WARS2** | **BTG1** | **AP3S1** |
| NDST1 | **MCL1** | **CHRM2** | **CITED2** |
| PPP1CC | **HORMAD1** | **PITX2** | **ATAD2B** |
| DYRK4 | **CTSS** | **ZNF532** | **SNRPB2** |
| GRID2 | **PI4KB** | **AHDC1** | **SNX9** |
| SH2B3 | **POGZ** | **PKIA** | **TMEM106B** |
| ANKRD12 | **S100A6** | **CITED2** | **FZD4** |
| TIA1 | **GATAD2B** | **ZFAND5** | **SIN3A** |
| ATAD2 | **TPM3** | **FLJ00104** | **HIF1A** |
| CPNE3 | **UBE2Q1** | **MEF2C** | **ZNF384** |
| LRRC8C | **SHC1** | **MYCBP2** | **ARHGAP12** |
| PCGF5 | **CLK2** | **SUV39H1** | **ITGB1** |
| NFKBIA | **ASH1L** | **KIF18A** | **GTF2IRD1** |
| SMG1 | **MEX3A** | **PVRL3** | **GOLM1** |
| NECTIN3 | **ETV3** | **TSC22D2** | **ENDOD1** |
| UBE2V2 | **NECTIN4** | **GATA2** | **AHDC1** |
| COG3 | **ALDH9A1** | **NUDCD1** | **LARP4** |
| TMEFF1 | **TMCO1** | **BRD7** | **PSD3** |
| MLLT10 | **SCYL3** | **CPLX2** | **RBBP5** |
| GDF6 | **ASTN1** | **GTF2IRD1** | **LIMCH1** |
| SEC24B | **ABL2** | **NCL** | **CWC25** |
| MEF2C | **TOR1AIP2** | **DLL1** | **GSKIP** |
| DHX36 | **GLUL** | **MRPL49** | **DLG2** |
| VPS35L | **PTGS2** | **TSLP** | **CDH2** |
| SALL1 | **UCHL5** | **PTCH1** | **GDNF** |
| EOMES | **DENND1B** | **FAM83B** | **HSPA12A** |
| AP3S1 | **ZNF281** | **ANKRD17** | **BCOR** |
| CITED2 | **KIF14** | **TNFAIP8** | **FOXJ3** |
| ATAD2B | **DDX59** | **TSPAN3** | **DLL1** |
| SNRPB2 | **KIF21B** | **WBP11** | **ARID1B** |
| SNX9 | **ARL8A** | **MLLT10** | **GAS1** |
| AP4S1 | **KDM5B** | **GART** | **PAPSS2** |
| MEGF11 | **RBBP5** | **GCLC** | **IL1A** |
| TMEM106B | **NUAK2** | **PHF2** | **HMGCR** |
| FZD4 | **IRF6** | **DACH1** | **E2F3** |
| SIN3A | **NEK2** | **SAT1** | **REEP1** |
| PLCB1 | **LPGAT1** | **DYRK4** | **MED13L** |
| HIF1A | **TMEM206** | **USMG5** | **WNT5A** |
| ZNF384 | **ESRRG** | **NDNF** | **PIK3R1** |
| ARHGAP12 | **WDR26** | **LRIG1** | **PTCH1** |
| ITGB1 | **ENAH** | **PPP3CA** | **RALGPS1** |
| GTF2IRD1 | **ACBD3** | **GPR176** | **AKAP1** |
| PCGF2 | **ITPKB** | **PRDM12** | **HS3ST3B1** |
| PDZRN3 | **CCSAP** | **YIPF4** | **FBRSL1** |
| MAP3K20 | **NUP133** | **UCK2** | **KIAA1671** |
| GOLM1 | **C1orf131** | **CCNG2** | **PHF12** |
| TAB3 | **EGLN1** | **STT3B** | **RPS6KA5** |
| ENDOD1 | **SIPA1L2** | **HMGXB4** | **CTDSPL2** |
| CDC14A | **TOMM20** | **KIAA0408** | **MGAT3** |
| SLAIN2 | **ARID4B** | **MBNL2** | **NEO1** |
| CALD1 | **NID1** | **ITGB1** | **BTG1** |
| AHDC1 | **GREM2** | **PIK3R1** | **CXADR** |
| SNRNP48 | **CEP170** | **SRPK2** | **PUM2** |
| UNC50 | **TFB2M** | **KIAA2026** | **TTC7B** |
| LARP4 | **ZNF695** | **REEP1** | **EPHA7** |
| MEF2A | **CAMTA1** | **EOMES** | **CARM1** |
| GP6 | **SPSB1** | **PVRL4** | **KCNK1** |
| PSD3 | **KIF1B** | **GRID2** | **PRKD3** |
| RBBP5 | **VPS13D** | **PFN2** | **VPS13D** |
| HOOK3 | **PDPN** | **ARPC3** | **TBC1D22B** |
| KIF13A | **KAZN** | **AC012215.1** | **TCF4** |
| BAZ1A | **SPEN** | **MAML3** | **UBE2G1** |
| MBTD1 | **PNRC2** | **TNFSF11** | **PAG1** |
| LIMCH1 | **ARID1A** | **DDX50** | **CAMK2N1** |
| CWC25 | **PPP1R8** | **ATP5B** | **KDM2B** |
| AGTPBP1 | **XKR8** | **SERPINB7** | **SRGAP1** |
| GSKIP | **ZNF362** | **BTG2** | **NCAPH** |
| NLK | **AGO1** | **PARVA** | **TNFSF11** |
| REV3L | **AKIRIN1** | **HIVEP2** | **SP1** |
| ATP5F1A | **MACF1** | **AAR2** | **CLSTN1** |
| DLG2 | **RLF** | **SNRPB2** | **CREB1** |
| ZNF354C | **RNF220** | **GSKIP** | **GPR176** |
| CDH2 | **NSUN4** | **DDX5** | **FMNL2** |
| BOD1L1 | **BTF3L4** | **SRSF3** | **SVEP1** |
| PRDM8 | **ZYG11B** | **TM2D3** | **BASP1** |
| DSCAML1 | **NFIA** | **AXIN1** | **DIP2C** |
| CLOCK | **LEPROT** | **NAB1** | **PAIP1** |
| ZBTB38 | **SRSF11** | **TIA1** | **CYTH3** |
| ZNF560 | **FPGT** | **BCL7A** | **ACAP2** |
| ARHGEF38 | **TYW3** | **CDH2** | **NAV2** |
| GDNF | **ADGRL2** | **MCTP1** | **GNPTAB** |
| HSPA12A | **LMO4** | **UBE2E2** | **BBX** |
| BCOR | **PKN2** | **SH2B3** | **HP1BP3** |
| FOXJ3 | **LRRC8B** | **KIAA0247** | **SLC22A23** |
| PSMG4 | **LRRC8C** | **RGS17** | **VAMP2** |
| DLL1 | **EPHX4** | **KCNK1** | **EFNA3** |
| ARID1B | **AGL** | **MAP7** | **ZMIZ1** |
| GAS1 | **MFSD14A** | **NIPBL** | **ANKRD17** |
| PAPSS2 | **AC118553.2** | **RALGPS1** | **NRXN3** |
| DEFB132 | **CDC14A** | **ANK2** | **FBXW11** |
| ZNF720 | **SLC30A7** | **PCNP** | **FNIP1** |
| IL1A | **PRPF38B** | **EED** | **PPP2R2C** |
| HMGCR | **STXBP3** | **TRAF3IP2** | **DAB2IP** |
| ANO4 | **GNAI3** | **APBB1IP** | **PPP2R5D** |
| C1GALT1C1 | **CEPT1** | **CTDSPL2** | **HK2** |
| SRI | **MAGI3** | **IGSF1** | **PREX1** |
| E2F3 | **HIPK1** | **PDE7B** | **BMPR1A** |
| REEP1 | **VANGL1** | **CSPP1** | **MAP3K2** |
| MED13L | **GABPB2** | **SPIRE2** | **VASH2** |
| WNT5A | **IVL** | **ATP5A1** | **CELF2** |
| GRM5 | **EFNA3** | **BCOR** | **CKS2** |
| PIK3R1 | **IFI16** | **RORA** | **MYCBP2** |
| GTF2H1 | **VANGL2** | **GREM2** | **PDPK1** |
| PTCH1 | **USP21** | **RASSF8** | **SRPX** |
| RALGPS1 | **DUSP12** | **NEO1** | **KDM6A** |
| DUSP16 | **NOS1AP** | **CPSF1** | **NCL** |
| VSTM2B | **UAP1** | **UBE2V2** | **USP7** |
| LTBP3 | **PBX1** | **PDK4** | **YIPF4** |
| HECTD2 | **UCK2** | **ZMYND8** | **KIF18A** |
| PMP22 | **POU2F1** | **ZNF706** | **NPAS3** |
| AKAP1 | **TIPRL** | **ZNF384** | **STAM** |
| OSBPL6 | **ATP1B1** | **RNF214** | **TNRC6B** |
| HS3ST3B1 | **PRRX1** | **KPNA4** | **ATAD5** |
| C1QL1 | **DNM3** | **GPC4** | **NAB1** |
| FBRSL1 | **RABGAP1L** | **CACNB2** | **RAPGEF2** |
| CLIC2 | **RASAL2** | **AKAP6** | **PANK3** |
| EPSTI1 | **CDC73** | **IRX3** | **FBXO33** |
| KIAA1671 | **CFHR3** | **SNX9** | **DENND4C** |
| INSM2 | **NEK7** | **STK24** | **BCL7A** |
| ANKRD61 | **PTPRC** | **CPNE3** | **MED13** |
| PHF12 | **CAMSAP2** | **NFKBIA** | **NUDT4** |
| MTSS1 | **ELF3** | **GABRG2** | **TRIP10** |
| RAP1GAP2 | **BTG2** | **GDF6** | **RGS10** |
| RPS6KA5 | **ATP2B4** | **SERTAD2** | **NCOA7** |
| CLIP1 | **CDK18** | **STAM** | **SEC63** |
| F3 | **PFKFB2** | **PAIP1** | **MAP3K1** |
| CTDSPL2 | **SERTAD4** | **HEMGN** | **LMO3** |
| ZNF431 | **FLVCR1** | **TGFBRAP1** | **ZNF148** |
| MGAT3 | **VASH2** | **ZNF281** | **ZNF385B** |
| NEO1 | **RRP15** | **KLF3** | **SCRN1** |
| BTG1 | **MARK1** | **PSD2** | **STARD13** |
| COQ3 | **STUM** | **SALL1** | **GJC1** |
| SOS2 | **SNAP47** | **HSPD1** | **ZMYND8** |
| LYRM7 | **RHOU** | **P4HA1** | **ARHGAP5** |
| TRHDE | **TSNAX** | **ACTR10** | **GATA2** |
| LDLRAD4 | **KCNK1** | **DHX36** | **NCOA1** |
| ADCY1 | **SLC35F3** | **CELF2** | **PURB** |
| CXADR | **KIF26B** | **DAB2IP** | **ITPRIPL2** |
| PUM2 | **DIP2C** | **BRMS1L** | **UCK2** |
| LDLR | **USP6NL** | **PDS5A** | **WASF3** |
| ZNF718 | **SKIDA1** | **PBX3** | **SCN1A** |
| HOXA1 | **PIP4K2A** | **ELF1** | **MBNL3** |
| PPFIA1 | **ABI1** | **POU4F1** | **CTNND2** |
| ELAVL1 | **ANKRD26** | **GPR63** | **RAB5C** |
| TTC7B | **SVIL** | **ARHGAP44** | **ZNF638** |
| CENPJ | **MTPAP** | **PRPF38B** | **HMGCS1** |
| SLC38A2 | **ARHGAP12** | **LIN52** | **IRX3** |
| EPHA7 | **KIF5B** | **MAP4K3** | **ACTR1B** |
| JPH3 | **EPC1** | **DAB1** | **SLC10A7** |
| CARM1 | **ITGB1** | **RPL29** | **TNFAIP8** |
| KCNK1 | **CUL2** | **ATAD2** | **SLC25A12** |
| PRKD3 | **FZD8** | **TTC7B** | **L1CAM** |
| VPS13D | **ZNF33B** | **IRF1** | **NR2C2** |
| TBC1D22B | **ERCC6** | **CLSTN1** | **CSPP1** |
| EEA1 | **PARG** | **XPO7** | **TMEM170A** |
| TCF4 | **CCDC6** | **SRPX** | **RAB10** |
| UBE2G1 | **EGR2** | **CLPX** | **KAT6A** |
| ZNF43 | **JMJD1C** | **RPE** | **HMGXB4** |
| PAG1 | **DNAJC12** | **RFTN2** | **SHOC2** |
| CAMK2N1 | **P4HA1** | **LRRC8C** | **DIP2A** |
| SUSD6 | **DNAJC9** | **GAD1** | **GCLC** |
| KDM2B | **PPP3CB** | **DRGX** | **HNRNPA0** |
| SRGAP1 | **ATAD1** | **VASH2** | **STT3B** |
| GGACT | **PANK1** | **ZMYND19** | **BRD7** |
| LOC102724951 | **ANKRD1** | **PDGFC** | **NUDCD1** |
| NCAPH | **PPP1R3C** | **LRRC55** | **RDX** |
| TNFSF11 | **CPEB3** | **MXD1** | **THBS1** |
| SP1 | **TM9SF3** | **ATP2A2** | **MCTP1** |
| C1S | **MMS19** | **HIF1A** | **KDM3A** |
| RAB27B | **GOT1** | **OVOL1** | **SMARCC1** |
| ESF1 | **CHUK** | **GNB1** | **ZNF532** |
| CLSTN1 | **ATP5MD** | **SHC3** | **BTAF1** |
| ASTN1 | **GPAM** | **GJC1** | **DNAJC13** |
| NDST3 | **CCDC186** | **DIP2C** | **ESRRG** |
| TBK1 | **GFRA1** | **GRAMD1B** | **APOO** |
| CREB1 | **HSPA12A** | **SLC26A4** | **NUDT21** |
| GPR176 | **FAM204A** | **CHSY3** | **ZNF800** |
| LOC102723360 | **RGS10** | **EPHA7** | **CASC4** |
| LOC102724219 | **TIAL1** | **SIN3A** | **GPR63** |
| RAB3IP | **FAM196A** | **HMGCS1** | **PPP3CA** |
| ERC2 | **MKI67** | **USP2** | **FXR1** |
| FMNL2 | **EBF3** | **MED26** | **LZTS2** |
| FAR1 | **ZMYND11** | **ARHGAP12** | **POU4F1** |
| LOC102724843 | **WDR37** | **FBXL3** | **TMEM178B** |
| TMEM128 | **NET1** | **TRAM2** | **SUV39H1** |
| FAM114A1 | **TAF3** | **ADNP** | **KDELR2** |
| SVEP1 | **GATA3** | **SBNO1** | **THAP11** |
| BASP1 | **CELF2** | **PPP1R10** | **COL8A1** |
| DIP2C | **SUV39H2** | **HK2** | **DMD** |
| PAIP1 | **STAM** | **ZNF385B** | **PSMD11** |
| CYTH3 | **CACNB2** | **FGF19** | **CTNND1** |
| CAPZA2 | **MLLT10** | **VSIG1** | **ANKRD50** |
| ACAP2 | **BMI1** | **MAPK1** | **FOXO3** |
| BCL11A | **OTUD1** | **CHUK** | **SCAMP5** |
| ATP5MD | **RAB18** | **EMILIN2** | **GNB1** |
| NAV2 | **WAC** | **DNAJC14** | **RREB1** |
| SYT4 | **MAP3K8** | **LARP4** | **GOLGA1** |
| MYO9B | **CCNY** | **RNF181** | **TAOK3** |
| ZNF91 | **REEP3** | **HMGCR** | **FAM133B** |
| SORCS3 | **SIRT1** | **PPP2R2C** | **SPRYD3** |
| GNPTAB | **DDX50** | **MGAT3** | **GPC4** |
| SLC5A1 | **CHST3** | **ST5** | **WBP11** |
| BBX | **KAT6B** | **TRIP10** | **HSPD1** |
| RSL24D1 | **ZMIZ1** | **B3GAT2** | **STK4** |
| SPARCL1 | **GHITM** | **FBXW11** | **SH2D3C** |
| ITGBL1 | **BMPR1A** | **TMEM26** | **SRPK2** |
| HP1BP3 | **PAPSS2** | **RNF125** | **KDM5B** |
| COLEC12 | **PTEN** | **CASC4** | **BRMS1L** |
| STXBP5L | **IFIT2** | **C18orf25** | **OVOL1** |
| SLC22A23 | **PCGF5** | **MEMO1** | **MAP1B** |
| ZDHHC17 | **HECTD2** | **TNKS2** | **ZFHX3** |
| VAMP2 | **TNKS2** | **KIAA1024** | **ZNF704** |
| FAXC | **BTAF1** | **NPAS3** | **DGKD** |
| SERPINB7 | **KIF11** | **VPS35** | **ESRP2** |
| EFNA3 | **EXOC6** | **EVI5** | **FAM76B** |
| ZMIZ1 | **HELLS** | **SVEP1** | **SPAST** |
| ANKRD17 | **LCOR** | **ABR** | **SYNE1** |
| NRXN3 | **FRAT1** | **CADM2** | **ZNF706** |
| FBXW11 | **SLF2** | **SOX1** | **MED17** |
| FNIP1 | **LZTS2** | **ZNF618** | **DNAJC21** |
| NUDT16 | **BTRC** | **SMC6** | **MAP4K3** |
| BNC2 | **GBF1** | **VSX2** | **FLT3LG** |
| PPP2R2C | **SUFU** | **L1CAM** | **RASGRP1** |
| WNK3 | **CNNM2** | **ROR2** | **PARVA** |
| WDR33 | **SLK** | **EIF5A2** | **DNAJC14** |
| DAB2IP | **RBM20** | **AFF3** | **CLASP1** |
| PPP2R5D | **SHOC2** | **KCNAB1** | **KCTD15** |
| HK2 | **TCF7L2** | **MTPAP** | **STK24** |
| GABRG3 | **NHLRC2** | **ING1** | **GABRA1** |
| KCNJ13 | **AL162407.1** | **NR1D2** | **MRPL49** |
| USP32 | **FAM160B1** | **MTDH** | **SLC35E1** |
| PREX1 | **ATRNL1** | **CCNT2** | **ETV5** |
| BMPR1A | **EDRF1** | **ITPRIPL2** | **PHF2** |
| RABGAP1L | **PTPRE** | **TMPO** | **SPEN** |
| TMEM237 | **VENTX** | **LMBR1** | **INTS8** |
| MAP3K2 | **SCART1** | **ABTB2** | **CHSY3** |
| VASH2 | **BET1L** | **THBS1** | **SUFU** |
| CELF2 | **INS-IGF2** | **SCN1A** | **TBC1D30** |
| MFAP5 | **IGF2** | **SLC10A7** | **GART** |
| CKS2 | **NAP1L4** | **ROPN1** | **BAZ1B** |
| TIAL1 | **OR51E2** | **NCOA7** | **ELMO2** |
| MYCBP2 | **ST5** | **FMNL2** | **ABR** |
| PIAS1 | **DENND5A** | **GRM5** | **CHUK** |
| AKR1D1 | **RRAS2** | **ELF3** | **FLRT2** |
| FAM228A | **SOX6** | **NDST1** | **REEP5** |
| PDPK1 | **PIK3C2A** | **KCNJ6** | **CCDC93** |
| SRPX | **E2F8** | **COG3** | **DNAJA2** |
| GABRG2 | **LGR4** | **RNF185** | **MADD** |
| SUN1 | **KIF18A** | **MAML2** | **TMTC1** |
| KDM6A | **ABTB2** | **FLT3LG** | **WWC1** |
| NCL | **NUP160** | **NRDE2** | **RAB2A** |
| USP21 | **AHNAK** | **KIAA1671** | **TANC2** |
| USP7 | **SYVN1** | **CSNK1A1** | **MON2** |
| CUL5 | **KMT5B** | **ENDOD1** | **ZMYND19** |
| KIAA0408 | **FGF19** | **BHLHE41** | **NRDE2** |
| RSPO2 | **RSF1** | **RAPGEF2** | **VGLL3** |
| YIPF4 | **DLG2** | **SCYL1** | **EIF4B** |
| CDH13 | **CREBZF** | **MALT1** | **WDR43** |
| KIF18A | **FZD4** | **SLIT2** | **MAML3** |
| RXRA | **FAM76B** | **PBX1** | **KLF3** |
| CRISP3 | **MAML2** | **MED17** | **CACNB2** |
| NPAS3 | **CCDC82** | **NETO1** | **SLC6A6** |
| STAM | **EXPH5** | **ZNF800** | **P4HA1** |
| TNRC6B | **RDX** | **RAB5C** | **PRPF38B** |
| ATAD5 | **PPP2R1B** | **USP7** | **TNKS2** |
| MIER3 | **USP2** | **NSG2** | **PIKFYVE** |
| AK5 | **ARHGAP32** | **ZMIZ2** | **NRK** |
| NAB1 | **PRDM10** | **TBC1D22B** | **PBX3** |
| EIF4ENIF1 | **ZBTB44** | **NSL1** | **MARCKS** |
| PRDM12 | **TRIM22** | **WNT5A** | **UBE2E2** |
| LOC100506388 | **TIMM10B** | **IKZF1** | **ELF1** |
| LCA5 | **RPL27A** | **DNAJC13** | **SYNE2** |
| RAPGEF2 | **IPO7** | **ANO4** | **CAMSAP2** |
| UNC5C | **ZNF143** | **ACAP2** | **FGF7** |
| PANK3 | **SWAP70** | **MADD** | **PPP2R5C** |
| DGKI | **USP47** | **CTNND2** | **RBM26** |
| FBXO33 | **PARVA** | **FAM133B** | **ANP32B** |
| HAT1 | **FAR1** | **CTDNEP1** | **C16orf70** |
| DENND4C | **GTF2H1** | **DKK2** | **PDE7B** |
| EGR2 | **NAV2** | **SCRN1** | **PPP1R10** |
| SPRY3 | **QSER1** | **ELMO2** | **TRPS1** |
| RAD21 | **PDHX** | **CASD1** | **MED1** |
| ALCAM | **API5** | **RASAL2** | **USP8** |
| TDO2 | **HSD17B12** | **ZFX** | **RORA** |
| MT2A | **MADD** | **GREM1** | **GREM2** |
| TMCO3 | **PTPRJ** | **BMPR1A** | **SRSF3** |
| BCL7A | **CTNND1** | **KDELR2** | **ANKRD52** |
| MED13 | **OTUB1** | **DNAJA2** | **RASSF8** |
| 7-Sep | **FLRT1** | **SERTM1** | **DDX50** |
| PHC3 | **MRPL49** | **SLC35E1** | **NETO2** |
| CALN1 | **SCYL1** | **UBE2Q1** | **FBXL4** |
| ACKR4 | **OVOL1** | **SCRIB** | **EVI5** |
| DOCK9 | **NDUFS8** | **KDM2B** | **ADNP** |
| GJB7 | **PPP6R3** | **FBXO33** | **RNF125** |
| KLF4 | **PPFIA1** | **SPRYD3** | **AAR2** |
| M6PR | **ARHGEF17** | **CYTH3** | **MAP7** |
| NUDT4 | **RPS3** | **PAG1** | **SPRED2** |
| JUN | **EMSY** | **SYNE1** | **TMPO** |
| ALX1 | **ACER3** | **MTSS1** | **BTG2** |
| PARP8 | **EED** | **THAP11** | **CSRNP3** |
| ALDH9A1 | **PRSS23** | **KAZN** | **KIF2A** |
| USP43 | **MED17** | **PDE4D** | **ZFX** |
| ITPRID2 | **AP001273.2** | **ETV3** | **GAD1** |
| NAMPT | **FUT4** | **TOP2B** | **PPTC7** |
| CYFIP2 | **AMOTL1** | **C3orf80** | **SOCS7** |
| TRIP10 | **ENDOD1** | **EIF4E** | **PDK4** |
| SF3B1 | **JRKL** | **AKAP1** | **MAML2** |
| KCNJ6 | **CUL5** | **SP4** | **DGKH** |
| HSF5 | **SIK2** | **LZTS2** | **KIF1B** |
| RGS10 | **RBM7** | **GPR37L1** | **GABPB2** |
| NCOA7 | **PAFAH1B2** | **FGF2** | **USP6NL** |
| SEC63 | **RNF214** | **CARM1** | **PITX2** |
| PHF20L1 | **KMT2A** | **STK4** | **SLIT2** |
| MAP3K1 | **ARCN1** | **XKR4** | **SCYL1** |
| MTREX | **SORL1** | **PPP2R5C** | **MTDH** |
| LMO3 | **UBASH3B** | **AK5** | **TPM3** |
| ZNF148 | **GRAMD1B** | **GGACT** | **MARK1** |
| ZNF385B | **EI24** | **FBRS** | **TRAM2** |
| SCRN1 | **APLP2** | **ZNF436** | **DNMT3A** |
| USP16 | **ST14** | **GNPTAB** | **LMBR1** |
| IL15 | **LPAR5** | **SCAMP5** | **MAPK1** |
| STARD13 | **ZNF384** | **GIT1** | **SPINT2** |
| GJC1 | **C1RL** | **ANP32B** | **SERTAD4** |
| NTN4 | **SLC2A14** | **TCF21** | **NR1D2** |
| ZMYND8 | **SLC2A3** | **SPAST** | **UBE2Q1** |
| NDUFC2 | **MFAP5** | **PDPK1** | **PTPRD** |
| TOX3 | **M6PR** | **OSBPL6** | **CUL3** |
| RMC1 | **LRP6** | **NUDT4** | **TRAF3IP2** |
| ARHGAP5 | **DUSP16** | **EGFR** | **TSPAN3** |
| KRBOX1 | **WBP11** | **ZZEF1** | **CCNG2** |
| OSBPL8 | **LMO3** | **GDNF** | **RNF214** |
| TMCO1 | **SLCO1A2** | **SFXN5** | **CLPX** |
| GATA2 | **SOX5** | **HIPK1** | **BHLHE41** |
| NCOA1 | **KRAS** | **SHOC2** | **FGF19** |
| PURB | **BHLHE41** | **FAM63B** | **ROR2** |
| CPEB2 | **TMTC1** | **TCF4** | **CRISPLD2** |
| ITPRIPL2 | **CPNE8** | **MPP6** | **GNG11** |
| LCE1E | **SLC2A13** | **RASD1** | **DDX5** |
| UCK2 | **NELL2** | **HTR2A** | **KIF24** |
| MCUR1 | **SLC38A1** | **FAM178A** | **PPP3CB** |
| FRMD4A | **SLC38A2** | **ZBTB11** | **ZNF281** |
| ZNF236 | **AMIGO2** | **KCNK5** | **SETD1A** |
| WASF3 | **ADCY6** | **PCDH18** | **SF3B3** |
| RNF4 | **KMT2D** | **PLEKHA8** | **GALNT10** |
| RAPH1 | **PRKAG1** | **KIF24** | **USP2** |
| ARID4B | **FMNL3** | **CDC73** | **PCNP** |
| FAM84A | **CSRNP2** | **NMT1** |  |
| SERP1 | **SPRYD3** | **TBC1D4** |  |
| CENPA | **DNAJC14** | **PLEKHA7** |  |
| NPR3 | **ANKRD52** | **SLC35D1** |  |
| ADARB2 | **GLS2** | **IGSF9B** |  |
| IQCK | **BAZ2A** | **ANKRD12** |  |
| TP63 | **ATP5F1B** | **NAA25** |  |
| SCN1A | **PTGES3** | **COBLL1** |  |
| SEC24D | **DCTN2** | **OPCML** |  |
| KLHL24 | **C12orf66** | **E2F3** |  |
| MBNL3 | **GRIP1** | **PREX1** |  |
| CTNND2 | **NAP1L1** | **MAP3K2** |  |
| KLHL36 | **OSBPL8** | **TMED7** |  |
| GRHL2 | **PPP1R12A** | **GABRA1** |  |
| DMGDH | **KITLG** | **CXXC4** |  |
| KALRN | **POC1B** | **FNIP1** |  |
| FAM217B | **GALNT4** | **DENND4C** |  |
| LANCL2 | **POC1B-GALNT4** | **ELMO1** |  |
| RAB5C | **BTG1** | **ANKRD50** |  |
| C11orf87 | **EEA1** | **UNC5D** |  |
| SPX | **UBE2N** | **PCNX** |  |
| ZNF638 | **NTN4** | **AGFG1** |  |
| MEMO1 | **CDK17** | **SF3B3** |  |
| HMGCS1 | **IKBIP** | **PDPN** |  |
| RTN4IP1 | **GNPTAB** | **RAD52** |  |
| SMAD9 | **ANAPC7** | **KCND3** |  |
| IRX3 | **ARPC3** | **UBP1** |  |
| ADAM28 | **PPTC7** | **PPP3CB** |  |
| PUM1 | **HVCN1** | **RYBP** |  |
| CXCL8 | **PPP1CC** | **GALNT10** |  |
| ACTR1B | **ATXN2** | **RAB2A** |  |
| OTUD1 | **NAA25** | **SNIP1** |  |
| ZMYM5 | **MED13L** | **SYVN1** |  |
| SLC10A7 | **FBXO21** | **TMEM170A** |  |
| NECTIN4 | **TAOK3** | **KDM6A** |  |
| HORMAD1 | **CAMKK2** | **PPM1A** |  |
| DCTN2 | **KDM2B** | **SLC22A23** |  |
| DENND5A | **CLIP1** | **SRSF11** |  |
| PTGER3 | **ZCCHC8** | **MYLK2** |  |
| TNFAIP8 | **PITPNM2** | **MYT1L** |  |
| SMAP1 | **SBNO1** | **FGF7** |  |
| SLC25A12 | **STX2** | **CHMP1B** |  |
| CACNB4 | **GOLGA3** | **CTNND1** |  |
| VSX2 | **ZNF605** | **KCTD15** |  |
| L1CAM | **WNK1** | **HNRNPA3** |  |
| HAPLN1 | **DYRK4** | **HSPA12A** |  |
| DENND4A | **NDUFA9** | **NLGN3** |  |
| NR2C2 | **C1S** | **KCNJ10** |  |
| CSPP1 | **RIMKLB** | **KDM5B** |  |
| MCM6 | **PLEKHA5** | **FBXO32** |  |
| TMEM170A | **ETNK1** | **HMX3** |  |
| BTBD3 | **RASSF8** | **PANK3** |  |
| KLK2 | **BICD1** | **TMEM178B** |  |
| CAMK4 | **DNM1L** | **HELZ** |  |
| PLEKHA5 | **LARP4** | **TRHDE** |  |
| FER | **DIP2B** | **SRSF10** |  |
| RAB10 | **ACVR1B** | **MON2** |  |
| MPP7 | **NR4A1** | **LRRTM3** |  |
| RFX3 | **EIF4B** | **POU3F1** |  |
| HS6ST3 | **SP1** | **SMG1** |  |
| ZNF143 | **HOXC9** | **C19orf44** |  |
| STON2 | **ORMDL2** | **DLG2** |  |
| KAT6A | **MARS** | **THSD7A** |  |
| HMGXB4 | **KIF5A** | **TAOK3** |  |
| SHOC2 | **SLC16A7** | **UBE2F** |  |
| ZC3H12B | **MON2** | **SEPT7** |  |
| RAB11FIP2 | **SRGAP1** | **EIF4B** |  |
| JPH1 | **TBK1** | **GRIN3A** |  |
| POLK | **RASSF3** | **PPP1R2** |  |
| KIAA0825 | **TBC1D30** | **PPP2R5D** |  |
| NIN | **LEMD3** | **FBXO21** |  |
| DIP2A | **HMGA2** | **ASXL2** |  |
| CDH10 | **DYRK2** | **KIF2A** |  |
| GCLC | **RAP1B** | **HERPUD2** |  |
| PLGLB2 | **RAB3IP** | **PPTC7** |  |
| HNRNPA0 | **NAV3** | **VPS13D** |  |
| STT3B | **NUDT4** | **LRWD1** |  |
| FLVCR1 | **ELK3** | **MED13L** |  |
| BRINP1 | **NEDD1** | **ENAH** |  |
| BRD7 | **TMPO** | **GFRA2** |  |
| ADGRL3 | **DRAM1** | **HHIP** |  |
| SLC13A1 | **WASHC4** | **MED1** |  |
| CAV2 | **TMEM263** | **SETD7** |  |
| ARID1A | **UBE3B** | **SYNE2** |  |
| NUDCD1 | **ATP2A2** | **KDM3A** |  |
| PITPNB | **SH2B3** | **COL8A1** |  |
| EXOC6 | **OAS3** | **EFNA3** |  |
| ADAMTS3 | **OAS2** | **MFN1** |  |
| RDX | **RFC5** | **WASF3** |  |
| ASPH | **UNC119B** | **PDE1C** |  |
| THBS1 | **BCL7A** | **RRP1B** |  |
| FBXO38 | **DENR** | **SIPA1L3** |  |
| MCTP1 | **DDX55** | **WDR37** |  |
| KDM3A | **GTF2H3** | **MAP3K1** |  |
| BACH2 | **AACS** | **PARP8** |  |
| PLGLB1 | **RAN** | **FAM49B** |  |
| LRP11 | **ADGRD1** | **PUM2** |  |
| SMARCC1 | **FBRSL1** | **SP1** |  |
| CD24 | **ZNF10** | **YY1** |  |
| RCHY1 | **ZNF268** | **USP14** |  |
| ZNF532 | **XPO4** | **TCTE1** |  |
| CLEC19A | **SACS** | **CXADR** |  |
| CASS4 | **USP12** | **ARFGEF1** |  |
| ZDHHC21 | **SLC7A1** | **UNC5C** |  |
| PCDHA8 | **UBL3** | **KIF1B** |  |
| BTAF1 | **HMGB1** | **ZNF148** |  |
| PCDHAC1 | **STARD13** | **TLK1** |  |
| DNAJC13 | **SMAD9** | **NDUFC2** |  |
| CCN2 | **SUPT20H** | **CAMSAP2** |  |
| SLC17A5 | **FOXO1** | **GBF1** |  |
| PCDHAC2 | **ELF1** | **SMARCC1** |  |
| PCDHA13 | **KBTBD7** | **KCNIP4** |  |
| FEZF2 | **TSC22D1** | **EXPH5** |  |
| ESRRG | **MED4** | **MBD2** |  |
| APOO | **INTS6** | **TBC1D8** |  |
| PCDHA10 | **PCDH9** | **SH2D3C** |  |
| PCDHA5 | **DACH1** | **DCUN1D3** |  |
| TIGAR | **MZT1** | **NUDT21** |  |
| PCDHA6 | **KLF12** | **ADARB2** |  |
| ATG3 | **TBC1D4** | **SETD1A** |  |
| PCDHA11 | **FBXL3** | **KCMF1** |  |
| RRAS2 | **MYCBP2** | **HNRNPDL** |  |
| PCDHA3 | **POU4F1** | **USP8** |  |
| PCDHA9 | **RBM26** | **RREB1** |  |
| JMJD1C | **SPRY2** | **ADAM23** |  |
| FGL2 | **STK24** | **ASB7** |  |
| PCDHA4 | **DOCK9** | **RBM23** |  |
| PCDHA1 | **ZIC5** | **FUT9** |  |
| PCDHA7 | **EFNB2** | **WDR43** |  |
| PCDHA2 | **IRS2** | **TMTC1** |  |
| PCDHA12 | **COL4A1** | **KIAA1432** |  |
| NLGN3 | **SPATA13** | **STIM2** |  |
| GPM6A | **WASF3** | **ZC3H12B** |  |
| ZNF10 | **PAN3** | **LPP** |  |
| NUDT21 | **NBEA** | **ESRRG** |  |
| ZNF800 | **WBP4** | **FZD3** |  |
| ZNF441 | **NAA16** | **BTAF1** |  |
| CASC4 | **DGKH** | **ZNF704** |  |
| GPR63 | **AKAP11** | **MAP1B** |  |
| PDIA5 | **TNFSF11** | **TRPS1** |  |
| PPP3CA | **GTF2F2** | **UBN2** |  |
| CERKL | **COG3** | **OTUB1** |  |
| FXR1 | **RB1** | **NCOA1** |  |
| ST20-MTHFS | **FNDC3A** | **USP32** |  |
| LZTS2 | **CDADC1** | **HP1BP3** |  |
| DIP2B | **TRIM13** | **GNB4** |  |
| POU4F1 | **SLITRK5** | **ADAMTSL1** |  |
| TMEM178B | **MBNL2** | **ENTPD6** |  |
| IRF2 | **ZIC2** | **PRKCA** |  |
| SUV39H1 | **ING1** | **EXT1** |  |
| SLC25A43 | **ARHGEF7** | **MYCL** |  |
| KDELR2 | **ATP11A** | **TMEM151B** |  |
| C3orf35 | **LAMP1** | **RASGRP1** |  |
| ZFR | **TMCO3** | **FXR1** |  |
| ANKS4B | **PIP4P1** | **SERTAD4** |  |
| MSANTD3-TMEFF1 | **RBM23** | **RBM26** |  |
| MTHFS | **DTD2** | **PIKFYVE** |  |
| THAP11 | **BAZ1A** | **DNAJC21** |  |
| COL8A1 | **NFKBIA** | **ZC3H6** |  |
| LRRC8B | **TRAPPC6B** | **PURG** |  |
| TMOD1 | **FBXO33** | **TANC2** |  |
| ZNF737 | **KLHL28** | **LZTS1** |  |
| CYP24A1 | **SOS2** | **VANGL2** |  |
| DMD | **SAV1** | **NMNAT2** |  |
| NID1 | **PYGL** | **REEP5** |  |
| KLHL13 | **DDHD1** | **DGKI** |  |
| PSMD11 | **DCAF5** | **KAT6A** |  |
| ZNF649 | **ELMSAN1** | **ABHD17A** |  |
| TM6SF1 | **ACYP1** | **PURB** |  |
| UBE2A | **STON2** | **FOXO3** |  |
| TMEM199 | **SEL1L** | **SMARCA5** |  |
| TRIM61 | **NRDE2** | **VGLL3** |  |
| CTNND1 | **TTC7B** | **C11orf30** |  |
| ANKRD50 | **RPS6KA5** | **BBX** |  |
| FOXO3 | **CCDC88C** | **NR2C2** |  |
| ANAPC10 | **TRIP11** | **SLC6A6** |  |
| SCAMP5 | **ATXN3** | **TRIM2** |  |
| GNB1 | **BTBD7** | **SLC7A14** |  |
| RREB1 | **ATG2B** | **FZD4** |  |
| GOLGA1 | **BCL11B** | **ANKRD52** |  |
| TAOK3 | **CCDC85C** | **ERN1** |  |
| FAM135A | **JAG2** | **NETO2** |  |
| SOX1 | **G2E3** | **CREB1** |  |
| ATL2 | **COCH** | **TNRC6B** |  |
| FAM133B | **AP4S1** | **GPRIN3** |  |
| HOPX | **ARHGAP5** | **CMTM4** |  |
| MRTFA | **NPAS3** | **PRKD3** |  |
| CUL1 | **BRMS1L** | **SETD9** |  |
| CCDC88A | **PAX9** | **SLC25A26** |  |
| NBEA | **C14orf28** | **HIAT1** |  |
| PRELID3B | **PRPF39** | **ABI2** |  |
| NFAT5 | **ATL1** | **PAPLN** |  |
| CCDC102B | **CDKN3** | **CAMKK2** |  |
| LAMP2 | **SAMD4A** | **ONECUT2** |  |
| SFXN1 | **FBXO34** | **ANKRD28** |  |
| SPRYD3 | **NAA30** | **FBXL4** |  |
| NELL2 | **ACTR10** | **RAB10** |  |
| ACTR5 | **DAAM1** | **RPS6KA3** |  |
| TJP1 | **PPM1A** | **TM9SF3** |  |
| GPC4 | **HIF1A** | **ETF1** |  |
| WBP11 | **SYNE2** | **STARD13** |  |
| HSPD1 | **PLEKHG3** | **SSBP2** |  |
| ARFIP1 | **GPHN** | **TOR1AIP2** |  |
| ZNF714 | **MPP5** | **FBRSL1** |  |
| STK4 | **SUSD6** | **MED13** |  |
| NR4A1 | **TTC9** | **YARS2** |  |
| SH2D3C | **PCNX1** | **TFR2** |  |
| SLC25A36 | **DNAL1** | **SPEN** |  |
| SRPK2 | **LIN52** | **RDX** |  |
| ZBED4 | **JDP2** | **EML6** |  |
| KDM5B | **NRXN3** | **FAT3** |  |
| ZNF98 | **FLRT2** | **ZMIZ1** |  |
| FREM2 | **TTC8** | **SDC2** |  |
| BRMS1L | **CALM1** | **SETD1B** |  |
| BHLHE22 | **GOLGA5** | **DDX3X** |  |
| PROX1 | **GSKIP** | **BAZ2A** |  |
| RAB3C | **PAPOLA** | **ZBTB7A** |  |
| HDGFL3 | **VRK1** | **C16orf70** |  |
| SCN2A | **CCNK** | **SCML2** |  |
| OVOL1 | **YY1** | **STX16** |  |
| MAP1B | **PPP2R5C** | **IMPACT** |  |
| ZFHX3 | **WDR20** | **RAB11FIP1** |  |
| ZNF704 | **EIF5** | **PSD3** |  |
| XKR8 | **KIF26A** | **JAKMIP2** |  |
| KDM5C | **NIPA1** | **PHF12** |  |
| PTPN4 | **UBE3A** | **SRGAP1** |  |
| GTPBP8 | **TJP1** | **GRIN2A** |  |
| DGKD | **LPCAT4** | **ATAD2B** |  |
| ESRP2 | **ACTC1** | **ARHGAP5** |  |
| FAM76B | **DPH6** | **GOLGA1** |  |
| FBXO5 | **RASGRP1** | **LRP6** |  |
| FAM83B | **GPR176** | **CUL3** |  |
| SKA2 | **TTBK2** | **FAM179A** |  |
| VHL | **UBR1** | **PAX9** |  |
| ZNF211 | **GABPB1** | **ESRP2** |  |
| USP12 | **MYO5A** | **SOX5** |  |
| BRCA1 | **RSL24D1** | **MBNL3** |  |
| SPAST | **RAB27A** | **PDE11A** |  |
| UNC5A | **DNAAF4** | **NEGR1** |  |
| SYNE1 | **RFX7** | **SCYL3** |  |
| BRD4 | **ADAM10** | **RPS6KA5** |  |
| TRIM33 | **SLTM** | **PIK3C3** |  |
| ST6GAL2 | **RORA** | **MTMR3** |  |
| ZNF706 | **HERC1** | **FGD4** |  |
| MET | **SPG21** | **ZFHX3** |  |
| ELOVL5 | **CLPX** | **PTPRD** |  |
| MED17 | **INTS14** | **DGKD** |  |
| PLAG1 | **SCAMP2** | **BPTF** |  |
| NUP133 | **SIN3A** | **CCDC93** |  |
| OPCML | **TSPAN3** | **LPHN3** |  |
| CCDC84 | **TBC1D2B** | **ATG14** |  |
| SWT1 | **HDGFL3** | **UBE3A** |  |
| TGFBI | **IDH2** | **CCNY** |  |
| IQCJ-SCHIP1 | **LINS1** | **SLC8A1** |  |
| TET2 | **SELENOS** | **CNNM2** |  |
| DNAJC21 | **TM2D3** | **DIP2A** |  |
| MAP4K3 | **TARSL2** | **SOCS5** |  |
| SEC24A | **TUBGCP5** | **SUFU** |  |
| FLT3LG | **KLF13** | **LPCAT1** |  |
| RASGRP1 | **ARHGAP11A** | **TMEM132B** |  |
| APBB2 | **GREM1** | **ARHGEF17** |  |
| PARVA | **SPRED1** | **ZZZ3** |  |
| SCHIP1 | **THBS1** | **RUNX1T1** |  |
| COX10 | **PAK6** | **ROBO2** |  |
| CADPS | **BUB1B-PAK6** | **HIVEP3** |  |
| RAD51C | **SNAP23** | **SV2B** |  |
| ISPD | **PDIA3** | **FRMPD3** |  |
| PACRGL | **CASC4** | **FLNB** |  |
| DNAJC14 | **CTDSPL2** | **PITPNM2** |  |
| SPTBN1 | **EID1** | **ATXN2** |  |
| CLASP1 | **FGF7** | **NFATC2** |  |
| KCTD15 | **DTWD1** | **LAMP1** |  |
| UGT8 | **USP8** | **GRIN2B** |  |
| SNX14 | **MAPK6** | **TTN** |  |
| FUT9 | **POLR2M** | **NLGN1** |  |
| STK24 | **MINDY2** | **BAZ1B** |  |
| FCHO2 | **TLN2** | **PDGFA** |  |
| CTHRC1 | **RAB8B** | **RNF150** |  |
| ZNF33B | **ZNF609** | **DENR** |  |
| CFAP57 | **HACD3** | **MARK1** |  |
| PGAP1 | **PIAS1** | **STAT5B** |  |
| CARTPT | **GLCE** | **SEL1L** |  |
| BCL2 | **KIF23** | **CRTC1** |  |
| PSMG2 | **ARIH1** | **ZNF516** |  |
| RLIM | **NEO1** | **CYFIP2** |  |
| SHISA6 | **SCAMP5** | **LMTK2** |  |
| HBS1L | **UBE2Q2** | **METTL8** |  |
| ELL | **MORF4L1** | **SRF** |  |
| BMP8A | **KIAA1024** | **DGKH** |  |
| POC1B | **ABHD17C** | **MFSD6** |  |
| FAM122B | **CEMIP** | **NAV2** |  |
| ANKRD1 | **NR2F2** | **C8orf59** |  |
| ZNF492 | **SYNM** | **AGO1** |  |
| SNAP23 | **MEF2A** | **DIMT1** |  |
| GABRA1 | **AXIN1** | **EIF5B** |  |
| CCDC173 | **RNPS1** | **DMD** |  |
| NMBR | **CREBBP** | **CLASP1** |  |
| LRRTM3 | **USP7** | **DCLRE1C** |  |
| C2orf88 | **GRIN2A** | **CENPP** |  |
| STX6 | **RRN3** | **MAP2K6** |  |
| MRPL49 | **XYLT1** | **FAM204A** |  |
| UNC5D | **SMG1** | **LSAMP** |  |
| ARFGEF2 | **DCUN1D3** | **C14orf28** |  |
| SLC35E1 | **USP31** | **FAM189A1** |  |
| CDKN1C | **PALB2** | **INSR** |  |
| ETV5 | **GSG1L** | **KLF7** |  |
| TUBGCP5 | **HIRIP3** | **ARIH1** |  |
| PHF2 | **SHCBP1** | **RXRA** |  |
| NSG2 | **VPS35** | **DNMT3A** |  |
| SDHAF4 | **DNAJA2** | **RIMS3** |  |
| DCAF17 | **NETO2** | **C1orf95** |  |
| NSL1 | **BRD7** | **EPG5** |  |
| SLC26A9 | **SALL1** | **FLRT2** |  |
| UBXN10 | **IRX3** | **ZNF638** |  |
| THOC2 | **NUDT21** | **ETV5** |  |
| SLC9A3R1 | **CDH11** | **TBC1D30** |  |
| SPEN | **CMTM4** | **GABPB2** |  |
| GFRA2 | **DYNC1LI2** | **PRR12** |  |
| INTS8 | **ESRP2** | **WWC1** |  |
| CHSY3 | **ZFHX3** | **CDK12** |  |
| AARD | **TMEM170A** | **PLEKHG3** |  |
| SUFU | **MPHOSPH6** | **PITPNM3** |  |
| NCEH1 | **ZDHHC7** | **FRMPD4** |  |
| HLF | **ZCCHC14** | **SEC63** |  |
| TBC1D30 | **RAB40C** | **LIMCH1** |  |
| GART | **PDPK1** | **SPRED2** |  |
| SMARCA5 | **ZNF263** | **DNM1L** |  |
| BAZ1B | **SNN** | **NUFIP2** |  |
| HIRIP3 | **SHISA9** | **MARCKS** |  |
| ELMO2 | **MKL2** | **GCA** |  |
| ADAMTSL1 | **C16orf45** | **CSRNP3** |  |
| ABR | **COQ7** | **BSN** |  |
| SLC7A1 | **ITPRIPL2** | **NRK** |  |
| WAC | **VPS35L** | **ROCK1** |  |
| GABRA6 | **IQCK** | **ITGA10** |  |
| MAP3K13 | **ANKS4B** | **OSBP2** |  |
| CHUK | **METTL9** | **IPO7** |  |
| KCNQ3 | **MOSMO** | **SHISA6** |  |
| TRIM22 | **PLK1** | **HID1** |  |
| NRP2 | **RBBP6** | **TOX3** |  |
| FLRT2 | **TNRC6A** | **SPNS2** |  |
| PHTF2 | **NFATC2IP** | **CRISPLD2** |  |
| REEP5 | **FBRS** | **ZNF831** |  |
| IWS1 | **FBXL19** | **MARK4** |  |
| KCNJ10 | **SETD1A** | **ZNF641** |  |
| TGFBR1 | **ZNF720** | **TLE4** |  |
| CCDC93 | **CYLD** | **HECW2** |  |
| BANK1 | **CHD9** | **AKAP12** |  |
| FABP3 | **MT2A** | **ZBTB16** |  |
| ZNF879 | **MT1F** | **XPO4** |  |
| DNAJA2 | **GINS3** | **LTBP3** |  |
| MADD | **C16orf70** | **C20orf112** |  |
| CRTAP | **THAP11** | **MEX3A** |  |
| FGF12 | **TANGO6** | **CREBBP** |  |
| COX11 | **NFAT5** | **NRG2** |  |
| C16orf45 | **SF3B3** | **PSMD11** |  |
| ATXN1 | **ZNRF1** | **USP6NL** |  |
| SLK | **CMIP** | **STRN** |  |
| TMTC1 | **CDH13** | **ETNK1** |  |
| WWC1 | **KLHL36** | **SWAP70** |  |
| TAB2 | **CRISPLD2** | **CCDC85C** |  |
| ZEB1 | **JPH3** | **CBX8** |  |
| RAB2A | **ABR** | **BNC2** |  |
| PDGFRA | **ZZEF1** | **TNIK** |  |
| MORC4 | **UBE2G1** | **NOL7** |  |
| TANC1 | **ZNF594** | **LMNB2** |  |
| ENDOU | **CTDNEP1** | **GLUL** |  |
| TANC2 | **CLDN7** | **RBM12** |  |
| SOX2 | **VAMP2** | **RAB3C** |  |
| SLC30A8 | **PMP22** | **TXNDC17** |  |
| PLA1A | **RASD1** | **INTS8** |  |
| AAK1 | **SREBF1** | **HIC2** |  |
| UCHL5 | **SHMT1** | **PIGV** |  |
| MON2 | **PHF12** | **MSH6** |  |
| SSPN | **NUFIP2** | **SOCS7** |  |
| GLRA3 | **GIT1** | **RBBP5** |  |
| LRRN3 | **SSH2** | **VGLL4** |  |
| NCKAP5 | **MYO19** | **ANGEL2** |  |
| PRMT2 | **DDX52** | **POLH** |  |
| GNB4 | **PCGF2** | **NSUN4** |  |
| NEDD4L | **CWC25** | **NADK** |  |
| LELP1 | **MED1** | **FZD5** |  |
| NR4A3 | **SMARCE1** | **ZNF507** |  |
| LHFPL6 | **AC073508.2** | **IGSF10** |  |
| KDM6B | **RAB5C** | **CRTAP** |  |
| SMARCA2 | **STAT5B** | **ORMDL2** |  |
| ZMYND19 | **COA3** | **BACE1** |  |
| RNF180 | **BRCA1** | **FTO** |  |
| NRDE2 | **GJC1** | **CISD3** |  |
| RRN3 | **KIF18B** | **CCDC84** |  |
| CMTM6 | **C1QL1** | **GABRB1** |  |
| AUTS2 | **HOXB2** | **ORC4** |  |
| BCCIP | **HOXB7** | **MSH2** |  |
| DDX46 | **ZNF652** | **ZBTB20** |  |
| GMNC | **SLC35B1** | **MCTS1** |  |
| VGLL3 | **SPAG9** | **FAM64A** |  |
| FZD1 | **MBTD1** | **LRRC40** |  |
| EIF4B | **COX11** |  |  |
| ALG10 | **VEZF1** |  |  |
| WDR43 | **TRIM37** |  |  |
| MAML3 | **SKA2** |  |  |
| KLF3 | **MED13** |  |  |
| WNK1 | **SMARCD2** |  |  |
| PCDH19 | **ERN1** |  |  |
| THAP1 | **DDX5** |  |  |
| SDC2 | **HELZ** |  |  |
| ITSN2 | **HID1** |  |  |
| ZNF117 | **JPT1** |  |  |
| OR13A1 | **USP36** |  |  |
| SVIL | **RBFOX3** |  |  |
| DMXL1 | **CBX8** |  |  |
| ZC3H8 | **RAB40B** |  |  |
| CARD8 | **RPA1** |  |  |
| CACNB2 | **RAP1GAP2** |  |  |
| CUX1 | **ZFP3** |  |  |
| FGFR1OP2 | **USP6** |  |  |
| SLC6A6 | **RPAIN** |  |  |
| PRIMA1 | **KDM6B** |  |  |
| CALML4 | **MAP2K4** |  |  |
| CCL2 | **ARHGAP44** |  |  |
| COQ8A | **HS3ST3B1** |  |  |
| SDHAF3 | **RAI1** |  |  |
| ACSL4 | **SPECC1** |  |  |
| ZNF516 | **WSB1** |  |  |
| P4HA1 | **KSR1** |  |  |
| ATL1 | **NLK** |  |  |
| NMT1 | **TMEM199** |  |  |
| ST8SIA2 | **TAOK1** |  |  |
| ZDHHC23 | **CPD** |  |  |
| FZD7 | **ATAD5** |  |  |
| GPM6B | **RHOT1** |  |  |
| APOLD1 | **ZNF207** |  |  |
| ARHGAP29 | **PSMD11** |  |  |
| ATP2B4 | **SOCS7** |  |  |
| GOLGA8H | **CDK12** |  |  |
| VPS13C | **RAPGEFL1** |  |  |
| GAS2L3 | **RAMP2** |  |  |
| MMUT | **G6PC** |  |  |
| LNPK | **NME1** |  |  |
| PRPF38B | **TOM1L1** |  |  |
| TNKS2 | **HLF** |  |  |
| HYAL4 | **AKAP1** |  |  |
| PIKFYVE | **RAD51C** |  |  |
| ADGRL2 | **TBX2** |  |  |
| NRK | **TANC2** |  |  |
| PBX3 | **PRKCA** |  |  |
| EPS15L1 | **BPTF** |  |  |
| GABRB2 | **C17orf80** |  |  |
| MARCKS | **SLC9A3R1** |  |  |
| 3-Sep | **RNF213** |  |  |
| UBE2E2 | **COLEC12** |  |  |
| INSYN2 | **PIEZO2** |  |  |
| IFI16 | **PTPN2** |  |  |
| PSD2 | **ROCK1** |  |  |
| SLF2 | **ABHD3** |  |  |
| FAM24B | **SS18** |  |  |
| LSM5 | **CDH2** |  |  |
| ELF1 | **GAREM1** |  |  |
| TBL1XR1 | **KLHL14** |  |  |
| SYNE2 | **EPG5** |  |  |
| MPP6 | **ATP5F1A** |  |  |
| MORF4L1 | **HDHD2** |  |  |
| STYK1 | **SMAD2** |  |  |
| CNOT6 | **SMAD7** |  |  |
| ZNF699 | **MEX3C** |  |  |
| CXXC4 | **MBD2** |  |  |
| STAT5A | **TCF4** |  |  |
| CTDSP1 | **TXNL1** |  |  |
| CAMSAP2 | **BCL2** |  |  |
| SUPT20H | **VPS4B** |  |  |
| UBE2D3 | **MBP** |  |  |
| CTSV | **USP14** |  |  |
| FRMD3 | **MTCL1** |  |  |
| FGF7 | **ANKRD12** |  |  |
| FSHB | **RALBP1** |  |  |
| PPP2R5C | **VAPA** |  |  |
| VAV3 | **CHMP1B** |  |  |
| RNPS1 | **GNAL** |  |  |
| GPAM | **PSMG2** |  |  |
| FAAP24 | **LDLRAD4** |  |  |
| POLR2M | **MIB1** |  |  |
| CDKN1A | **RMC1** |  |  |
| RBM26 | **RNF125** |  |  |
| ANP32B | **ASXL3** |  |  |
| C19orf12 | **DTNA** |  |  |
| ATP11A | **FHOD3** |  |  |
| TENM1 | **SETBP1** |  |  |
| C16orf70 | **C18orf25** |  |  |
| FAM47B | **SKA1** |  |  |
| PDHX | **RAB27B** |  |  |
| PDZD2 | **WDR7** |  |  |
| RFTN2 | **ONECUT2** |  |  |
| ZNF273 | **NEDD4L** |  |  |
| RTL8A | **MALT1** |  |  |
| PDZD7 | **ZNF532** |  |  |
| DOCK5 | **SEC11C** |  |  |
| MEI4 | **ZCCHC2** |  |  |
| PDE7B | **ABHD17A** |  |  |
| LIX1 | **LMNB2** |  |  |
| NUP153 | **ZBTB7A** |  |  |
| TBP | **INSR** |  |  |
| TMPRSS11E | **ELAVL1** |  |  |
| RAB40B | **KEAP1** |  |  |
| SOGA3 | **TRIR** |  |  |
| PPP1R10 | **BRD4** |  |  |
| ZNF621 | **SLC35E1** |  |  |
| TMEM132B | **MED26** |  |  |
| TRPS1 | **ELL** |  |  |
| CSNK1A1L | **ZNF708** |  |  |
| MED1 | **ZNF100** |  |  |
| TBX18 | **ZNF98** |  |  |
| ZNF234 | **ZNF91** |  |  |
| FAM9A | **ZNF675** |  |  |
| TCERG1L | **UQCRFS1** |  |  |
| CXCL3 | **C19orf12** |  |  |
| ZNF596 | **ANKRD27** |  |  |
| ERG | **ZNF792** |  |  |
| ACSL3 | **ZNF260** |  |  |
| PLXDC2 | **ZNF585B** |  |  |
| RHOQ | **ZNF780B** |  |  |
| USP8 | **POU2F2** |  |  |
| RORA | **ZNF235** |  |  |
| GREM2 | **ZC3H4** |  |  |
| SRSF3 | **BBC3** |  |  |
| SUCNR1 | **ZNF615** |  |  |
| ANKRD52 | **IL11** |  |  |
| GJA1 | **TRIP10** |  |  |
| CPLX2 | **ZNF559** |  |  |
| BDH1 | **ZNF177** |  |  |
| APPL1 | **ZNF559-ZNF177** |  |  |
| BCL6 | **PDE4A** |  |  |
| RASSF8 | **CARM1** |  |  |
| DDX50 | **LDLR** |  |  |
| NETO2 | **ZNF627** |  |  |
| SRPK1 | **ZNF791** |  |  |
| WDFY3 | **CYP4F3** |  |  |
| FMO2 | **MYO9B** |  |  |
| HERC1 | **ARRDC2** |  |  |
| PCDH7 | **ZNF430** |  |  |
| PCDH11Y | **ZNF431** |  |  |
| LPCAT2 | **ZNF257** |  |  |
| PTPN12 | **ZNF726** |  |  |
| NET1 | **ZNF254** |  |  |
| CPNE8 | **ZNF507** |  |  |
| GRM1 | **FAAP24** |  |  |
| KMT2C | **KCTD15** |  |  |
| LSM8 | **LSM14A** |  |  |
| CDH20 | **UBA2** |  |  |
| PPP2CA | **ZNF302** |  |  |
| NLGN4X | **ZNF793** |  |  |
| GUCA1A | **SIPA1L3** |  |  |
| FBXL4 | **SPINT2** |  |  |
| NCAN | **MARK4** |  |  |
| EVI5 | **FLT3LG** |  |  |
| NRXN1 | **AC010619.1** |  |  |
| TRIM36 | **PRR12** |  |  |
| ADNP | **EMC10** |  |  |
| IPMK | **ZNF331** |  |  |
| STAG1 | **CACNG8** |  |  |
| RNF125 | **ZNF264** |  |  |
| AAR2 | **ZNF805** |  |  |
| MIER1 | **ZNF543** |  |  |
| KIF26A | **ZNF587** |  |  |
| PPP6R3 | **ROCK2** |  |  |
| SCN7A | **E2F6** |  |  |
| MAP7 | **SMC6** |  |  |
| CC2D2B | **PUM2** |  |  |
| GNG5 | **ATAD2B** |  |  |
| SPRED2 | **ITSN2** |  |  |
| CDCA2 | **DNMT3A** |  |  |
| TOB2 | **ASXL2** |  |  |
| TMPO | **MPV17** |  |  |
| PCP4 | **STRN** |  |  |
| BTG2 | **EIF2AK2** |  |  |
| TWSG1 | **PRKD3** |  |  |
| SKIL | **CDC42EP3** |  |  |
| ROCK2 | **ATL2** |  |  |
| PFDN4 | **MAP4K3** |  |  |
| CSRNP3 | **CALM2** |  |  |
| HS3ST5 | **FBXO11** |  |  |
| CDH19 | **CCDC88A** |  |  |
| PTPRS | **EFEMP1** |  |  |
| PTGES3L | **BCL11A** |  |  |
| PRRG4 | **TMEM17** |  |  |
| NAP1L1 | **SERTAD2** |  |  |
| PKHD1 | **SPRED2** |  |  |
| PAFAH1B2 | **PPP3R1** |  |  |
| CDC42EP3 | **GFPT1** |  |  |
| FAM122C | **TIA1** |  |  |
| NFIA | **SNRPG** |  |  |
| KIF2A | **PAIP2B** |  |  |
| ZFX | **EXOC6B** |  |  |
| WASHC4 | **DUSP11** |  |  |
| PAN3 | **POLR1A** |  |  |
| HOXB7 | **REEP1** |  |  |
| TSC22D1 | **SEMA4C** |  |  |
| GAD1 | **ACTR1B** |  |  |
| DIXDC1 | **REV1** |  |  |
| KYAT1 | **AFF3** |  |  |
| CHD9 | **LONRF2** |  |  |
| PPTC7 | **TGFBRAP1** |  |  |
| MINAR1 | **RGPD6** |  |  |
| SOCS7 | **ZC3H8** |  |  |
| GOT1 | **RGPD8** |  |  |
| GOLGA8J | **IL1A** |  |  |
| PDK4 | **CCDC93** |  |  |
| PCDH18 | **CLASP1** |  |  |
| MAK16 | **ERCC3** |  |  |
| GOLGA8M | **MAP3K2** |  |  |
| FGD4 | **WDR33** |  |  |
| WWC3 | **FAM168B** |  |  |
| FGF13 | **NCKAP5** |  |  |
| VDAC1 | **CXCR4** |  |  |
| PCNX1 | **ZEB2** |  |  |
| ZNF248 | **ARL5A** |  |  |
| DAGLB | **CACNB4** |  |  |
| ZNF146 | **PRPF40A** |  |  |
| SCAF8 | **LY75** |  |  |
| CA5B | **RBMS1** |  |  |
| MAML2 | **FIGN** |  |  |
| DGKH | **COBLL1** |  |  |
| RNF187 | **GALNT3** |  |  |
| BCAS1 | **SCN1A** |  |  |
| TSN | **TLK1** |  |  |
| RNF144B | **SLC25A12** |  |  |
| GOLIM4 | **SP3** |  |  |
| SCN8A | **CHN1** |  |  |
| SELENOI | **LNPK** |  |  |
| HMX2 | **ZNF385B** |  |  |
| PGBD3 | **NEMP2** |  |  |
| KIF1B | **PGAP1** |  |  |
| GABPB2 | **SF3B1** |  |  |
| MEIOC | **HSPD1** |  |  |
| ERCC6 | **TMEM237** |  |  |
| CCR5 | **RAPH1** |  |  |
| SIPA1L2 | **KLF7** |  |  |
| TTF1 | **METTL21A** |  |  |
| HVCN1 | **FZD5** |  |  |
| FAT1 | **ERBB4** |  |  |
| PTPRC | **IKZF2** |  |  |
| INPP4A | **WDFY1** |  |  |
| DPF3 | **SERPINE2** |  |  |
| EPHX4 | **CUL3** |  |  |
| LRIF1 | **NCL** |  |  |
| SPIRE2 | **GBX2** |  |  |
| AGA | **IQCA1** |  |  |
| WDR48 | **PASK** |  |  |
| FAM78A | **SOX11** |  |  |
| ATP1B1 | **GRHL1** |  |  |
| IGFBP1 | **NCOA1** |  |  |
| USP6NL | **RAB10** |  |  |
| PDE11A | **SELENOI** |  |  |
| IGSF3 | **KCNK3** |  |  |
| ZNF587 | **CENPA** |  |  |
| FKBP1B | **WDR43** |  |  |
| ST7 | **TOGARAM2** |  |  |
| KIF11 | **SPAST** |  |  |
| GRSF1 | **YIPF4** |  |  |
| ROBO2 | **PRKCE** |  |  |
| PITX2 | **RHOQ** |  |  |
| ABL2 | **SOCS5** |  |  |
| CNTN1 | **MSH6** |  |  |
| SLIT2 | **FOXN2** |  |  |
| APBB1IP | **PEX13** |  |  |
| ARL8B | **C2orf74** |  |  |
| SCYL1 | **ANTXR1** |  |  |
| GPD2 | **GMCL1** |  |  |
| MTDH | **MXD1** |  |  |
| ZNF696 | **ZNF638** |  |  |
| TPM3 | **HK2** |  |  |
| C2CD6 | **KCMF1** |  |  |
| DKK2 | **RNF181** |  |  |
| CDC123 | **KDM3A** |  |  |
| GRIN3A | **NCAPH** |  |  |
| CD58 | **UNC50** |  |  |
| COCH | **EIF5B** |  |  |
| ST6GALNAC5 | **NPAS2** |  |  |
| MARK1 | **CNOT11** |  |  |
| SEZ6L | **C2orf49** |  |  |
| TRAM2 | **RGPD5** |  |  |
| RFC1 | **TMEM87B** |  |  |
| ESYT3 | **ZC3H6** |  |  |
| MIB1 | **SLC20A1** |  |  |
| ATP6V1G3 | **DDX18** |  |  |
| DNMT3A | **INSIG2** |  |  |
| ZNF519 | **PTPN4** |  |  |
| STX11 | **EPB41L5** |  |  |
| IGF2 | **GLI2** |  |  |
| LMBR1 | **TSN** |  |  |
| MAPK1 | **PLEKHB2** |  |  |
| DTD2 | **CCNT2** |  |  |
| YTHDF3 | **R3HDM1** |  |  |
| NDUFB2 | **UBXN4** |  |  |
| ZNF805 | **ACVR2A** |  |  |
| SPINT2 | **MBD5** |  |  |
| SERTAD4 | **KIF5C** |  |  |
| TTC28 | **LYPD6** |  |  |
| NR1D2 | **TNFAIP6** |  |  |
| ARHGEF3 | **FMNL2** |  |  |
| RPS3 | **TANC1** |  |  |
| DNM3 | **CSRNP3** |  |  |
| SLC35F3 | **PPIG** |  |  |
| OR11A1 | **GAD1** |  |  |
| LSM14A | **GORASP2** |  |  |
| UBE2Q1 | **DCAF17** |  |  |
| TRMT9B | **PDK1** |  |  |
| PSENEN | **MAP3K20** |  |  |
| RHOXF2 | **HNRNPA3** |  |  |
| PTPRD | **ZNF804A** |  |  |
| CUL3 | **ITGAV** |  |  |
| TRAF3IP2 | **C2orf88** |  |  |
| SLC26A4 | **MFSD6** |  |  |
| TDRD15 | **NAB1** |  |  |
| GTF2H3 | **COQ10B** |  |  |
| ARHGAP11A | **FZD7** |  |  |
| PIK3R6 | **ABI2** |  |  |
| R3HDM1 | **NRP2** |  |  |
| GRINA | **CREB1** |  |  |
| KCNV1 | **PIKFYVE** |  |  |
| ZBTB33 | **ACSL3** |  |  |
| ZFY | **AGFG1** |  |  |
| SPON1 | **DGKD** |  |  |
| BICD1 | **AGAP1** |  |  |
| ELMO1 | **COPS8** |  |  |
| RHOXF2B | **LRRFIP1** |  |  |
| TEP1 | **ASB1** |  |  |
| DENND1B | **ESF1** |  |  |
| PRUNE2 | **FLRT3** |  |  |
| AP4M1 | **KIF16B** |  |  |
| ZNF367 | **THBD** |  |  |
| TSPAN3 | **NOL4L** |  |  |
| ECM2 | **NCOA6** |  |  |
| CAMK2B | **UQCC1** |  |  |
| TNFRSF10B | **RBM12** |  |  |
| CD83 | **RBL1** |  |  |
| RAD51AP2 | **BLCAP** |  |  |
| TRIM13 | **ELMO2** |  |  |
| LRRFIP1 | **ZMYND8** |  |  |
| CCNG2 | **PREX1** |  |  |
| ETV1 | **SPATA2** |  |  |
| RNF214 | **TMEM189** |  |  |
| POU3F1 | **ADNP** |  |  |
| CLPX | **NFATC2** |  |  |
| TBC1D8 | **ATP9A** |  |  |
| BLM | **ZNF217** |  |  |
| C12orf66 | **AURKA** |  |  |
| ANKRD66 | **TAF4** |  |  |
| VANGL1 | **TCFL5** |  |  |
| TMEM144 | **DIDO1** |  |  |
| TTC8 | **CDS2** |  |  |
| RIMS2 | **CRLS1** |  |  |
| KRT6A | **BMP2** |  |  |
| KNOP1 | **PLCB1** |  |  |
| BHLHE41 | **BTBD3** |  |  |
| GRIP1 | **NDUFAF5** |  |  |
| FGF19 | **SNRPB2** |  |  |
| BAALC | **INSM1** |  |  |
| CDADC1 | **ENTPD6** |  |  |
| MYO5A | **GINS1** |  |  |
| ROR2 | **ZNF341** |  |  |
| SNAI1 | **AAR2** |  |  |
| MRFAP1 | **DLGAP4** |  |  |
| YME1L1 | **STK4** |  |  |
| ATAD1 | **NCOA3** |  |  |
| EPB41L5 | **ARFGEF2** |  |  |
| TNFRSF9 | **SNAI1** |  |  |
| MZT1 | **PARD6B** |  |  |
| CMPK2 | **RAB22A** |  |  |
| GDAP2 | **VAPB** |  |  |
| CEBPZOS | **STX16** |  |  |
| CRISPLD2 | **FAM217B** |  |  |
| PTPN14 | **SS18L1** |  |  |
| ZFP3 | **COL9A3** |  |  |
| KBTBD2 | **NRIP1** |  |  |
| ANOS1 | **MRPL39** |  |  |
| GNG11 | **ATP5PF** |  |  |
| DDX5 | **APP** |  |  |
| KIF24 | **LTN1** |  |  |
| FRMPD3 | **TIAM1** |  |  |
| IKZF2 | **SYNJ1** |  |  |
| RBFA | **TMEM50B** |  |  |
| JAKMIP2 | **GART** |  |  |
| YARS2 | **CRYZL1** |  |  |
| CAMTA1 | **BRWD1** |  |  |
| MRPS21 | **HMGN1** |  |  |
| PPP3CB | **CXADR** |  |  |
| MMP25 | **USP16** |  |  |
| ZNF281 | **IFNAR2** |  |  |
| NAIP | **IFNAR1** |  |  |
| PROX2 | **SON** |  |  |
| SLC5A3 | **SLC5A3** |  |  |
| HHLA1 | **RRP1B** |  |  |
| ANKRD23 | **TRAPPC10** |  |  |
| SETBP1 | **FP565260.7** |  |  |
| MACC1 | **DIP2A** |  |  |
| C3orf80 | **MAPK1** |  |  |
| CCDC82 | **PITPNB** |  |  |
| RAD51B | **EIF4ENIF1** |  |  |
| MME | **PRR14L** |  |  |
| SETD1A | **IL2RB** |  |  |
| JMY | **TOB2** |  |  |
| C1orf131 | **CERK** |  |  |
| PRMT9 | **BRD1** |  |  |
| ZNF674 | **TANGO2** |  |  |
| CTNNA3 | **HIC2** |  |  |
| SF3B3 | **KIAA1671** |  |  |
| GTPBP10 | **MTMR3** |  |  |
| PDE1A | **DEPDC5** |  |  |
| CLTCL1 | **TIMP3** |  |  |
| GALNT10 | **HMGXB4** |  |  |
| AKAP6 | **APOL6** |  |  |
| USP2 | **H1F0** |  |  |
| GAB1 | **MGAT3** |  |  |
| PLEKHJ1 | **TNRC6B** |  |  |
| PCNP | **PPARA** |  |  |
| ZNF182 | **ZBED4** |  |  |
| ATP7A | **PIM3** |  |  |
