# Supplementary material for: Circulating Long Non-Coding RNAs LINC00324 and LOC100507053 as Potential Liquid Biopsy Markers for Esophageal Squamous Cell Carcinoma: A Pilot Study
Source: Front Oncol. 2022 Feb 14;12:823953. doi: 10.3389/fonc.2022.823953 (PMC8882835; doi:10.3389/fonc.2022.823953)
Supplement: Supplementary file 6 [file Table_4.docx]

| RNAInter_ID  **Supplementary Table 4: LOC100507053-mRNA target prediction using various databases** | Interactor1 | Category1 | Species1 | Interactor2 | Category2 | Species2 | Score |
| --- | --- | --- | --- | --- | --- | --- | --- |
| RP08796816 | **LOC100507053** | **lncRNA** | **Homo sapiens** | **TARDBP** | **RBP** | **Homo sapiens** | **0.6668** |
| RP08613465 | **LOC100507053** | **lncRNA** | **Homo sapiens** | **PTBP1** | **RBP** | **Homo sapiens** | **0.6668** |
| RP33652257 | **LOC100507053** | **lncRNA** | **Homo sapiens** | **HNRNPA1** | **RBP** | **Homo sapiens** | **0.6166** |
| RP33593156 | **LOC100507053** | **lncRNA** | **Homo sapiens** | **U2AF2** | **RBP** | **Homo sapiens** | **0.6166** |
| RP08403780 | **LOC100507053** | **lncRNA** | **Homo sapiens** | **HNRNPC** | **RBP** | **Homo sapiens** | **0.6166** |
| RP08347376 | **LOC100507053** | **lncRNA** | **Homo sapiens** | **FUS** | **TF** | **Homo sapiens** | **0.6166** |
| RP08134524 | **LOC100507053** | **lncRNA** | **Homo sapiens** | **CSTF2** | **RBP** | **Homo sapiens** | **0.6166** |
| RR00853942 | **LOC100507053** | **lncRNA** | **Homo sapiens** | **ZNF443** | **mRNA** | **Homo sapiens** | **0.5117** |
| RR00565457 | **FHIT** | **mRNA** | **Homo sapiens** | **LOC100507053** | **lncRNA** | **Homo sapiens** | **0.5117** |
| RR00462899 | **DYNLL2** | **mRNA** | **Homo sapiens** | **LOC100507053** | **lncRNA** | **Homo sapiens** | **0.5117** |
| RP00734733 | **LOC100507053** | **lncRNA** | **Homo sapiens** | **STAT3** | **TF** | **Homo sapiens** | **0.5117** |
| RP33830076 | **LOC100507053** | **lncRNA** | **Homo sapiens** | **HNRNPU** | **RBP** | **Homo sapiens** | **0.5117** |
| RP33768441 | **LOC100507053** | **lncRNA** | **Homo sapiens** | **BCCIP** | **RBP** | **Homo sapiens** | **0.5117** |
| RP33737214 | **LOC100507053** | **lncRNA** | **Homo sapiens** | **DGCR8** | **RBP** | **Homo sapiens** | **0.5117** |
| RP33705668 | **LOC100507053** | **lncRNA** | **Homo sapiens** | **BUD13** | **RBP** | **Homo sapiens** | **0.5117** |
| RP33691988 | **LOC100507053** | **lncRNA** | **Homo sapiens** | **DICER1** | **RBP** | **Homo sapiens** | **0.5117** |
| RP33648746 | **LOC100507053** | **lncRNA** | **Homo sapiens** | **MSI1** | **RBP** | **Homo sapiens** | **0.5117** |
| RP33642754 | **LOC100507053** | **lncRNA** | **Homo sapiens** | **SAFB2** | **RBP** | **Homo sapiens** | **0.5117** |
| RP08950588 | **LOC100507053** | **lncRNA** | **Homo sapiens** | **ZC3H7B** | **RBP** | **Homo sapiens** | **0.5117** |
| RP08903048 | **LOC100507053** | **lncRNA** | **Homo sapiens** | **YTHDC1** | **RBP** | **Homo sapiens** | **0.5117** |
| RP08873844 | **LOC100507053** | **lncRNA** | **Homo sapiens** | **WDR33** | **RBP** | **Homo sapiens** | **0.5117** |
| RP08860650 | **LOC100507053** | **lncRNA** | **Homo sapiens** | **UPF1** | **RBP** | **Homo sapiens** | **0.5117** |
| RP08820719 | **LOC100507053** | **lncRNA** | **Homo sapiens** | **TIAL1** | **RBP** | **Homo sapiens** | **0.5117** |
| RP08805359 | **LOC100507053** | **lncRNA** | **Homo sapiens** | **TBRG4** | **RBP** | **Homo sapiens** | **0.5117** |
| RP08751289 | **LOC100507053** | **lncRNA** | **Homo sapiens** | **SSB** | **RBP** | **Homo sapiens** | **0.5117** |
| RP08710426 | **LOC100507053** | **lncRNA** | **Homo sapiens** | **SF3B1** | **RBP** | **Homo sapiens** | **0.5117** |
| RP08662077 | **LOC100507053** | **lncRNA** | **Homo sapiens** | **RBM15B** | **RBP** | **Homo sapiens** | **0.5117** |
| RP08628473 | **LOC100507053** | **lncRNA** | **Homo sapiens** | **QKI** | **RBP** | **Homo sapiens** | **0.5117** |
| RP08597528 | **LOC100507053** | **lncRNA** | **Homo sapiens** | **PRPF8** | **RBP** | **Homo sapiens** | **0.5117** |
| RP08577049 | **LOC100507053** | **lncRNA** | **Homo sapiens** | **NUDT21** | **RBP** | **Homo sapiens** | **0.5117** |
| RP08534444 | **LOC100507053** | **lncRNA** | **Homo sapiens** | **MOV10** | **RBP** | **Homo sapiens** | **0.5117** |
| RP08508809 | **LOC100507053** | **lncRNA** | **Homo sapiens** | **LIN28B** | **RBP** | **Homo sapiens** | **0.5117** |
| RP08426563 | **LOC100507053** | **lncRNA** | **Homo sapiens** | **HNRNPM** | **RBP** | **Homo sapiens** | **0.5117** |
| RP08316641 | **LOC100507053** | **lncRNA** | **Homo sapiens** | **FIP1L1** | **RBP** | **Homo sapiens** | **0.5117** |
| RP08277570 | **LOC100507053** | **lncRNA** | **Homo sapiens** | **ELAVL1** | **RBP** | **Homo sapiens** | **0.5117** |
| RP08247058 | **LOC100507053** | **lncRNA** | **Homo sapiens** | **EIF3G** | **RBP** | **Homo sapiens** | **0.5117** |
| RP08237162 | **LOC100507053** | **lncRNA** | **Homo sapiens** | **EIF3D** | **RBP** | **Homo sapiens** | **0.5117** |
| RP08230376 | **LOC100507053** | **lncRNA** | **Homo sapiens** | **EIF3B** | **RBP** | **Homo sapiens** | **0.5117** |
| RP08223551 | **LOC100507053** | **lncRNA** | **Homo sapiens** | **EIF3A** | **RBP** | **Homo sapiens** | **0.5117** |
| RP08150493 | **LOC100507053** | **lncRNA** | **Homo sapiens** | **CSTF2T** | **RBP** | **Homo sapiens** | **0.5117** |
| RP08107094 | **LOC100507053** | **lncRNA** | **Homo sapiens** | **CPSF6** | **RBP** | **Homo sapiens** | **0.5117** |
| RP08094130 | **LOC100507053** | **lncRNA** | **Homo sapiens** | **CPSF4** | **RBP** | **Homo sapiens** | **0.5117** |
| RP08064311 | **LOC100507053** | **lncRNA** | **Homo sapiens** | **CPSF1** | **RBP** | **Homo sapiens** | **0.5117** |
| RP08053058 | **LOC100507053** | **lncRNA** | **Homo sapiens** | **CNBP** | **RBP** | **Homo sapiens** | **0.5117** |
| RP08020355 | **LOC100507053** | **lncRNA** | **Homo sapiens** | **ATXN2** | **RBP** | **Homo sapiens** | **0.5117** |
| RP03087956 | **LOC100507053** | **lncRNA** | **Homo sapiens** | **HNF1B** | **TF** | **Homo sapiens** | **0.5117** |
| RP03069738 | **LOC100507053** | **lncRNA** | **Homo sapiens** | **PSIP1** | **TF** | **Homo sapiens** | **0.5117** |
| RP03060612 | **LOC100507053** | **lncRNA** | **Homo sapiens** | **DEK** | **TF** | **Homo sapiens** | **0.5117** |
| RP03040560 | **LOC100507053** | **lncRNA** | **Homo sapiens** | **ELK3** | **TF** | **Homo sapiens** | **0.5117** |
| RP03033414 | **LOC100507053** | **lncRNA** | **Homo sapiens** | **EGLN2** | **protein** | **Homo sapiens** | **0.5117** |
| RP03026341 | **LOC100507053** | **lncRNA** | **Homo sapiens** | **ARNT** | **TF** | **Homo sapiens** | **0.5117** |
| RP03017093 | **LOC100507053** | **lncRNA** | **Homo sapiens** | **HIF1A** | **TF** | **Homo sapiens** | **0.5117** |
| RP03003887 | **LOC100507053** | **lncRNA** | **Homo sapiens** | **KDM1B** | **protein** | **Homo sapiens** | **0.5117** |
| RP02993344 | **LOC100507053** | **lncRNA** | **Homo sapiens** | **RB1** | **TF** | **Homo sapiens** | **0.5117** |
| RP02984760 | **LOC100507053** | **lncRNA** | **Homo sapiens** | **EZH1** | **TF** | **Homo sapiens** | **0.5117** |
| RP02983046 | **LOC100507053** | **lncRNA** | **Homo sapiens** | **SUZ12** | **TF** | **Homo sapiens** | **0.5117** |
| RP02978761 | **LOC100507053** | **lncRNA** | **Homo sapiens** | **PDX1** | **TF** | **Homo sapiens** | **0.5117** |
| RP02970556 | **LOC100507053** | **lncRNA** | **Homo sapiens** | **CSNK2A1** | **TF** | **Homo sapiens** | **0.5117** |
| RP02958056 | **LOC100507053** | **lncRNA** | **Homo sapiens** | **CTBP1** | **TF** | **Homo sapiens** | **0.5117** |
| RP02955152 | **LOC100507053** | **lncRNA** | **Homo sapiens** | **NOTCH1** | **TF** | **Homo sapiens** | **0.5117** |
| RP02948310 | **LOC100507053** | **lncRNA** | **Homo sapiens** | **ERCC6** | **protein** | **Homo sapiens** | **0.5117** |
| RP02941655 | **LOC100507053** | **lncRNA** | **Homo sapiens** | **ZNF750** | **TF** | **Homo sapiens** | **0.5117** |
| RP02930871 | **LOC100507053** | **lncRNA** | **Homo sapiens** | **IRF4** | **TF** | **Homo sapiens** | **0.5117** |
| RP02874115 | **LOC100507053** | **lncRNA** | **Homo sapiens** | **TEAD1** | **TF** | **Homo sapiens** | **0.5117** |
| RP02865956 | **LOC100507053** | **lncRNA** | **Homo sapiens** | **TCF7** | **TF** | **Homo sapiens** | **0.5117** |
| RP02844596 | **LOC100507053** | **lncRNA** | **Homo sapiens** | **CDK6** | **TF** | **Homo sapiens** | **0.5117** |
| RP02823054 | **LOC100507053** | **lncRNA** | **Homo sapiens** | **GATA4** | **TF** | **Homo sapiens** | **0.5117** |
| RP02805439 | **LOC100507053** | **lncRNA** | **Homo sapiens** | **ZNF83** | **TF** | **Homo sapiens** | **0.5117** |
| RP02792807 | **LOC100507053** | **lncRNA** | **Homo sapiens** | **ZNF12** | **TF** | **Homo sapiens** | **0.5117** |
| RP02761978 | **LOC100507053** | **lncRNA** | **Homo sapiens** | **ZBTB10** | **TF** | **Homo sapiens** | **0.5117** |
| RP02758443 | **LOC100507053** | **lncRNA** | **Homo sapiens** | **STAT6** | **TF** | **Homo sapiens** | **0.5117** |
| RP02756170 | **LOC100507053** | **lncRNA** | **Homo sapiens** | **STAT2** | **TF** | **Homo sapiens** | **0.5117** |
| RP02748190 | **LOC100507053** | **lncRNA** | **Homo sapiens** | **RUNX1** | **TF** | **Homo sapiens** | **0.5117** |
| RP02746139 | **LOC100507053** | **lncRNA** | **Homo sapiens** | **OVOL2** | **TF** | **Homo sapiens** | **0.5117** |
| RP02741153 | **LOC100507053** | **lncRNA** | **Homo sapiens** | **NPAT** | **TF** | **Homo sapiens** | **0.5117** |
| RP02738594 | **LOC100507053** | **lncRNA** | **Homo sapiens** | **NFYC** | **TF** | **Homo sapiens** | **0.5117** |
| RP02730691 | **LOC100507053** | **lncRNA** | **Homo sapiens** | **HOXC8** | **TF** | **Homo sapiens** | **0.5117** |
| RP02728342 | **LOC100507053** | **lncRNA** | **Homo sapiens** | **GATAD1** | **TF** | **Homo sapiens** | **0.5117** |
| RP02724585 | **LOC100507053** | **lncRNA** | **Homo sapiens** | **FOXP1** | **TF** | **Homo sapiens** | **0.5117** |
| RP02720609 | **LOC100507053** | **lncRNA** | **Homo sapiens** | **ELF2** | **TF** | **Homo sapiens** | **0.5117** |
| RP02717233 | **LOC100507053** | **lncRNA** | **Homo sapiens** | **BARX1** | **TF** | **Homo sapiens** | **0.5117** |
| RP02714568 | **LOC100507053** | **lncRNA** | **Homo sapiens** | **ARNTL** | **TF** | **Homo sapiens** | **0.5117** |
| RP02713052 | **LOC100507053** | **lncRNA** | **Homo sapiens** | **SOX9** | **TF** | **Homo sapiens** | **0.5117** |
| RP02704424 | **LOC100507053** | **lncRNA** | **Homo sapiens** | **HOXA6** | **TF** | **Homo sapiens** | **0.5117** |
| RP02700930 | **LOC100507053** | **lncRNA** | **Homo sapiens** | **HOXA5** | **TF** | **Homo sapiens** | **0.5117** |
| RP02697473 | **LOC100507053** | **lncRNA** | **Homo sapiens** | **HOXA4** | **TF** | **Homo sapiens** | **0.5117** |
| RP02695723 | **LOC100507053** | **lncRNA** | **Homo sapiens** | **HEYL** | **TF** | **Homo sapiens** | **0.5117** |
| RP02694713 | **LOC100507053** | **lncRNA** | **Homo sapiens** | **GLI2** | **TF** | **Homo sapiens** | **0.5117** |
| RP02693846 | **LOC100507053** | **lncRNA** | **Homo sapiens** | **ETV5** | **TF** | **Homo sapiens** | **0.5117** |
| RP02690332 | **LOC100507053** | **lncRNA** | **Homo sapiens** | **E2F3** | **TF** | **Homo sapiens** | **0.5117** |
| RP02670073 | **LOC100507053** | **lncRNA** | **Homo sapiens** | **MYOD1** | **TF** | **Homo sapiens** | **0.5117** |
| RP02663918 | **LOC100507053** | **lncRNA** | **Homo sapiens** | **VEZF1** | **TF** | **Homo sapiens** | **0.5117** |
| RP02656083 | **LOC100507053** | **lncRNA** | **Homo sapiens** | **TEAD2** | **TF** | **Homo sapiens** | **0.5117** |
| RP02641375 | **LOC100507053** | **lncRNA** | **Homo sapiens** | **RBCK1** | **protein** | **Homo sapiens** | **0.5117** |
| RP02631449 | **LOC100507053** | **lncRNA** | **Homo sapiens** | **PATZ1** | **TF** | **Homo sapiens** | **0.5117** |
| RP02619782 | **LOC100507053** | **lncRNA** | **Homo sapiens** | **NFKB2** | **TF** | **Homo sapiens** | **0.5117** |
| RP02615143 | **LOC100507053** | **lncRNA** | **Homo sapiens** | **MXD1** | **TF** | **Homo sapiens** | **0.5117** |
| RP02608636 | **LOC100507053** | **lncRNA** | **Homo sapiens** | **MNT** | **TF** | **Homo sapiens** | **0.5117** |
| RP02602017 | **LOC100507053** | **lncRNA** | **Homo sapiens** | **LYL1** | **TF** | **Homo sapiens** | **0.5117** |
| RP02590031 | **LOC100507053** | **lncRNA** | **Homo sapiens** | **HOXA2** | **TF** | **Homo sapiens** | **0.5117** |
| RP02586363 | **LOC100507053** | **lncRNA** | **Homo sapiens** | **HOXA13** | **TF** | **Homo sapiens** | **0.5117** |
| RP02580063 | **LOC100507053** | **lncRNA** | **Homo sapiens** | **GLIS1** | **TF** | **Homo sapiens** | **0.5117** |
| RP02576047 | **LOC100507053** | **lncRNA** | **Homo sapiens** | **GLI3** | **TF** | **Homo sapiens** | **0.5117** |
| RP02570474 | **LOC100507053** | **lncRNA** | **Homo sapiens** | **FOXG1** | **TF** | **Homo sapiens** | **0.5117** |
| RP02566085 | **LOC100507053** | **lncRNA** | **Homo sapiens** | **FOXD2** | **TF** | **Homo sapiens** | **0.5117** |
| RP02560927 | **LOC100507053** | **lncRNA** | **Homo sapiens** | **FEV** | **TF** | **Homo sapiens** | **0.5117** |
| RP02556088 | **LOC100507053** | **lncRNA** | **Homo sapiens** | **MECOM** | **TF** | **Homo sapiens** | **0.5117** |
| RP02548290 | **LOC100507053** | **lncRNA** | **Homo sapiens** | **ETS2** | **TF** | **Homo sapiens** | **0.5117** |
| RP02535833 | **LOC100507053** | **lncRNA** | **Homo sapiens** | **E2F8** | **TF** | **Homo sapiens** | **0.5117** |
| RP02525595 | **LOC100507053** | **lncRNA** | **Homo sapiens** | **DLX1** | **TF** | **Homo sapiens** | **0.5117** |
| RP02521706 | **LOC100507053** | **lncRNA** | **Homo sapiens** | **CREB3L4** | **TF** | **Homo sapiens** | **0.5117** |
| RP02516388 | **LOC100507053** | **lncRNA** | **Homo sapiens** | **CLOCK** | **TF** | **Homo sapiens** | **0.5117** |
| RP02509918 | **LOC100507053** | **lncRNA** | **Homo sapiens** | **BARHL1** | **TF** | **Homo sapiens** | **0.5117** |
| RP02499092 | **LOC100507053** | **lncRNA** | **Homo sapiens** | **ADNP** | **TF** | **Homo sapiens** | **0.5117** |
| RP02487054 | **LOC100507053** | **lncRNA** | **Homo sapiens** | **ZNF250** | **TF** | **Homo sapiens** | **0.5117** |
| RP02479730 | **LOC100507053** | **lncRNA** | **Homo sapiens** | **ZBED4** | **TF** | **Homo sapiens** | **0.5117** |
| RP02471002 | **LOC100507053** | **lncRNA** | **Homo sapiens** | **TFDP1** | **TF** | **Homo sapiens** | **0.5117** |
| RP02464339 | **LOC100507053** | **lncRNA** | **Homo sapiens** | **SP3** | **TF** | **Homo sapiens** | **0.5117** |
| RP02453287 | **LOC100507053** | **lncRNA** | **Homo sapiens** | **SMC1A** | **TF** | **Homo sapiens** | **0.5117** |
| RP02439406 | **LOC100507053** | **lncRNA** | **Homo sapiens** | **RFX1** | **TF** | **Homo sapiens** | **0.5117** |
| RP02426783 | **LOC100507053** | **lncRNA** | **Homo sapiens** | **NIPBL** | **TF** | **Homo sapiens** | **0.5117** |
| RP02419644 | **LOC100507053** | **lncRNA** | **Homo sapiens** | **NFE2L2** | **TF** | **Homo sapiens** | **0.5117** |
| RP02417809 | **LOC100507053** | **lncRNA** | **Homo sapiens** | **NFATC3** | **TF** | **Homo sapiens** | **0.5117** |
| RP02413697 | **LOC100507053** | **lncRNA** | **Homo sapiens** | **NFAT5** | **TF** | **Homo sapiens** | **0.5117** |
| RP02403547 | **LOC100507053** | **lncRNA** | **Homo sapiens** | **MYB** | **TF** | **Homo sapiens** | **0.5117** |
| RP02396538 | **LOC100507053** | **lncRNA** | **Homo sapiens** | **MED1** | **TF** | **Homo sapiens** | **0.5117** |
| RP02391740 | **LOC100507053** | **lncRNA** | **Homo sapiens** | **MED12** | **TF** | **Homo sapiens** | **0.5117** |
| RP02387969 | **LOC100507053** | **lncRNA** | **Homo sapiens** | **LHX2** | **TF** | **Homo sapiens** | **0.5117** |
| RP02380943 | **LOC100507053** | **lncRNA** | **Homo sapiens** | **KLF5** | **TF** | **Homo sapiens** | **0.5117** |
| RP02368217 | **LOC100507053** | **lncRNA** | **Homo sapiens** | **HOXA7** | **TF** | **Homo sapiens** | **0.5117** |
| RP02352279 | **LOC100507053** | **lncRNA** | **Homo sapiens** | **HINFP** | **TF** | **Homo sapiens** | **0.5117** |
| RP02342632 | **LOC100507053** | **lncRNA** | **Homo sapiens** | **HES1** | **TF** | **Homo sapiens** | **0.5117** |
| RP02333255 | **LOC100507053** | **lncRNA** | **Homo sapiens** | **GMEB2** | **TF** | **Homo sapiens** | **0.5117** |
| RP02328187 | **LOC100507053** | **lncRNA** | **Homo sapiens** | **GMEB1** | **TF** | **Homo sapiens** | **0.5117** |
| RP02325785 | **LOC100507053** | **lncRNA** | **Homo sapiens** | **GLIS3** | **TF** | **Homo sapiens** | **0.5117** |
| RP02311560 | **LOC100507053** | **lncRNA** | **Homo sapiens** | **ERF** | **TF** | **Homo sapiens** | **0.5117** |
| RP02306983 | **LOC100507053** | **lncRNA** | **Homo sapiens** | **EHF** | **TF** | **Homo sapiens** | **0.5117** |
| RP02267545 | **LOC100507053** | **lncRNA** | **Homo sapiens** | **EBNA-LP** | **protein** | **Homo sapiens** | **0.5117** |
| RP02256490 | **LOC100507053** | **lncRNA** | **Homo sapiens** | **SPDEF** | **TF** | **Homo sapiens** | **0.5117** |
| RP02225713 | **LOC100507053** | **lncRNA** | **Homo sapiens** | **BRD1** | **TF** | **Homo sapiens** | **0.5117** |
| RP02219240 | **LOC100507053** | **lncRNA** | **Homo sapiens** | **ETV1** | **TF** | **Homo sapiens** | **0.5117** |
| RP02191774 | **LOC100507053** | **lncRNA** | **Homo sapiens** | **UBN1** | **protein** | **Homo sapiens** | **0.5117** |
| RP02187696 | **LOC100507053** | **lncRNA** | **Homo sapiens** | **HIRA** | **TF** | **Homo sapiens** | **0.5117** |
| RP02181166 | **LOC100507053** | **lncRNA** | **Homo sapiens** | **ASF1A** | **TF** | **Homo sapiens** | **0.5117** |
| RP02174709 | **LOC100507053** | **lncRNA** | **Homo sapiens** | **NELFA** | **TF** | **Homo sapiens** | **0.5117** |
| RP02163462 | **LOC100507053** | **lncRNA** | **Homo sapiens** | **ERCC3** | **TF** | **Homo sapiens** | **0.5117** |
| RP02155551 | **LOC100507053** | **lncRNA** | **Homo sapiens** | **MBD3** | **TF** | **Homo sapiens** | **0.5117** |
| RP02124713 | **LOC100507053** | **lncRNA** | **Homo sapiens** | **KLF1** | **TF** | **Homo sapiens** | **0.5117** |
| RP02119646 | **LOC100507053** | **lncRNA** | **Homo sapiens** | **GATA1** | **TF** | **Homo sapiens** | **0.5117** |
| RP02115233 | **LOC100507053** | **lncRNA** | **Homo sapiens** | **TAF3** | **TF** | **Homo sapiens** | **0.5117** |
| RP02096098 | **LOC100507053** | **lncRNA** | **Homo sapiens** | **ESR2** | **TF** | **Homo sapiens** | **0.5117** |
| RP02057611 | **LOC100507053** | **lncRNA** | **Homo sapiens** | **PGR** | **TF** | **Homo sapiens** | **0.5117** |
| RP02049661 | **LOC100507053** | **lncRNA** | **Homo sapiens** | **AGO1** | **TF** | **Homo sapiens** | **0.5117** |
| RP02043753 | **LOC100507053** | **lncRNA** | **Homo sapiens** | **E2F7** | **TF** | **Homo sapiens** | **0.5117** |
| RP02030565 | **LOC100507053** | **lncRNA** | **Homo sapiens** | **NKX2-1** | **TF** | **Homo sapiens** | **0.5117** |
| RP02008109 | **LOC100507053** | **lncRNA** | **Homo sapiens** | **AFF1** | **TF** | **Homo sapiens** | **0.5117** |
| RP02007111 | **LOC100507053** | **lncRNA** | **Homo sapiens** | **KMT2A** | **TF** | **Homo sapiens** | **0.5117** |
| RP02002234 | **LOC100507053** | **lncRNA** | **Homo sapiens** | **CDK8** | **TF** | **Homo sapiens** | **0.5117** |
| RP01999226 | **LOC100507053** | **lncRNA** | **Homo sapiens** | **NFE2** | **TF** | **Homo sapiens** | **0.5117** |
| RP01990977 | **LOC100507053** | **lncRNA** | **Homo sapiens** | **KLF4** | **TF** | **Homo sapiens** | **0.5117** |
| RP01986042 | **LOC100507053** | **lncRNA** | **Homo sapiens** | **SOX2** | **TF** | **Homo sapiens** | **0.5117** |
| RP01972605 | **LOC100507053** | **lncRNA** | **Homo sapiens** | **TBL1X** | **protein** | **Homo sapiens** | **0.5117** |
| RP01919455 | **LOC100507053** | **lncRNA** | **Homo sapiens** | **BCOR** | **TF** | **Homo sapiens** | **0.5117** |
| RP01914051 | **LOC100507053** | **lncRNA** | **Homo sapiens** | **BCL6** | **TF** | **Homo sapiens** | **0.5117** |
| RP01901182 | **LOC100507053** | **lncRNA** | **Homo sapiens** | **FOXH1** | **TF** | **Homo sapiens** | **0.5117** |
| RP01861131 | **LOC100507053** | **lncRNA** | **Homo sapiens** | **SMARCA4** | **TF** | **Homo sapiens** | **0.5117** |
| RP01848008 | **LOC100507053** | **lncRNA** | **Homo sapiens** | **EOMES** | **TF** | **Homo sapiens** | **0.5117** |
| RP01834948 | **LOC100507053** | **lncRNA** | **Homo sapiens** | **SMARCC1** | **TF** | **Homo sapiens** | **0.5117** |
| RP01828205 | **LOC100507053** | **lncRNA** | **Homo sapiens** | **SMARCB1** | **TF** | **Homo sapiens** | **0.5117** |
| RP01810363 | **LOC100507053** | **lncRNA** | **Homo sapiens** | **HNF4A** | **TF** | **Homo sapiens** | **0.5117** |
| RP01799133 | **LOC100507053** | **lncRNA** | **Homo sapiens** | **GATA6** | **TF** | **Homo sapiens** | **0.5117** |
| RP01785448 | **LOC100507053** | **lncRNA** | **Homo sapiens** | **CDX2** | **TF** | **Homo sapiens** | **0.5117** |
| RP01762427 | **LOC100507053** | **lncRNA** | **Homo sapiens** | **PPARG** | **TF** | **Homo sapiens** | **0.5117** |
| RP01749632 | **LOC100507053** | **lncRNA** | **Homo sapiens** | **NFKB1** | **TF** | **Homo sapiens** | **0.5117** |
| RP01736737 | **LOC100507053** | **lncRNA** | **Homo sapiens** | **TP63** | **TF** | **Homo sapiens** | **0.5117** |
| RP01712347 | **LOC100507053** | **lncRNA** | **Homo sapiens** | **ERG** | **TF** | **Homo sapiens** | **0.5117** |
| RP01701933 | **LOC100507053** | **lncRNA** | **Homo sapiens** | **KLF9** | **TF** | **Homo sapiens** | **0.5117** |
| RP01687720 | **LOC100507053** | **lncRNA** | **Homo sapiens** | **ZNF552** | **TF** | **Homo sapiens** | **0.5117** |
| RP01683430 | **LOC100507053** | **lncRNA** | **Homo sapiens** | **AR** | **TF** | **Homo sapiens** | **0.5117** |
| RP01670823 | **LOC100507053** | **lncRNA** | **Homo sapiens** | **STAG1** | **TF** | **Homo sapiens** | **0.5117** |
| RP01666425 | **LOC100507053** | **lncRNA** | **Homo sapiens** | **MITF** | **TF** | **Homo sapiens** | **0.5117** |
| RP01647455 | **LOC100507053** | **lncRNA** | **Homo sapiens** | **STAT4** | **TF** | **Homo sapiens** | **0.5117** |
| RP01644078 | **LOC100507053** | **lncRNA** | **Homo sapiens** | **IRF5** | **TF** | **Homo sapiens** | **0.5117** |
| RP01635326 | **LOC100507053** | **lncRNA** | **Homo sapiens** | **TP53** | **TF** | **Homo sapiens** | **0.5117** |
| RP01612103 | **LOC100507053** | **lncRNA** | **Homo sapiens** | **CEBPA** | **TF** | **Homo sapiens** | **0.5117** |
| RP01604630 | **LOC100507053** | **lncRNA** | **Homo sapiens** | **SMAD2** | **TF** | **Homo sapiens** | **0.5117** |
| RP01604629 | **LOC100507053** | **lncRNA** | **Homo sapiens** | **SMAD3** | **TF** | **Homo sapiens** | **0.5117** |
| RP01600431 | **LOC100507053** | **lncRNA** | **Homo sapiens** | **TP73** | **TF** | **Homo sapiens** | **0.5117** |
| RP01590385 | **LOC100507053** | **lncRNA** | **Homo sapiens** | **HCFC1** | **TF** | **Homo sapiens** | **0.5117** |
| RP01574824 | **LOC100507053** | **lncRNA** | **Homo sapiens** | **CUX1** | **TF** | **Homo sapiens** | **0.5117** |
| RP01566644 | **LOC100507053** | **lncRNA** | **Homo sapiens** | **ZNF384** | **TF** | **Homo sapiens** | **0.5117** |
| RP01558353 | **LOC100507053** | **lncRNA** | **Homo sapiens** | **ZMIZ1** | **TF** | **Homo sapiens** | **0.5117** |
| RP01551113 | **LOC100507053** | **lncRNA** | **Homo sapiens** | **ZC3H11A** | **TF** | **Homo sapiens** | **0.5117** |
| RP01541333 | **LOC100507053** | **lncRNA** | **Homo sapiens** | **PBX3** | **TF** | **Homo sapiens** | **0.5117** |
| RP01524416 | **LOC100507053** | **lncRNA** | **Homo sapiens** | **FOSL1** | **TF** | **Homo sapiens** | **0.5117** |
| RP01507331 | **LOC100507053** | **lncRNA** | **Homo sapiens** | **NSD2** | **TF** | **Homo sapiens** | **0.5117** |
| RP01497582 | **LOC100507053** | **lncRNA** | **Homo sapiens** | **KDM4A** | **TF** | **Homo sapiens** | **0.5117** |
| RP01487729 | **LOC100507053** | **lncRNA** | **Homo sapiens** | **CHD7** | **TF** | **Homo sapiens** | **0.5117** |
| RP01468122 | **LOC100507053** | **lncRNA** | **Homo sapiens** | **SIRT6** | **TF** | **Homo sapiens** | **0.5117** |
| RP01447247 | **LOC100507053** | **lncRNA** | **Homo sapiens** | **NCOR1** | **TF** | **Homo sapiens** | **0.5117** |
| RP01440094 | **LOC100507053** | **lncRNA** | **Homo sapiens** | **KDM1A** | **TF** | **Homo sapiens** | **0.5117** |
| RP01417754 | **LOC100507053** | **lncRNA** | **Homo sapiens** | **CREBBP** | **TF** | **Homo sapiens** | **0.5117** |
| RP01399775 | **LOC100507053** | **lncRNA** | **Homo sapiens** | **CHD4** | **TF** | **Homo sapiens** | **0.5117** |
| RP01388168 | **LOC100507053** | **lncRNA** | **Homo sapiens** | **HDAC6** | **TF** | **Homo sapiens** | **0.5117** |
| RP01378357 | **LOC100507053** | **lncRNA** | **Homo sapiens** | **ATF1** | **TF** | **Homo sapiens** | **0.5117** |
| RP01362716 | **LOC100507053** | **lncRNA** | **Homo sapiens** | **ARID3A** | **TF** | **Homo sapiens** | **0.5117** |
| RP01342678 | **LOC100507053** | **lncRNA** | **Homo sapiens** | **MAZ** | **TF** | **Homo sapiens** | **0.5117** |
| RP01335583 | **LOC100507053** | **lncRNA** | **Homo sapiens** | **ELK1** | **TF** | **Homo sapiens** | **0.5117** |
| RP01325610 | **LOC100507053** | **lncRNA** | **Homo sapiens** | **TBL1XR1** | **TF** | **Homo sapiens** | **0.5117** |
| RP01300716 | **LOC100507053** | **lncRNA** | **Homo sapiens** | **E2F4** | **TF** | **Homo sapiens** | **0.5117** |
| RP01282829 | **LOC100507053** | **lncRNA** | **Homo sapiens** | **RCOR1** | **TF** | **Homo sapiens** | **0.5117** |
| RP01258444 | **LOC100507053** | **lncRNA** | **Homo sapiens** | **FOXM1** | **TF** | **Homo sapiens** | **0.5117** |
| RP01247392 | **LOC100507053** | **lncRNA** | **Homo sapiens** | **CBX3** | **TF** | **Homo sapiens** | **0.5117** |
| RP01239840 | **LOC100507053** | **lncRNA** | **Homo sapiens** | **NR2F2** | **TF** | **Homo sapiens** | **0.5117** |
| RP01230355 | **LOC100507053** | **lncRNA** | **Homo sapiens** | **MYBL2** | **TF** | **Homo sapiens** | **0.5117** |
| RP01217755 | **LOC100507053** | **lncRNA** | **Homo sapiens** | **TEAD4** | **TF** | **Homo sapiens** | **0.5117** |
| RP01209038 | **LOC100507053** | **lncRNA** | **Homo sapiens** | **RUNX3** | **TF** | **Homo sapiens** | **0.5117** |
| RP01200190 | **LOC100507053** | **lncRNA** | **Homo sapiens** | **MTA3** | **TF** | **Homo sapiens** | **0.5117** |
| RP01191752 | **LOC100507053** | **lncRNA** | **Homo sapiens** | **CREB1** | **TF** | **Homo sapiens** | **0.5117** |
| RP01184673 | **LOC100507053** | **lncRNA** | **Homo sapiens** | **STAT5A** | **TF** | **Homo sapiens** | **0.5117** |
| RP01177760 | **LOC100507053** | **lncRNA** | **Homo sapiens** | **NFIC** | **TF** | **Homo sapiens** | **0.5117** |
| RP01172606 | **LOC100507053** | **lncRNA** | **Homo sapiens** | **MBD4** | **TF** | **Homo sapiens** | **0.5117** |
| RP01169746 | **LOC100507053** | **lncRNA** | **Homo sapiens** | **SP4** | **TF** | **Homo sapiens** | **0.5117** |
| RP01164649 | **LOC100507053** | **lncRNA** | **Homo sapiens** | **TCF3** | **TF** | **Homo sapiens** | **0.5117** |
| RP01148886 | **LOC100507053** | **lncRNA** | **Homo sapiens** | **PML** | **TF** | **Homo sapiens** | **0.5117** |
| RP01146739 | **LOC100507053** | **lncRNA** | **Homo sapiens** | **NFATC1** | **TF** | **Homo sapiens** | **0.5117** |
| RP01137395 | **LOC100507053** | **lncRNA** | **Homo sapiens** | **ATF2** | **TF** | **Homo sapiens** | **0.5117** |
| RP01131429 | **LOC100507053** | **lncRNA** | **Homo sapiens** | **CEBPD** | **TF** | **Homo sapiens** | **0.5117** |
| RP01107926 | **LOC100507053** | **lncRNA** | **Homo sapiens** | **KDM5A** | **TF** | **Homo sapiens** | **0.5117** |
| RP01092540 | **LOC100507053** | **lncRNA** | **Homo sapiens** | **SAP30** | **TF** | **Homo sapiens** | **0.5117** |
| RP01080623 | **LOC100507053** | **lncRNA** | **Homo sapiens** | **PHF8** | **TF** | **Homo sapiens** | **0.5117** |
| RP01064657 | **LOC100507053** | **lncRNA** | **Homo sapiens** | **HDAC1** | **TF** | **Homo sapiens** | **0.5117** |
| RP01054630 | **LOC100507053** | **lncRNA** | **Homo sapiens** | **EZH2** | **TF** | **Homo sapiens** | **0.5117** |
| RP01042825 | **LOC100507053** | **lncRNA** | **Homo sapiens** | **CHD1** | **TF** | **Homo sapiens** | **0.5117** |
| RP01029571 | **LOC100507053** | **lncRNA** | **Homo sapiens** | **RBBP5** | **TF** | **Homo sapiens** | **0.5117** |
| RP01016389 | **LOC100507053** | **lncRNA** | **Homo sapiens** | **KDM5B** | **TF** | **Homo sapiens** | **0.5117** |
| RP01010635 | **LOC100507053** | **lncRNA** | **Homo sapiens** | **TCF7L2** | **TF** | **Homo sapiens** | **0.5117** |
| RP00994026 | **LOC100507053** | **lncRNA** | **Homo sapiens** | **GATA3** | **TF** | **Homo sapiens** | **0.5117** |
| RP00969984 | **LOC100507053** | **lncRNA** | **Homo sapiens** | **IRF1** | **TF** | **Homo sapiens** | **0.5117** |
| RP00962670 | **LOC100507053** | **lncRNA** | **Homo sapiens** | **CCNT2** | **TF** | **Homo sapiens** | **0.5117** |
| RP00954996 | **LOC100507053** | **lncRNA** | **Homo sapiens** | **HMGN3** | **TF** | **Homo sapiens** | **0.5117** |
| RP00945202 | **LOC100507053** | **lncRNA** | **Homo sapiens** | **BHLHE40** | **TF** | **Homo sapiens** | **0.5117** |
| RP00933235 | **LOC100507053** | **lncRNA** | **Homo sapiens** | **ZNF143** | **TF** | **Homo sapiens** | **0.5117** |
| RP00925199 | **LOC100507053** | **lncRNA** | **Homo sapiens** | **STAT1** | **TF** | **Homo sapiens** | **0.5117** |
| RP00905980 | **LOC100507053** | **lncRNA** | **Homo sapiens** | **MAFK** | **TF** | **Homo sapiens** | **0.5117** |
| RP00893414 | **LOC100507053** | **lncRNA** | **Homo sapiens** | **MAFF** | **TF** | **Homo sapiens** | **0.5117** |
| RP00886887 | **LOC100507053** | **lncRNA** | **Homo sapiens** | **SMC3** | **TF** | **Homo sapiens** | **0.5117** |
| RP00861449 | **LOC100507053** | **lncRNA** | **Homo sapiens** | **MXI1** | **TF** | **Homo sapiens** | **0.5117** |
| RP00848242 | **LOC100507053** | **lncRNA** | **Homo sapiens** | **CHD2** | **TF** | **Homo sapiens** | **0.5117** |
| RP00830961 | **LOC100507053** | **lncRNA** | **Homo sapiens** | **GTF2F1** | **TF** | **Homo sapiens** | **0.5117** |
| RP00816429 | **LOC100507053** | **lncRNA** | **Homo sapiens** | **BRCA1** | **TF** | **Homo sapiens** | **0.5117** |
| RP00811978 | **LOC100507053** | **lncRNA** | **Homo sapiens** | **USF2** | **TF** | **Homo sapiens** | **0.5117** |
| RP00807130 | **LOC100507053** | **lncRNA** | **Homo sapiens** | **RFX5** | **TF** | **Homo sapiens** | **0.5117** |
| RP00803137 | **LOC100507053** | **lncRNA** | **Homo sapiens** | **IRF3** | **TF** | **Homo sapiens** | **0.5117** |
| RP00790993 | **LOC100507053** | **lncRNA** | **Homo sapiens** | **RAD21** | **TF** | **Homo sapiens** | **0.5117** |
| RP00783113 | **LOC100507053** | **lncRNA** | **Homo sapiens** | **NRF1** | **TF** | **Homo sapiens** | **0.5117** |
| RP00780261 | **LOC100507053** | **lncRNA** | **Homo sapiens** | **JUN** | **TF** | **Homo sapiens** | **0.5117** |
| RP00773530 | **LOC100507053** | **lncRNA** | **Homo sapiens** | **TBP** | **TF** | **Homo sapiens** | **0.5117** |
| RP00764463 | **LOC100507053** | **lncRNA** | **Homo sapiens** | **WRNIP1** | **TF** | **Homo sapiens** | **0.5117** |
| RP00756536 | **LOC100507053** | **lncRNA** | **Homo sapiens** | **ZNF263** | **TF** | **Homo sapiens** | **0.5117** |
| RP00745928 | **LOC100507053** | **lncRNA** | **Homo sapiens** | **FOS** | **TF** | **Homo sapiens** | **0.5117** |
| RP33873539 | **LOC100507053** | **lncRNA** | **Homo sapiens** | **AUH** | **RBP** | **Homo sapiens** | **0.5117** |
| RP00716781 | **LOC100507053** | **lncRNA** | **Homo sapiens** | **SETDB1** | **TF** | **Homo sapiens** | **0.5117** |
| RP00708449 | **LOC100507053** | **lncRNA** | **Homo sapiens** | **ELK4** | **TF** | **Homo sapiens** | **0.5117** |
| RP00690323 | **LOC100507053** | **lncRNA** | **Homo sapiens** | **SP2** | **TF** | **Homo sapiens** | **0.5117** |
| RP00675695 | **LOC100507053** | **lncRNA** | **Homo sapiens** | **FOXA2** | **TF** | **Homo sapiens** | **0.5117** |
| RP00667412 | **LOC100507053** | **lncRNA** | **Homo sapiens** | **EGR1** | **TF** | **Homo sapiens** | **0.5117** |
| RP00642415 | **LOC100507053** | **lncRNA** | **Homo sapiens** | **HDAC2** | **TF** | **Homo sapiens** | **0.5117** |
| RP00641945 | **LOC100507053** | **lncRNA** | **Homo sapiens** | **USF1** | **TF** | **Homo sapiens** | **0.5117** |
| RP00635776 | **LOC100507053** | **lncRNA** | **Homo sapiens** | **ZBTB7A** | **TF** | **Homo sapiens** | **0.5117** |
| RP00620711 | **LOC100507053** | **lncRNA** | **Homo sapiens** | **ELF1** | **TF** | **Homo sapiens** | **0.5117** |
| RP00613705 | **LOC100507053** | **lncRNA** | **Homo sapiens** | **EP300** | **TF** | **Homo sapiens** | **0.5117** |
| RP00609372 | **LOC100507053** | **lncRNA** | **Homo sapiens** | **TAF7** | **TF** | **Homo sapiens** | **0.5117** |
| RP00603670 | **LOC100507053** | **lncRNA** | **Homo sapiens** | **MAX** | **TF** | **Homo sapiens** | **0.5117** |
| RP00590812 | **LOC100507053** | **lncRNA** | **Homo sapiens** | **FOXA1** | **TF** | **Homo sapiens** | **0.5117** |
| RP00570918 | **LOC100507053** | **lncRNA** | **Homo sapiens** | **ETS1** | **TF** | **Homo sapiens** | **0.5117** |
| RP00564129 | **LOC100507053** | **lncRNA** | **Homo sapiens** | **BCL3** | **TF** | **Homo sapiens** | **0.5117** |
| RP00553049 | **LOC100507053** | **lncRNA** | **Homo sapiens** | **MEF2A** | **TF** | **Homo sapiens** | **0.5117** |
| RP00544037 | **LOC100507053** | **lncRNA** | **Homo sapiens** | **BCLAF1** | **TF** | **Homo sapiens** | **0.5117** |
| RP00534896 | **LOC100507053** | **lncRNA** | **Homo sapiens** | **REST** | **TF** | **Homo sapiens** | **0.5117** |
| RP00525824 | **LOC100507053** | **lncRNA** | **Homo sapiens** | **ESR1** | **TF** | **Homo sapiens** | **0.5117** |
| RP00498211 | **LOC100507053** | **lncRNA** | **Homo sapiens** | **FOSL2** | **TF** | **Homo sapiens** | **0.5117** |
| RP00484489 | **LOC100507053** | **lncRNA** | **Homo sapiens** | **NR3C1** | **TF** | **Homo sapiens** | **0.5117** |
| RP00477051 | **LOC100507053** | **lncRNA** | **Homo sapiens** | **PAX5** | **TF** | **Homo sapiens** | **0.5117** |
| RP00460308 | **LOC100507053** | **lncRNA** | **Homo sapiens** | **TCF12** | **TF** | **Homo sapiens** | **0.5117** |
| RP00449178 | **LOC100507053** | **lncRNA** | **Homo sapiens** | **SPI1** | **TF** | **Homo sapiens** | **0.5117** |
| RP00444012 | **LOC100507053** | **lncRNA** | **Homo sapiens** | **HEY1** | **TF** | **Homo sapiens** | **0.5117** |
| RP00436010 | **LOC100507053** | **lncRNA** | **Homo sapiens** | **EBF1** | **TF** | **Homo sapiens** | **0.5117** |
| RP00422224 | **LOC100507053** | **lncRNA** | **Homo sapiens** | **TAF1** | **TF** | **Homo sapiens** | **0.5117** |
| RP00400325 | **LOC100507053** | **lncRNA** | **Homo sapiens** | **POU2F2** | **TF** | **Homo sapiens** | **0.5117** |
| RP00390130 | **LOC100507053** | **lncRNA** | **Homo sapiens** | **SIN3A** | **TF** | **Homo sapiens** | **0.5117** |
| RP00381026 | **LOC100507053** | **lncRNA** | **Homo sapiens** | **GABPA** | **TF** | **Homo sapiens** | **0.5117** |
| RP00374495 | **LOC100507053** | **lncRNA** | **Homo sapiens** | **FOXP2** | **TF** | **Homo sapiens** | **0.5117** |
| RP00366115 | **LOC100507053** | **lncRNA** | **Homo sapiens** | **JUND** | **TF** | **Homo sapiens** | **0.5117** |
| RP00359083 | **LOC100507053** | **lncRNA** | **Homo sapiens** | **JUNB** | **TF** | **Homo sapiens** | **0.5117** |
| RP00353860 | **LOC100507053** | **lncRNA** | **Homo sapiens** | **HDAC8** | **TF** | **Homo sapiens** | **0.5117** |
| RP00348853 | **LOC100507053** | **lncRNA** | **Homo sapiens** | **GATA2** | **TF** | **Homo sapiens** | **0.5117** |
| RP00334536 | **LOC100507053** | **lncRNA** | **Homo sapiens** | **CEBPB** | **TF** | **Homo sapiens** | **0.5117** |
| RP00330649 | **LOC100507053** | **lncRNA** | **Homo sapiens** | **GTF3C2** | **protein** | **Homo sapiens** | **0.5117** |
| RP00312029 | **LOC100507053** | **lncRNA** | **Homo sapiens** | **RELA** | **TF** | **Homo sapiens** | **0.5117** |
| RP00307900 | **LOC100507053** | **lncRNA** | **Homo sapiens** | **GTF2B** | **TF** | **Homo sapiens** | **0.5117** |
| RP00303675 | **LOC100507053** | **lncRNA** | **Homo sapiens** | **E2F1** | **TF** | **Homo sapiens** | **0.5117** |
| RP00285552 | **LOC100507053** | **lncRNA** | **Homo sapiens** | **E2F6** | **TF** | **Homo sapiens** | **0.5117** |
| RP00278418 | **LOC100507053** | **lncRNA** | **Homo sapiens** | **YY1** | **TF** | **Homo sapiens** | **0.5117** |
| RP00244583 | **LOC100507053** | **lncRNA** | **Homo sapiens** | **CTCF** | **TF** | **Homo sapiens** | **0.5117** |
| RP00225852 | **LOC100507053** | **lncRNA** | **Homo sapiens** | **HSF1** | **TF** | **Homo sapiens** | **0.5117** |
| RP00219305 | **LOC100507053** | **lncRNA** | **Homo sapiens** | **UBTF** | **TF** | **Homo sapiens** | **0.5117** |
| RP00210173 | **LOC100507053** | **lncRNA** | **Homo sapiens** | **MRE11** | **TF** | **Homo sapiens** | **0.5117** |
| RP00181211 | **LOC100507053** | **lncRNA** | **Homo sapiens** | **BRD4** | **TF** | **Homo sapiens** | **0.5117** |
| RP00160923 | **LOC100507053** | **lncRNA** | **Homo sapiens** | **CHD8** | **TF** | **Homo sapiens** | **0.5117** |
| RP00145382 | **LOC100507053** | **lncRNA** | **Homo sapiens** | **MYC** | **TF** | **Homo sapiens** | **0.5117** |
| RP00126484 | **LOC100507053** | **lncRNA** | **Homo sapiens** | **TAL1** | **TF** | **Homo sapiens** | **0.5117** |
| RP00106363 | **LOC100507053** | **lncRNA** | **Homo sapiens** | **SRF** | **TF** | **Homo sapiens** | **0.5117** |
| RP00101338 | **LOC100507053** | **lncRNA** | **Homo sapiens** | **SP1** | **TF** | **Homo sapiens** | **0.5117** |
| RP00090288 | **LOC100507053** | **lncRNA** | **Homo sapiens** | **SOX17** | **TF** | **Homo sapiens** | **0.5117** |
| RP00082259 | **LOC100507053** | **lncRNA** | **Homo sapiens** | **SNAI2** | **TF** | **Homo sapiens** | **0.5117** |
| RP00072979 | **LOC100507053** | **lncRNA** | **Homo sapiens** | **SMAD4** | **TF** | **Homo sapiens** | **0.5117** |
| RP00063318 | **LOC100507053** | **lncRNA** | **Homo sapiens** | **SMAD1** | **TF** | **Homo sapiens** | **0.5117** |
| RP00048558 | **LOC100507053** | **lncRNA** | **Homo sapiens** | **ZFP42** | **TF** | **Homo sapiens** | **0.5117** |
| RP00041271 | **LOC100507053** | **lncRNA** | **Homo sapiens** | **POU5F1** | **TF** | **Homo sapiens** | **0.5117** |
| RP00029241 | **LOC100507053** | **lncRNA** | **Homo sapiens** | **POLR2A** | **protein** | **Homo sapiens** | **0.5117** |
| RP00018634 | **LOC100507053** | **lncRNA** | **Homo sapiens** | **OTX2** | **TF** | **Homo sapiens** | **0.5117** |
| RP00009704 | **LOC100507053** | **lncRNA** | **Homo sapiens** | **NANOG** | **TF** | **Homo sapiens** | **0.5117** |
| RH00581147 | **LOC100507053** | **lncRNA** | **Homo sapiens** | **H3K4** | **histone modification** | **N/A** | **0.5117** |
| RH00556652 | **LOC100507053** | **lncRNA** | **Homo sapiens** | **H3K4me2-3** | **histone modification** | **N/A** | **0.5117** |
| RH00541731 | **LOC100507053** | **lncRNA** | **Homo sapiens** | **H3.3** | **histone modification** | **N/A** | **0.5117** |
| RH00517963 | **LOC100507053** | **lncRNA** | **Homo sapiens** | **H3K27me1** | **histone modification** | **N/A** | **0.5117** |
| RH00510573 | **LOC100507053** | **lncRNA** | **Homo sapiens** | **H3K9-14ac** | **histone modification** | **N/A** | **0.5117** |
| RH00502775 | **LOC100507053** | **lncRNA** | **Homo sapiens** | **H3K36me2** | **histone modification** | **N/A** | **0.5117** |
| RH00493384 | **LOC100507053** | **lncRNA** | **Homo sapiens** | **H3Ace** | **histone modification** | **N/A** | **0.5117** |
| RH00489128 | **LOC100507053** | **lncRNA** | **Homo sapiens** | **HIST2H3C** | **histone modification** | **N/A** | **0.5117** |
| RH00480695 | **LOC100507053** | **lncRNA** | **Homo sapiens** | **H4K16ac** | **histone modification** | **N/A** | **0.5117** |
| RH00472919 | **LOC100507053** | **lncRNA** | **Homo sapiens** | **H3Ac** | **histone modification** | **N/A** | **0.5117** |
| RH00450104 | **LOC100507053** | **lncRNA** | **Homo sapiens** | **H2AFZ** | **histone modification** | **N/A** | **0.5117** |
| RH00432042 | **LOC100507053** | **lncRNA** | **Homo sapiens** | **H3K9me1** | **histone modification** | **N/A** | **0.5117** |
| RH00419673 | **LOC100507053** | **lncRNA** | **Homo sapiens** | **H2AK9ac** | **histone modification** | **N/A** | **0.5117** |
| RH00400605 | **LOC100507053** | **lncRNA** | **Homo sapiens** | **H3T11ph** | **histone modification** | **N/A** | **0.5117** |
| RH00385050 | **LOC100507053** | **lncRNA** | **Homo sapiens** | **H2BK20ac** | **histone modification** | **N/A** | **0.5117** |
| RH00347330 | **LOC100507053** | **lncRNA** | **Homo sapiens** | **H4K12ac** | **histone modification** | **N/A** | **0.5117** |
| RH00335314 | **LOC100507053** | **lncRNA** | **Homo sapiens** | **H3K27me3** | **histone modification** | **N/A** | **0.5117** |
| RH00306029 | **LOC100507053** | **lncRNA** | **Homo sapiens** | **H3K23ac** | **histone modification** | **N/A** | **0.5117** |
| RH00297607 | **LOC100507053** | **lncRNA** | **Homo sapiens** | **H3K4ac** | **histone modification** | **N/A** | **0.5117** |
| RH00287445 | **LOC100507053** | **lncRNA** | **Homo sapiens** | **H2BK15ac** | **histone modification** | **N/A** | **0.5117** |
| RH00278801 | **LOC100507053** | **lncRNA** | **Homo sapiens** | **H4K91ac** | **histone modification** | **N/A** | **0.5117** |
| RH00267526 | **LOC100507053** | **lncRNA** | **Homo sapiens** | **H4K20me1** | **histone modification** | **N/A** | **0.5117** |
| RH00254035 | **LOC100507053** | **lncRNA** | **Homo sapiens** | **H4K8ac** | **histone modification** | **N/A** | **0.5117** |
| RH00242469 | **LOC100507053** | **lncRNA** | **Homo sapiens** | **H4K5ac** | **histone modification** | **N/A** | **0.5117** |
| RH00233337 | **LOC100507053** | **lncRNA** | **Homo sapiens** | **H3K79me2** | **histone modification** | **N/A** | **0.5117** |
| RH00225695 | **LOC100507053** | **lncRNA** | **Homo sapiens** | **H3K79me1** | **histone modification** | **N/A** | **0.5117** |
| RH00216684 | **LOC100507053** | **lncRNA** | **Homo sapiens** | **H3K56ac** | **histone modification** | **N/A** | **0.5117** |
| RH00203196 | **LOC100507053** | **lncRNA** | **Homo sapiens** | **H3K27ac** | **histone modification** | **N/A** | **0.5117** |
| RH00192038 | **LOC100507053** | **lncRNA** | **Homo sapiens** | **H3K23me2** | **histone modification** | **N/A** | **0.5117** |
| RH00173700 | **LOC100507053** | **lncRNA** | **Homo sapiens** | **H3K18ac** | **histone modification** | **N/A** | **0.5117** |
| RH00165060 | **LOC100507053** | **lncRNA** | **Homo sapiens** | **H3K14ac** | **histone modification** | **N/A** | **0.5117** |
| RH00151808 | **LOC100507053** | **lncRNA** | **Homo sapiens** | **H3K4me2** | **histone modification** | **N/A** | **0.5117** |
| RH00137470 | **LOC100507053** | **lncRNA** | **Homo sapiens** | **H2BK120ac** | **histone modification** | **N/A** | **0.5117** |
| RH00114009 | **LOC100507053** | **lncRNA** | **Homo sapiens** | **H2BK12ac** | **histone modification** | **N/A** | **0.5117** |
| RH00102623 | **LOC100507053** | **lncRNA** | **Homo sapiens** | **H2BK5ac** | **histone modification** | **N/A** | **0.5117** |
| RH00089374 | **LOC100507053** | **lncRNA** | **Homo sapiens** | **H2A.Z** | **histone modification** | **N/A** | **0.5117** |
| RH00077645 | **LOC100507053** | **lncRNA** | **Homo sapiens** | **H2AK5ac** | **histone modification** | **N/A** | **0.5117** |
| RH00053382 | **LOC100507053** | **lncRNA** | **Homo sapiens** | **H3K36me3** | **histone modification** | **N/A** | **0.5117** |
| RH00037507 | **LOC100507053** | **lncRNA** | **Homo sapiens** | **H3K9me3** | **histone modification** | **N/A** | **0.5117** |
| RH00027614 | **LOC100507053** | **lncRNA** | **Homo sapiens** | **H3K9ac** | **histone modification** | **N/A** | **0.5117** |
| RH00017368 | **LOC100507053** | **lncRNA** | **Homo sapiens** | **H3K4me3** | **histone modification** | **N/A** | **0.5117** |
| RH00004977 | **LOC100507053** | **lncRNA** | **Homo sapiens** | **H3K4me1** | **histone modification** | **N/A** | **0.5117** |
