# Supplementary material for: Circulating Long Non-Coding RNAs LINC00324 and LOC100507053 as Potential Liquid Biopsy Markers for Esophageal Squamous Cell Carcinoma: A Pilot Study
Source: Front Oncol. 2022 Feb 14;12:823953. doi: 10.3389/fonc.2022.823953 (PMC8882835; doi:10.3389/fonc.2022.823953)
Supplement: Supplementary file 7 [file DataSheet_1.docx]

**Supplementary File S1**

**Manuscript Title: -** Circulating long non-coding RNAs LINC00324 and LOC100507053 as potential liquid biopsy markers for esophageal squamous cell carcinoma: A pilot study

**Methodology**

**Next-Generation Sequencing and data processing**

Once the raw data were filtered, and combined transcripts were assembled on the hg19 genome, we identified lncRNAs from the combined target according to the following conditions: **1.** Filter the genes and lncRNA known to the database; **2.** RNA with a length higher than 200 nucleotides; **3.** Predictive open reading Frame (ORF) <300 nucleotides; **4.** Filtering RNA with coding potential. Through the above steps, the new transcripts with lncRNA characteristics were screened and finally recognized potential coding scores less than one as a new lncRNA by the CPC software (http://cpc.cbi.pku.edu.cn/) (17631615).

**LncRNA target prediction**

The parameter was set as organism “*Homo sapiens* (human),” ID type “Ensemble ID,” and Targets “miRNA” and ”mRNA” to search for the targets of *LINC00324* and *LOC100507053*.

**Gene Ontology (GO) and KEGG pathway analysis**

The parameter was set as organism “*Homo sapiens* (human),” Query gene “Targets of *LINC00324* and *LOC100507053*”, CLIP data ”high stringency (>=3)”, Program number “3”, Pathways “GO Biological pathway, GO, Molecular pathways, GO Cellular pathways and KEGG pathways”.
